# Structural basis of the stereoselective formation of the spirooxindole ring in the biosynthesis of citrinadins

**DOI:** 10.1038/s41467-021-24421-0

**Published:** 2021-07-06

**Authors:** Zhiwen Liu, Fanglong Zhao, Boyang Zhao, Jie Yang, Joseph Ferrara, Banumathi Sankaran, B. V. Venkataram Prasad, Biki Bapi Kundu, George N. Phillips, Yang Gao, Liya Hu, Tong Zhu, Xue Gao

**Affiliations:** 1 Department of Chemical and Biomolecular Engineering, Rice University, Houston, TX USA; 2 Department of Molecular Virology and Microbiology, Baylor College of Medicine, Houston, TX USA; 3 Rigaku Americas Corporation, The Woodlands, TX USA; 4 Department of Molecular Biophysics and Integrated Bioimaging, Berkeley Center for Structural Biology, Lawrence Berkeley National Laboratory, Berkeley, CA USA; 5 Verna and Marrs McLean Department of Biochemistry and Molecular Biology, Baylor College of Medicine, Houston, TX USA; 6 PhD Program in Systems, Synthetic, and Physical Biology, Rice University, Houston, TX USA; 7 Department of Biosciences, Rice University, Houston, TX USA; 8 Department of Chemistry, Rice University, Houston, TX USA; 9 Shanghai Engineering Research Center of Molecular Therapeutics & New Drug Development, School of Chemistry and Molecular Engineering, East China Normal University, Shanghai, China; 10 Department of Bioengineering, Rice University, Houston, TX USA

**Keywords:** X-ray crystallography, Enzyme mechanisms, Enzymes, Natural product synthesis

## Abstract

Prenylated indole alkaloids featuring spirooxindole rings possess a 3*R* or 3*S* carbon stereocenter, which determines the bioactivities of these compounds. Despite the stereoselective advantages of spirooxindole biosynthesis compared with those of organic synthesis, the biocatalytic mechanism for controlling the 3*R* or 3*S*-spirooxindole formation has been elusive. Here, we report an oxygenase/semipinacolase CtdE that specifies the 3*S*-spirooxindole construction in the biosynthesis of 21*R*-citrinadin A. High-resolution X-ray crystal structures of CtdE with the substrate and cofactor, together with site-directed mutagenesis and computational studies, illustrate the catalytic mechanisms for the possible β-face epoxidation followed by a regioselective collapse of the epoxide intermediate, which triggers semipinacol rearrangement to form the 3*S*-spirooxindole. Comparing CtdE with PhqK, which catalyzes the formation of the 3*R*-spirooxindole, we reveal an evolutionary branch of CtdE in specific 3*S* spirocyclization. Our study provides deeper insights into the stereoselective catalytic machinery, which is important for the biocatalysis design to synthesize spirooxindole pharmaceuticals.

## Abstract (web summary)

Prenylated indole alkaloids contain spirooxindole rings with a 3*R* or 3*S* carbon stereocenter, which determines their bioactivities, but the biocatalytic mechanism controlling the 3*R*- or 3*S*-spirooxindole formation was unclear. Here, the authors report the biochemical and structural characterization of the oxygenase/semipinacolase CtdE that catalyses the 3*S*-spirooxindole construction in the biosynthesis of 21*R*-citrinadin A.

## Introduction

The efficacy and safety of chiral pharmaceuticals often critically depend on their specific stereochemistry. Therefore, the asymmetric synthesis of chiral molecules is important in pharmaceutical research and development^1^. While it remains very challenging in organic synthesis to rigidly control the stereochemistry of small molecules with multiple stereocenters, nature has evolved many fascinating enzymes catalyzing stereoselective chemical transformations^2,3^. Discovering new biocatalysts featuring high stereoselectivity and understanding their molecular mechanisms will provide new insights into powerful biocatalyst development for the manufacture of structurally complex pharmaceuticals.

**Fig. 1:**
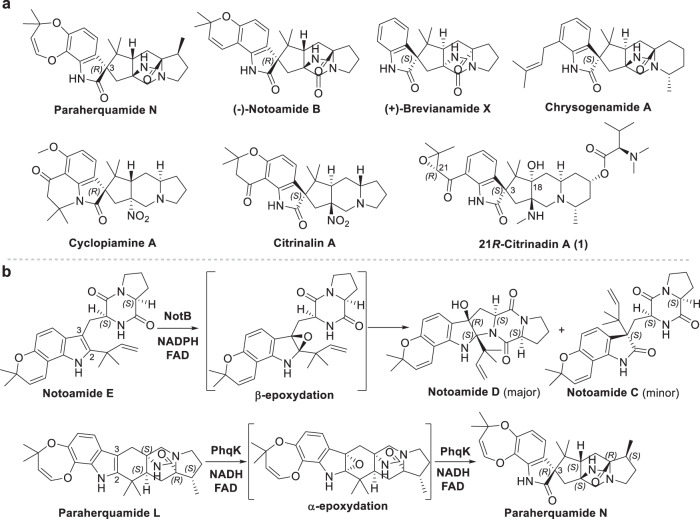
Representative 3*R*- and 3*S*-spirooxindole PIAs and FPMOs. **a** Spirooxindole PIAs contain or lack the bicyclo[2.2.2]diazaoctane ring. **b** FPMO-catalyzed facial selective indole 2,3-epoxidations on PIAs.

The spirooxindole ring is present in a variety of bioactive natural products and has been increasingly utilized as a promising pharmacophore in drug discovery^4,5^. Prenylated indole alkaloids (PIAs) featuring characteristic spirooxindole scaffolds possess great structural and bioactive diversity^6^. Among them, the anthelmintic paraherquamides^7^, anticancer notoamides^8^, and insecticidal brevianamides^9^ represent one class of PIAs that bears the bicyclo[2.2.2]diazaoctane ring system, while the other class lacks such an extended ring system, including anticancer agents cyclopiamines^10^ and citrinidins^11^ (Fig. 1a and Supplementary Fig. 1). Both classes of PIAs have different spiro systems, 3*R* and 3*S* spiro rings (Fig. 1a). As a promising scaffold for drug discovery, the stereochemical restriction on the spiro center (3*R* or 3*S*) could not only achieve specific binding to their respective targets, but also potentially improve drug oral bioavailability and metabolic stability^12,13^. Moreover, the three-dimensional structure of spiro scaffolds in spirooxindoles plays a critical role in their bioactivities^14^. However, enantioselective and efficient construction of chiral spirooxindole frameworks is very challenging in organic synthesis^15^. For example, in the total synthesis of citrinalin congeners (Fig. 1a), an improved Davis’ oxaziridine-catalyzed reaction to the desired 3*S*-spirooxindole showed only up to 52% yield, while another catalyst mediated the formation of the 3*R*-spirooxindole congener in 56% yield with a diastereomeric ratio of 4:1 (ref. ^16^).

In the biosynthesis of spirooxindole PIAs, amino acid precursors are initially assembled by the multidomain nonribosomal peptide synthetases (NRPSs)^17^. Subsequently, various enzymatic modifications, including the construction of spirocycles, further enrich their structural complexity^18^. The flavoprotein monooxygenases (FPMOs)^19^ are widespread and perform a variety of oxygenations, including hydroxylations^20^, Baeyer–Villiger oxidations^21^, and epoxidations^22^, each incorporating a single oxygen atom into the substrate. A few FPMOs have been reported to perform indole 2,3-epoxidation in the biosynthesis of PIAs^23–26^, which could act before or after the formation of the bicyclo[2.2.2]diazaoctane ring by the intramolecular [4 + 2] hetero-Diels–Alder (IMDA) reaction (Supplementary Fig. 2). NotB has been reported to catalyze the formation of non-spirocyclized notoamides C and D through a presumed indole 2,3-β-epoxide intermediate^22^, which does not contain the bicyclo[2.2.2]diazaoctane ring (Fig. 1b). Another FPMO, PhqK, could perform a specific α-face epoxidation on the bicyclo[2.2.2]diazaoctane substrates, triggering semipinacol rearrangement to build up 3*R*-spirooxindole constructions after the IMDA cyclization^24^ (Fig. 1b).

Citrinadin A was first discovered from a marine-derived *Penicillium citrinum* strain and exhibits notable activity against murine leukemia L1210 and human epidermoid carcinoma KB cells^11^. Citrinadin A possesses a unique 6/5/5/6/6 pentacyclic ring core with the addition of an *N*, *N*-dimethylvaline ester unit and an α, β-epoxy-carbonyl moiety. This complex structure with multiple stereocenters has served as a fascinating target in subsequent synthetic studies^27–31^. The absolute configuration of citrinadin A was corrected to be 3*S* spirocycle via the first enantioselective total synthesis in 2013 (refs. ^31,32^, Fig. 1a), the same as citrinalin^16^ and chrysogenamide A^33^ (Fig. 1a). A recent biosynthetic study has demonstrated that (2*S*, 6*S*)-6-methyl pipecolate is a key precursor in building up the l-pipecolate moiety in citrinadin A^34^. However, the later biosynthetic steps for citrinadins, including the formation of the spirooxindole ring, remain elusive. Unlike paraherquamides^24^, notoamides^23^, and brevianamides^25^, citrinadins do not contain the bicyclo[2.2.2]diazaoctane ring. Notably, a close examination of the stereocenters in citrinadins showed that they feature 3*S*-spirooxindole, which is opposite to those in paraherquamides^24^, indicating the possible presence of unique stereocontrol for the 3*S* spirocycle formation in citrinadin biosynthesis.

Herein, we report the identification of a distinct FPMO, CtdE, that stereoselectively catalyzes the 3*S*-spirooxindole formation in the 21*R*-citrinadin A (**1**) biosynthesis. Based on thorough analyses of the high-resolution X-ray crystal structures of CtdE complex containing substrate and cofactor flavin adenine dinucleotide (FAD), together with the site-directed mutagenesis and computational study, we revealed the molecular basis for the stereoselective catalytic mechanism that CtdE exploits for the possible β-facial epoxidation, triggering semipinacol rearrangement to yield 3*S*-spirooxindole PIAs. Our discovery of the stereoselective formation of the 3*S* spirocycle and deciphering of the mechanistic details of CtdE are important in the spirooxindole pharmaceutical research and development.

## Results

### Characterization of the biosynthetic intermediates reveals the function of CtdE

**Fig. 2:**
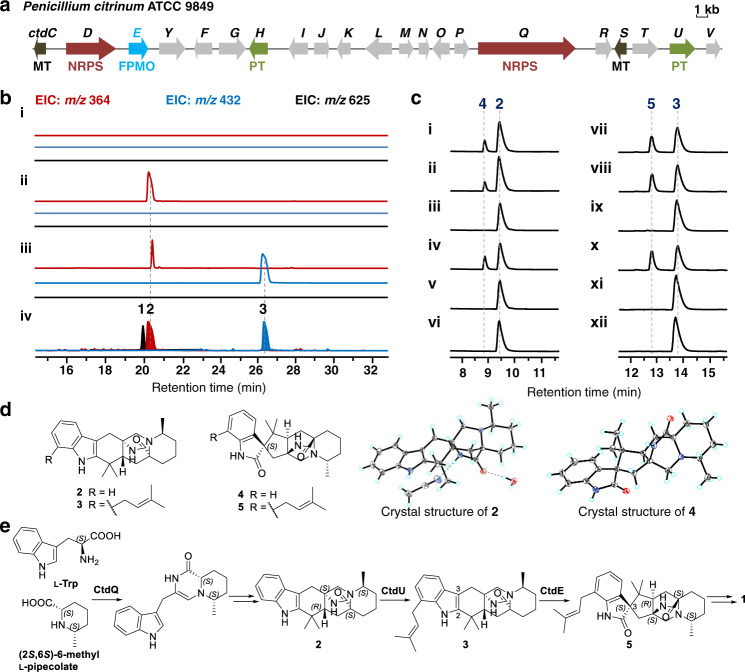
Functional characterization of CtdE and the proposed biosynthetic pathway. **a** Putative gene cluster for **1** biosynthesis in *P. citrinum*. **b** LCMS analysis (extracted ion chromatogram, EIC) of **1** production in *P. citrinum* wild-type (WT) and *ctd* mutants: (i) Δ*ctdQ*, (ii) Δ*ctdU*, (iii) Δ*ctdE*, (iv) standards of compounds **1**–**3**. EIC traces of *m/z* 364 (red), *m/z* 432 (blue), and *m/z* 625 (black) are in different colors. **c** In vitro assays of CtdE. (i) **2** + CtdE + NADH + FAD, (ii) **2** + CtdE + NADH, (iii) **2** + CtdE + FAD, (iv) **2** + CtdE + NADPH + FAD, (v) **2** + NADPH + FAD, (vi) **2** + NADH + FAD, (vii) **3** + CtdE + NADH + FAD, (viii) **3** + CtdE + NADH, (ix) **3** + CtdE + FAD, (x) **3** + CtdE + NADPH + FAD, (xi) **3** + NADH + FAD, and (xii) **3** + NADPH + FAD. **d** Chemical structures of **2**–**5**, and X-ray crystal structures of **2** and **4**. **e** Proposed biosynthetic pathway of **1**. (LCMS elution methods for *ctd* mutants extract and in vitro assays are different, see details in Methods).

Fermentation of *P. citrinum* ATCC 9849 led to the discovery of a major secondary metabolite, 21*R*-citrinadin A (**1**, Fig. 1a). The structure and absolute configuration of **1** were confirmed by extensive NMR and electric circular dichroism (ECD) analyses, which are consistent with the total synthetic 21*R*-citrinadin A^31^ (Supplementary Table 4 and Supplementary Fig. 3). We then searched the genome of *P. citrinum* ATCC 9849 to uncover the biosynthetic gene cluster corresponding to **1** (Supplementary Fig. 4). Related to the reported PIAs’ gene clusters (*mal*, *phq*, and *not*/*not*′)^35^, a putative biosynthetic cluster *ctd* for **1** was revealed, which includes two NRPSs (CtdQ and CtdD), two prenyltransferases (PTs, CtdH and CtdU), two methyltransferases (MTs, CtdS and CtdC), and an FPMO (CtdE; Fig. 2a). When compared to the recently discovered citrinadin A (*cnd*)^34^ gene cluster (Supplementary Fig. 4), the *ctd* cluster here contains additional NRPS (*ctdD*) and MT (*ctdC*) genes. To unequivocally link the *ctd* gene cluster to the production of **1**, we first deleted the putative dipeptide NRPS gene *ctdQ*, using split-marker recombination approach^36^ (Supplementary Fig. 5). The production of **1** was completely abolished in Δ*ctdQ* mutant, which confirmed that the *ctd* gene cluster is responsible for the biosynthesis of **1** (Fig. 2a).

Bioinformatics analyses suggested that CtdE is homologous to previously reported FPMOs NotI^23^ (45.2%), NotB^22^ (37.8%), PhqK^24^ (34.3%), and FqzB^37^ (35.0%). Both NotI and PhqK have been characterized as α-face epoxidases for semipinacol rearrangements in the spirocycle formation^23,24^ (Supplementary Fig. 2), while NotB catalyzes β-face epoxidation of notoamide E to generate the non-spirocyclized notoamide C^22^ (Fig. 1b). Therefore, CtdE was predicted to be involved in the spirooxindole formation of **1**. Knockout of the *ctdE* gene led to the accumulation of **2** (*m/z* 364.2, [M + H]^+^) and a primary product **3** (*m/z* 432.3, [M + H]^+^) compared with the wild-type (WT) strain (Fig. 2b, and Supplementary Figs. 6 and 7). To determine the absolute stereochemistry, we obtained the X-ray crystal structure of **2** with a good Flack parameter of −0.01(3) (CCDC 2057621, Fig. 2d), demonstrating that **2** possesses a bicyclo[2.2.2]diazaoctane ring. **3** was identified as a C7-prenyl substituted derivate from **2** by NMR spectroscopy (Supplementary Table 6). The ECD spectrum of **3** is well-matched with **2**, allowing us to determine that its absolute configuration is the same as **2** (Supplementary Fig. 3). Therefore, we proposed that CtdE may utilize **2** or **3** as the substrate, and catalyze the 3*S* spirocycle formation. Notably, both **2** and **3** contain the characteristic bicyclo[2.2.2]diazaoctane framework, which indicates that spirocyclization takes place after the bicyclo[2.2.2]diazaoctane ring formation in the biosynthesis of **1**. This also implied that later tailoring steps deconstructed the bicyclo[2,2,2]diazaoctane framework, since it was missing in the final structure of **1**.

CtdU is a putative PT, and its homologs MalE^38^ (41.4% identity) and NotF^39^ (37.1% identity) were previously characterized to catalyze C2 reverse prenylated reaction in malbrancheamide and notoamide biosynthesis, respectively. LCMS metabolites analysis revealed one major intermediate **2** (*m/z* 364.2, [M + H]^+^, Fig. 2b) and three other minor metabolites **u1**–**u3** accumulated in the Δ*ctdU* mutant (Supplementary Figs. 6 and 7). Intermediate **u1** was identified as spirooxindole PIA product lacking a C7-prenyl group by NMR and ECD analyses, while **u2** and **u3** were also proposed to be spirooxindole PIAs without a C7 prenylation based on their UV and MS spectra (Supplementary Figs. 4, 6, and 7, and Supplementary Table 9). Thus, CtdU was deduced to be responsible for normal prenylation at C7 position rather than C2 position.

### In vitro characterization of the CtdE

To verify the exact function of CtdE, we expressed and purified this enzyme as a *C*-His-tagged protein from *Escherichia coli* BL21(DE3) (Supplementary Fig. 8). The purified CtdE protein shows a yellow color, indicating that the FAD could bind to the protein. When we incubated 500 μM **2** with 2 μM CtdE, 5 mM NADH, and 100 μM FAD in Tris-HCl (pH 7.6) buffer, >30% of **2** was converted to a new product **4** (*m/z* 380.2 [M + H]^+^) within 2 h (Fig. 2c, traces i and vi). When **3** was incubated with CtdE in the same condition, product **5** (*m/z* 448.3 [M + H]^+^) with a similar UV spectrum as **4** was also produced with a higher conversion rate (Fig. 2c, traces vii and xii, and Supplementary Fig. 7). Both nicotinamide adenine dinucleotide (NADH) and nicotinamide adenine dinucleotide phosphate (NADPH) could be utilized as the cofactor for CtdE catalysis (Fig. 2c, traces i, iv, vii, and x). The addition of exogenous FAD to the reactions could improve the product yield (Fig. 2c, traces i, ii, vii, and viii). After large-scale in vitro assays of **2** and **3** with CtdE, respectively, enough amounts of **4** and **5** were purified for structural elucidation. The absolute structure of **4** was determined by single-crystal X-ray diffraction analysis with Cu Kα radiation (CCDC 2057622, Fig. 2d), confirming the 3*S*-spirooxindole configuration in **4**. Based on NMR and ECD analyses, the structure of **5** was elucidated to be chrysogenamide A^33^, a C7-prenyl substituted derivative from **4** (Fig. 2d). Furthermore, Michaelis–Menten kinetics analyses indicated that **3** is the favored substrate for CtdE to generate spirooxindole product due to a significantly higher catalytic efficiency (*k*_cat_/*K*_M_ = 27.9 ± 3.9 min^−1^ mM^−1^, Supplementary Fig. 9b) as compared to **2** (*k*_cat_/*K*_M_ = 3.8 ± 0.5 min^−1^ mM^−1^, Supplementary Fig. 9a). These results demonstrate that CtdE could stereoselectively catalyze the formation of 3*S*-spirooxindoles of **4** and **5** from substrates **2** and **3**, respectively. Therefore, we concluded that CtdU firstly mediated C7 prenylation from **2** to **3**, followed by CtdE catalyzed spirocyclization to generate 3*S*-spirooxindole **5** (Fig. 2e). We proposed that **3** may undergo a 2,3-β-face epoxidation as the first key step catalyzed by CtdE and followed by the regioselective opening of the epoxide ring that triggers the semipinacol rearrangement to form the 3*S*-spirooxindole **5**. Notably, CtdE revealed a divergent evolutionary process in PIAs biosynthesis (Supplementary Fig. 10). In the *mal* biosynthetic pathway, (+)-premalbrancheamide is synthesized via an IMDA reaction^40^; further biosynthesis of spirooxindole rings was not found in the *mal/mal*′ gene cluster^38,41^. In contrast, PhqK transforms the bicyclo[2,2,2]diazaoctane substrate to 3*R*-spirooxindole^24^. Here, we show that CtdE catalyzes the β-face epoxidation of substrate **3** to construct a 3*S* spirocycle. Moreover, the *bvn* system represents another biosynthetic branch, in which BvnB catalyzes the β-face epoxidation of deoxybrevianamide E to generate 3β-hydroxyindolenine product before the bicyclo[2,2,2]diazaoctane ring formation^25^.

### The overall crystal structures of CtdE–FAD and CtdE–FAD-3 complexes

**Fig. 3:**
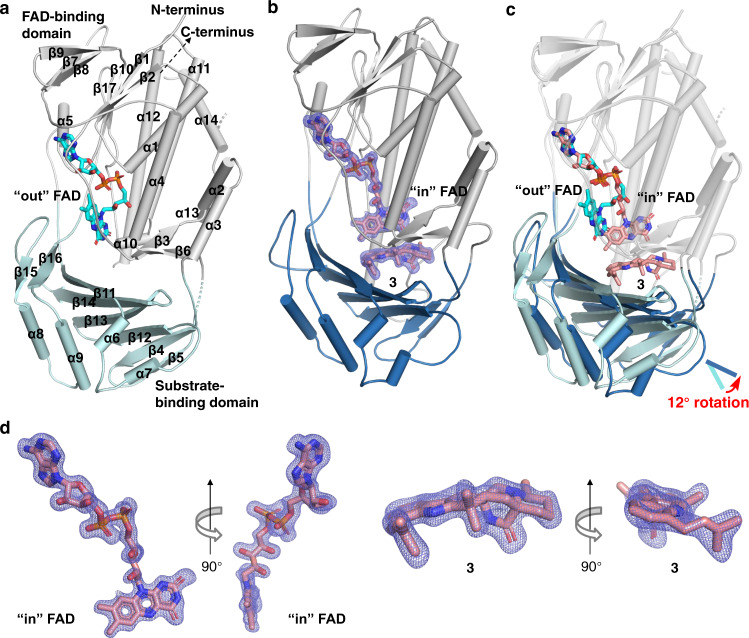
The overall CtdE crystal structures and structural comparisons. **a** The overall structure of the CtdE–FAD complex shown in a cartoon model with two domains (gray and pale cyan), and an FAD (cyan carbons and shown in a stick representation) in the “out” conformation. **b** The overall structure of the CtdE–FAD-**3** complex is shown in a cartoon model with two domains (gray and sky blue). The simulated annealing omit map (slate mesh, *F*_o_*–F*_c_, contoured at 3.0 σ) indicates binding of “in” FAD and **3** (pink carbons and shown in a stick representation) at the canonical binding site. **c** Superimposed CtdE–FAD and CtdE–FAD-**3** complex structures aligned with FAD-binding domains (gray), show the different conformations of substrate-binding domains (pale cyan and sky blue, respectively). Aligned FAD molecules are respectively in the “out” and “in” conformations (cyan and pink carbons, respectively, are shown in a stick representation). **d** The simulated annealing omit maps of the “in” FAD and **3** as shown in Fig. 3b.

**Fig. 4:**
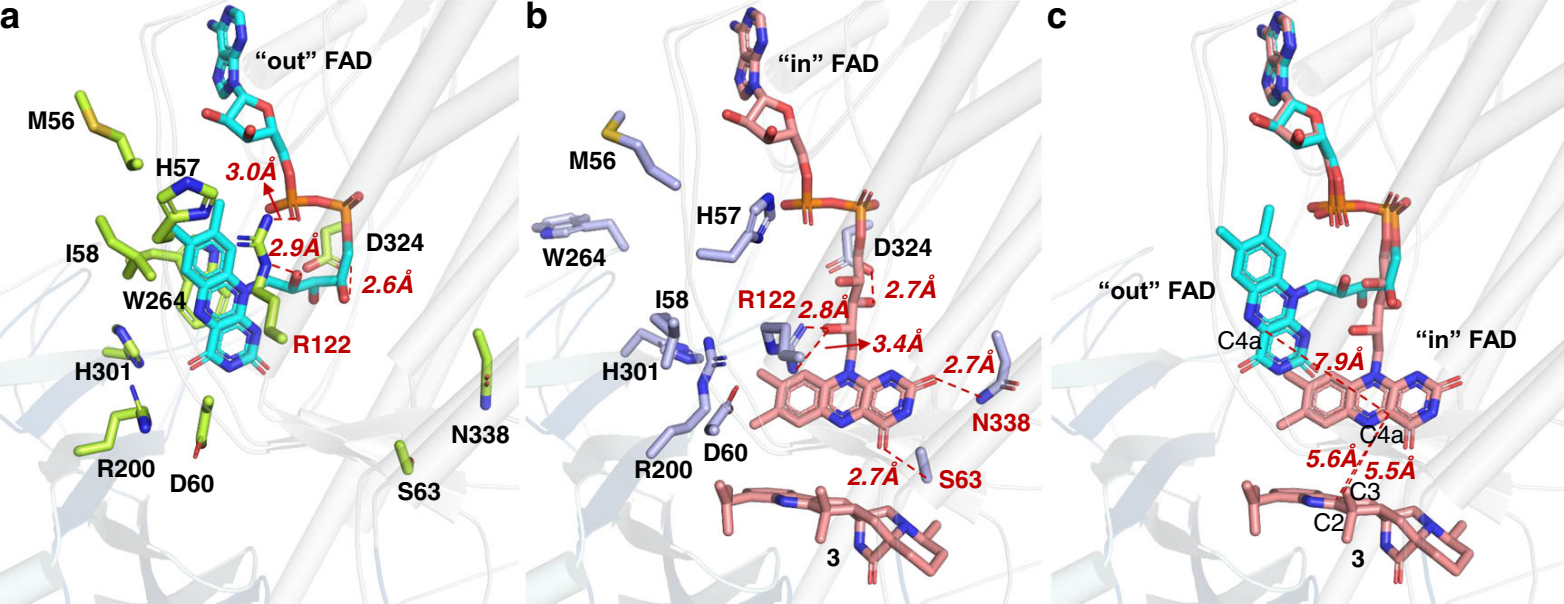
Structural changes of the FAD-binding sites in CtdE upon substrate binding. **a** “Out” FAD-binding site. **b** “In” FAD-binding site. **c** Structural overlay of “out” FAD and “in” FAD-binding sites.

To gain insights into the mechanism of the stereoselective spirocyclization catalyzed by CtdE, the X-ray crystal structures of CtdE–FAD and CtdE–FAD-**3** complexes were solved by molecular replacement, and refined to 2.1 and 1.9 Å resolution, respectively (Supplementary Table 12). CtdE comprises two domains, a three-layer ββα sandwich domain for FAD binding, and an internal substrate-binding domain featuring eight antiparallel β-sheets (Fig. 3a). CtdE belongs to group A FPMOs, which do not contain a separate dinucleotide-binding domain^19^. Similar domain structures were seen in related FPMOs PhqK^24^ (PDB: 6pvi), PhzS^42^ (PDB: 2rgj), and 3HB6H^43^ (PDB: 4bk3), the highest-ranked structural homologs of CtdE, according to the DALI structure server^44^ (Supplementary Table 13 and Supplementary Fig. 11). The cofactor FAD was co-purified with CtdE without exogenous supplement during the protein purification and crystallization steps. In the CtdE–FAD complex, the FAD exists in the “out” conformation, in which the isoalloxazine moiety is away from the substrate-binding site, similar to that seen in PhqK^24^. Interestingly, the FAD undergoes a major conformational change from the “out” to the “in” conformation with the C4a carbon moved 7.9 Å upon substrate binding (Figs. 3b and 4c), which was not obtained in the previous PhqK structures^24^. Similar to the “mobile flavin” in *para*-hydroxybenzoate hydroxylase study^45^, the “out” conformation could enable flavin reduction and substrate releasing, while the “in” conformation places flavin adjacent to the substrate and performs oxidation reactions. The “in” and “out” conformations of FAD in CtdE likely play important dynamic roles for substrate binding and product release during the catalytic cycle^46^. Substrate **3** is positioned at the domain–domain interface beneath the isoalloxazine ring of FAD (Fig. 3b). Besides the “out” to “in” transition of FAD, the internal β-sheets domain rotates 12° (Fig. 3c) toward substrate **3** upon substrate binding, pushing it toward the FAD. The CtdE–FAD complex features an open substrate loading channel, allowing the substrate to access the active site (Supplementary Fig. 12a), whereas this substrate loading channel closed after substrate binding in the CtdE–FAD-**3** structure (Supplementary Fig. 12b). The simulated annealing omit map for unbiased electron density within the active site allows us to determine the absolute stereochemistry of **3** (Fig. 3b). Importantly, our result revealed that FAD was well positioned on the β-face of substrate **3**, indicating CtdE could catalyze a β-face epoxidation of the substrate.

### CtdE catalysis and the active site

The FAD in its “out” and “in” conformations displays drastic changes in its interactions with surrounding residues (Fig. 4a, b). The residue R122 forms different hydrogen bonds with the hydroxy groups of flavin in the “out” and the “in” conformations, and is conserved in FPMOs PhzS (R106)^42^, HpxO (R103)^47^, and TropB (R124)^48^. N338 and S63 form hydrogen bonds with the isoalloxazine ring of FAD in the “in” conformation to stabilize FAD in a catalytic position. Thus, these residues are suggested to play an important role in directing FAD-binding and conformational conversion. Referring to the other group A FPMOs^45,48,49^, the conformational change of the flavin upon substrate binding could allow for the reduction of FAD by the reductant NADH/NADPH. Along with FAD conformational change, the residues, such as W264, I58, H57, H301, and R200, also show drastic changes of positions to match the mobile flavin moiety. To explore how the “in” FAD interacts with the substrate, we further determine its distance with the substrate in the CtdE–FAD-**3** complex (Fig. 4c). The C4a atom of the flavin in the “in” position is close to the C2 and C3 of **3** with distances of 5.6 and 5.5 Å, respectively (Fig. 4c). These distances are appropriate for the proposed C(4a)-hydroperoxide flavin (Fl_OOH_)^50^, which has been characterized as an active intermediate for oxygen activation in many group A FPMOs^19^, to perform epoxidation on C2=C3 bond in substrate **3**. Similarly, in FPMOs HpxO^47^ and TetX^20^, the distances from the C4a of the flavin to the hydroxylation sites of the substrates are 4.9 and 5.9 Å in the “in” conformation, respectively.

**Fig. 5:**
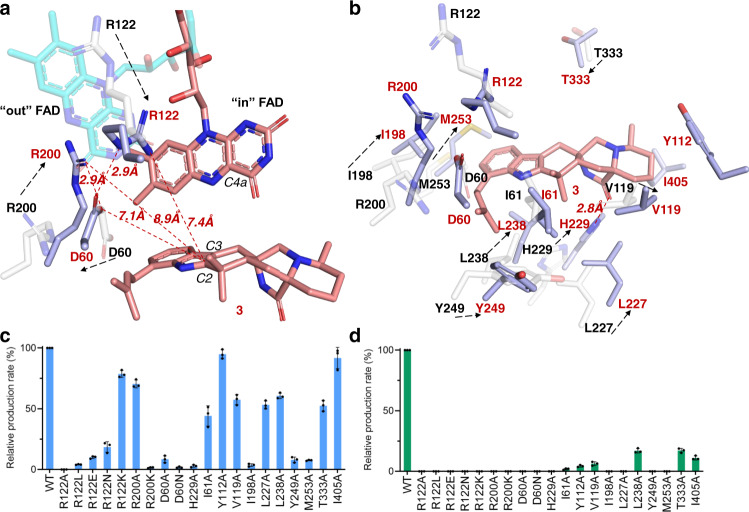
Active site and substrate-binding site views of CtdE–FAD-3 complex and its substrate-free structure. **a** Overlay of the active site view of CtdE–FAD (residue carbons colored in white, FAD carbons colored in cyan) and CtdE–FAD-**3** (residue carbons colored in light blue and FAD carbons colored in red) complexes. **b** Overlay of substrate-binding site view of CtdE–FAD (residues carbons colored in white) and CtdE–FAD-**3** (residues carbons colored in light blue and carbons of compound **3** colored in red) complexes. **c** Analysis of the relative production rate of **3** with CtdE mutants. **d** Analysis of the relative production rate of **2** with CtdE mutants. Data represent the average of triplicate independent experiments (center values, mean; error bars, s.d.; *n* = 3).

The major residues surrounding the active site of substrate **3** are shown in Fig. 5a, b. Among them, residues R200 and D60 are conserved and correspond to R192 and D47 in PhqK^24^, respectively. R192 is proposed as a general acid to catalyze epoxide opening in PhqK^24^. In our CtdE–FAD-**3** complex, two arginines, R200 and R122, are bridged by the negatively charged D60 (Fig. 5a). The amine groups of R122 and R200 are 9.3 and 7.4 Å away from the C2 of **3**, respectively (Fig. 5a). To determine the catalytic residues for the epoxide intermediate protonation in CtdE, we further perform site-directed mutagenesis of CtdE to probe the catalytic mechanism. The enzyme activities of the purified CtdE mutants were assayed in vitro, using **3** and **2** as the substrate, respectively (Fig. 5c, d). The R200A mutant retains 70.4 ± 3.2% activity of the CtdE WT with **3**, indicating that R200 may not serve as a catalytic residue in CtdE. In addition, R200 is in a similar position to R220 in PHBH^49^ and R206 in TropB^48^, which are both proposed to be involved in the reduction of FAD. The R220K mutant of PHBH is found to stabilize the conformation of “out” FAD and substantially decreases the catalytic efficiency of PHBH^49^, which may explain why the mutant R200K of CtdE almost completely abolished the enzymatic activity with **3**, possibly due to stabilizing the noncatalytic “out” FAD conformation. The R122A mutant completely abolished the enzyme activity with either **3** or **2**, while mutants R122L, R122E, R122N, and R122K abolished the activity with **2**, but retained 4.1 ± 0.2%, 10.0 ± 1.0%, 18.5 ± 4.3%, and 78.6 ± 2.9% activity of CtdE with **3**, respectively. These results indicate that R122 is crucial for catalysis and that positively charged lysine (K) also supports the function of R122. Thus, we propose that R122 may play multiple roles in CtdE catalysis, including orienting and stabilizing the “in” FAD conformation as proposed functions of R106 in PhzS^42^, and participating in directing the epoxide intermediate collapse that is similar to the putative function of R192 in PhqK^24^. Mutants of D60A and D60N only keep 8.5 ± 2.9% and 1.8 ± 0.6% activities of CtdE, suggesting that D60 may play an important role in both FAD reduction and substrate protonation by stabilizing with R200 and R122, respectively.

### Probing the stereoselective mechanism of CtdE

The structure of the CtdE–FAD-**3** complex displays a hydrophobic binding pocket to stabilize the poorly water-soluble substrate (Supplementary Fig. 13). Residue H229 forms a typical hydrogen bond interaction (2.8 Å) with the carboxylate oxygen in **3** (Fig. 5b). The CtdE variant H229A retains only 2.9 ± 1.0% activity with **3** (Fig. 5c), indicating the hydrogen bond interaction has a significant effect on stabilizing the substrate. A set of nonpolar residues, such as I61, V119, L227, L238, M253, T333, and I405, contributing to hydrophobic interactions with substrate **3** could help the substrate keeping an appropriate binding pose (Fig. 5a). Our mutagenesis study showed that the enzymatic activities of these mutants are greatly diminished with **2**, but less diminished with the favored substrate **3**, suggesting that the prenyl group in **3** may greatly attribute to the binding affinity with CtdE^51^ (Fig. 5c, d). Superimposing the CtdE–FAD complex onto the CtdE–FAD-**3** revealed that the residues R122, R200, H229, L238, M253, and T333 move closer to **3** upon substrate binding (Fig. 5b). In addition, the polar residues D60 and Y249 move away from the substrate, which may provide a hydrophobic environment for substrate binding.

**Fig. 6:**
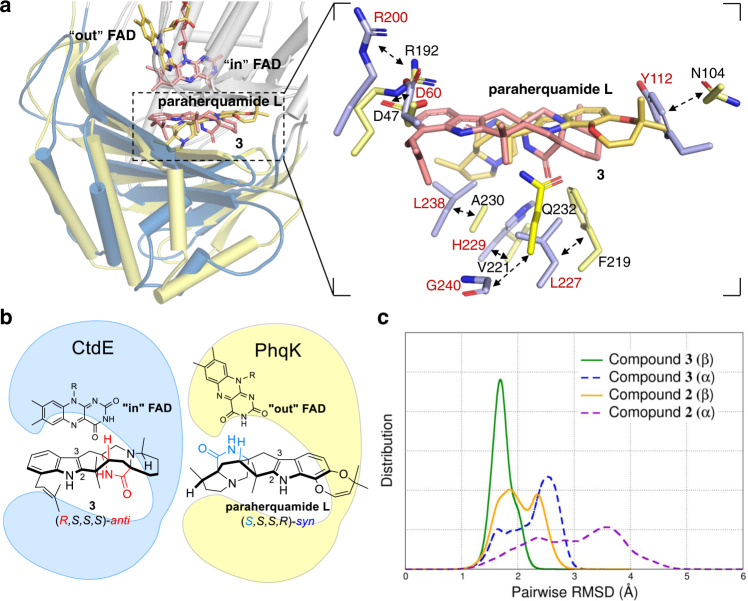
Comparison of the different facial selectivity in CtdE and PhqK. **a** Overlay structures of CtdE–FAD-**3** and PhqK–FAD–substrate complexes and their substrate-binding view of **3** (**3** is shown as red carbons, while paraherquamide L is shown as orange carbons. CtdE is shown in blue, while PhqK is shown in yellow). **b** Comparison of the stereochemistry of CtdE and PhqK substrates. **c** The pairwise RMSD of protein backbone atoms over time of CtdE substrates **2** and **3**, respectively. (β and α represent the positions of FAD on the β- and α-face of the substrate, respectively).

To confirm the difference of facial selectivity in CtdE and PhqK^24^, PhqK was expressed and purified (Supplementary Fig. 8) to respectively perform in vitro assays with **2** and **3**. Our results showed PhqK could not react with **2** or **3** in vitro (Supplementary Fig. 14). We then carefully compared the crystal structures and substrate-binding pockets of CtdE and PhqK (Fig. 6a). Structural comparison of **3** in CtdE and paraherquamide L in PhqK shows two major differences. First, **3** possesses an *anti*-configuration of bicyclo[2,2,2]diazaoctane framework with an *S*-methyl pipecolate ring, while paraherquamide L features a *syn*-configuration of bicyclo[2,2,2]diazaoctane framework fused to an *R*-methyl pyrrolidine ring (Fig. 6b). Notably, the difference in configurations of bicyclo[2,2,2]diazaoctane framework results in a significant change in the 3D structures of molecules. Second, the binding position of **3** in the CtdE complex turns nearly 180°, with respect to the paraherquamide L in PhqK (Fig. 6b), and the substrate-binding domain of CtdE also exhibits a significant difference from those in PhqK. Residue H229 in CtdE shows a hydrogen bond interaction with the carboxyl group of **3**, L238 in CtdE has a π–sigma interaction with the indole unit, and Y112 in CtdE has a π–sigma interaction with the pipecolate ring of **3**. However, the corresponding residues V221, A230, and N104 in PhqK are not conserved and lose the ability to bind substrate for β-facial selectivity. Residue Q232 in PhqK possesses a strong hydrogen bond with indole NH of the substrate paraherquamide L, and F219 has a π–π interaction with the indole unit of the substrate. On the contrary, CtdE loses these interactions by having G240 and L227 instead of Q232 and F219 in PhqK, respectively.

To further elucidate the stereoselectivity of CtdE catalysis, 500 ns classical molecular dynamics (MD) simulations were performed for CtdE with substrates **3** and **2**, respectively. Pairwise root mean square deviations (RMSDs)^52^, measuring the conformational variability among the sampled conformations, were calculated for the CtdE β-system (FAD is on the β-face of the substrate) and the CtdE α-system (FAD is on the α-face of the substrate). The results showed RMSD distribution of the β-system with **2** and **3** are smaller than those of the α-system (Fig. 6c), indicating that the β-system is more stable than the α-system in CtdE. Representative binding poses of substrates **2** and **3** with FAD in MD simulations are shown in Supplementary Fig. 15. Moreover, the calculated binding free energy (∆*G*_bind_) of the β-face poses of **3** (∆*G*_bind_ = −73.78 kcal mol^−1^) and **2** (∆*G*_bind_ = −55.48 kcal mol^−1^) are 6.26 and 15.42 kcal mol^−1^ lower than their α-face pose, respectively (Supplementary Table 14). These results further support that β-facial selectivity of substrates is preferable in the CtdE catalytic pocket. Among them, **3** shows a stronger binding affinity than **2** to CtdE, consistent with our mutagenesis study (Fig. 5c, d). As expected, the hydrophobic interactions (∆*E*_vdw_) contribute the most to the binding affinity. To explore the hot-spot residues of the substrate binding, per-residue free energy decomposition was performed. The results showed that the residues L238, H229, L227, R200, and I405 provide the major contribution to substrate binding in the CtdE active pocket (Supplementary Fig. 16).

**Fig. 7:**
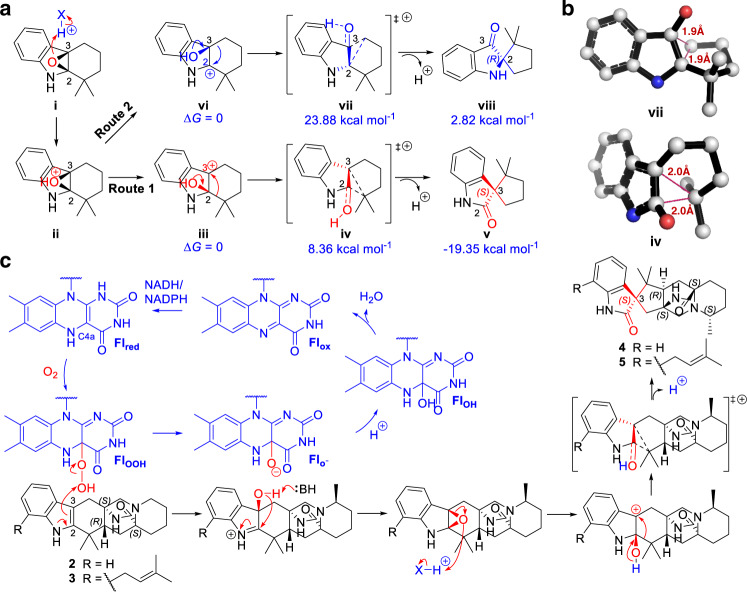
DFT calculations of the transformation pathways of CtdE. **a** Proposed pathways of the general acid-catalyzed epoxide opening and semipinacol rearrangement, and the corresponding calculated Gibbs energies. **b** The transition-state structures of the β-C2-hydroxyl carbocations (**iv**) and β-C3-hydroxyl carbocations (**vii**). **c** Proposed catalytic mechanism of CtdE for the transformation of **2** and **3** (Fl_red_, Fl_OOH_, Fl_O_^−^, Fl_OOH_, and Fl_ox_ represent reduced flavin, C4a-hydroperoxide flavin, C4a-peroxide flavin, C4a-hydroxide flavin, and oxidized flavin, respectively. X represents arginine or H_2_O).

To explore how the β-epoxide intermediate transforms to the 3*S*-spirooxindole product, we performed density functional theory (DFT) calculations (see Methods) to evaluate the intermediates and transition state (TS). The truncated indole fragment was modeled as the substrate, which was similarly performed in the PhqK calculation^24^. A proton was provided to represent the general acid catalyst, while the 2,3-β-epoxy intermediate **i** was set as an initial substrate. Subsequent protonation of **i** leads to the epoxide opening to generate predicted C2-hydroxyl carbocation intermediate **iii** (route 1) and C3-hydroxyl carbocation intermediate **vi** (route 2), respectively (Fig. 7a). Then, preferable migration of the alkyl moiety from C2 to C3 (route 1) and from C3 to C2 (route 2) through the less hindered α-face could yield the 3*S* and 2 *R* spirooxindole products, respectively. Accordingly, route 1 to 3*S*-spirooxindole is a preferable pathway due to the lower Gibbs energy of the TS **iv** (8.36 kcal mol^−1^) than that of **vii** (23.88 kcal mol^−1^) in route 2 (Fig. 7a, b). Moreover, residues R122/D60, which could direct the collapse of the epoxide, are located on the indole C2 side of the molecule as opposed to the C3 side (Fig. 5a).

## Discussion

Understanding how nature utilizes enzymes in the stereoselective synthesis of bioactive small molecules could greatly benefit the discovery and development of chiral drugs. In this study, we revealed a 3*S* stereoselective spirocyclization step in **1** biosynthesis via functional gene inactivation and in vitro assay studies. A oxygenase/semipinacolase CtdE was characterized for this spirocycle formation with a 3*S* stereoselective characteristic. A high-resolution X-ray crystal structure of CtdE–FAD–substrate complex, together with mutagenesis and MD simulations, fully support a β-face selective epoxidation of the indole C2=C3 bond followed by the regioselective collapse of the epoxide ring and semipinacol rearrangement to generate the 3*S*-spirooxindole construction. Our study revealed a divergent evolutionary process for spirocyclization in PIAs biosynthesis (Supplementary Fig. 10). CtdE enriches the structural complexity and diversity of PIAs products as an evolutionary branch that catalyzes specific 3*S* spirocyclization after the IMDA process.

FqzB is the only example of the FPMOs reported to be responsible for the 3*S* spirocyclic scaffold biosynthesis in spirotryprostatins^37^, which lack a bicyclo[2.2.2]diazaoctane framework (Supplementary Fig. 2). However, the catalytic mechanism of FqzB was proposed to be 2,3-α-face epoxidation followed by semipinacol rearrangement^37,53^. It was unclear how the FPMO catalyzes 3*S*-spirooxindole formation in the PIAs prior to our study. To the best of our knowledge, CtdE is the first reported FPMO biocatalyst that is responsible for the formation of the 3*S*-spirooxindole framework by 2,3-β-face epoxidation triggering semipinacol rearrangement (Fig. 7c). The CtdE–FAD-**3** complex structure reveals that the “in” FAD in the catalytic site is properly positioned and is on the β-face of the substrate to enable β-facial selectivity.

We have determined and examined the key amino acids contributing to the β-facial selectivity of CtdE via specific hydrophobic and hydrogen interactions. The MD simulations enabled us to understand the molecular basis for the β-facial preference of CtdE. The crystallographic analyses combined with site-directed mutagenesis suggested that the H229 and a set of hydrophobic residues in the active pocket help the substrate stay in a proper position for β-facial epoxidation. Residue R122 is proposed to play a key role in FAD-binding and conformation change due to its hydrogen bond interactions with FAD. Based on our mutagenesis study, R122 may serve as the proton donor to collapse the epoxide from the C3 side by protonation, leading to the regioselective collapse of the epoxide (Fig. 7c). Despite the long distance between R122 and substrate **3** in the crystal structure, a conformational change of these residues may occur after the formation of the unstable 2,3-β-epoxide intermediate. Alternatively, the water molecule between R122/D60 and the substrate may serve as an appropriate media for the proton transfer in the active site instead of the direct protonation from the residues R122/D60 (Supplementary Fig. 17). Quantum chemistry calculations were performed to evaluate the β-epoxide intermediate transformation to the spirooxindole products through a reasonable TS under a general acid catalyst. Accordingly, the route 1 to 3*S*-spirooxindole product is indeed a preferable pathway.

The discovery of the indole C2 = C3 epoxide shunt products in the in vitro studies of several homologous FPMOs, such as Af12060 (ref. ^50^), NotB^22^, and FqzB^37^, suggested the possible existence of these 2,3-epoxide intermediates to form the final spirooxindole products. However, it is worth mentioning that there is no direct evidence for the transient 2,3-epoxide intermediate formation in CtdE assays (Fig. 7c). We next checked if the direct formation of a C2-hydroxyl carbocation intermediate **iii** by the C4a-hydroperoxy flavin oxygenation, without a preceding epoxide intermediate, is also possible in CtdE catalysis (Supplementary Fig. 18). We performed the DFT calculations to compare the Gibbs free energies required to form the C2 or C3-hydroxyl intermediates. Our results indicated that the C3-hydroxyl intermediate **xi** might be more favorable to be formed than the C2-hydroxyl **iii**, since the truncated C3-hydroxyl TS (**x**, Δ*G*^‡^ = 31.44 kcal mol^−1^) is 3.18 kcal mol^−1^ lower in energy than that of the truncated C2-hydroxyl TS (**xii**, Supplementary Fig. 18). However, such a C2-hydroxyl **iii** is required to trigger the downstream semipinacol rearrangement and generate the final 3*S*-spirooxindole product (Fig. 7c). Thus, our DFT results indicate the direct hydroxylation in the C2 position is less likely and a preceding epoxide intermediate could be needed. In addition to the FPMOs, two cytochrome P450 monooxygenases, *Aspergillus*-derived FtmG^37^ and actinomycete-derived CyaH^54^, could also catalyze the formation of spirooxindole through a proposed radical-mediated hydroxylation and semipinacol rearrangement.

In conclusion, our work highlights a biocatalytic tool for chemoenzymatic diversification of PIAs biosynthesis with a specific β-facial selectivity to construct the 3*S*-spirooxindole ring. The mechanistic insight gained from our research will provide opportunities for the development of stereospecific catalysts and promising applications in spirooxindole drug design.

## Methods

### Strains, chemicals, and molecular biology agents

*P. citrinum* ATCC 9849 was purchased from ATCC (https://www.atcc.org/). *E. coli* TOP10 was used as the host for DNA preparation. *E. coli* BL21(DE3) was used for protein expression. Primers for *E. coli* expression were synthesized by Integrated DNA Technologies, Inc. (Coralville, USA). DNA sequencing was performed at the GENEWIZ, Inc. (New Jersey, USA). Kits for plasmid preparation and DNA isolation were purchased from Vazyme Biotech Co., Ltd (Nanjing, CN). Standard molecular biology methods were used for all other DNA manipulations.

### Inactivation of *ctd* genes in *P. citrinum* ATCC 9849

Split-marker homologous recombination^36^ was used for *ctd g*ene inactivations in *P. citrinum* ATCC 9849 (Supplementary Fig. 5). The homologous regions (~1.5 kb) and the hygromycin resistant marker gene *hyg* were PCR amplified from *P. citrinum* ATCC 9849 genome and plasmid pUCH2-8, respectively. DNA fragments were assembled into a *pUC57-Amp* vector using Gibson assembly. Then PCR amplification and gel purification were performed for the preparation of DNA fragments for homologous recombination. Polyethylene glycol (PEG)-mediated recombination was performed^55^ similarly, as previously reported. Briefly, spores of *P. citrinum* ATCC 9849 grew overnight to obtain the conidia. Then the protoplasts were obtained after 4 h of digestion (shake at 100 RPM, 30 °C) of the conidia by yatalase (2 mg mL^−1^) and lysing enzyme (3 mg mL^−1^). The protoplasts were further centrifuged and resuspended to a concentration of 10^8^–10^9^. After that, the prepared DNA fragments (10 µg) were incubated with the protoplast for 50 min at 4 °C, and subsequently mixed with PEG solution to spread on the hygromycin-containing plates (150 mg L^−1^). The plates were incubated at 30 °C for 4 days. Finally, the correct mutants were screened by colony PCR, and the colony PCR result was illustrated in Supplementary Fig. 5. Primers used for mutant screening are listed in Supplementary Table 2.

### Compound isolation, purification, and identification

The WT *P. citrinum* and mutants were cultured on 2 L YES medium at 28 °C for 5 days. The cells were extracted with ethyl acetate three times and the extracts were evaporated to dryness. The crude extracts were then isolated by silica chromatography, the fractions containing target compounds were collected, and the solvent was removed by rotary evaporation. The target fractions were further purified by Sephadex LH-20 (40–70 μm; GE Healthcare Life Science, USA) chromatography. The obtained subfractions were purified by Prep-HPLC (Agilent 1260 with DAD-detector) equipped with a semi-preparative Ultimate XB-C18 column (10 × 250 mm, 5 µm, Welch, China). A linear gradient of 40–80% acetonitrile (v/v) over 30 min in H_2_O (0.01% triethylamine, v/v) at a flow rate of 4 mL min^−1^ was used for compounds purification. The resulting compounds were collected and dried for NMR analysis. NMR spectra were recorded on a Bruker NEO 600 MHz High-Performance Digital NMR (BrukerBiospin, Sweden), using CDCl_3_ solvent (Cambridge Isotope Laboratories, USA). High-resolution mass spectrometry (HRMS) was performed on an Agilent 1290 Infinity/6230 TOF LCMS system, using electrospray ionization in positive mode.

### Protein expression and purification

The gene *ctdE* was amplified from the cDNA of *P. citrinum* and cloned into pET29 vector with a C-terminal hexa-histidine tag. After sequencing verification, the plasmids were transformed into *E. coli* BL21(DE3) for protein expression. *E. coli* cells were cultured in 1 L LB broth containing 100 μg mL^−1^ ampicillin at 37 °C until the optical density (OD_600_) value reached 0.5, and then protein expression was induced with 0.24 mM IPTG for 14 h at 16 °C. All purification steps were conducted at 4 °C. The cells were harvested by centrifugation (4000 × *g*) for 20 min, and then resuspended in 30 mL lysis buffer (50 mM Tris-HCl, 300 mM NaCl, 5 mM imidazole, and 1.0 mM TCEP, pH 8.0.) and lysed by sonication. Subsequently, high-speed centrifugation (12,000 × *g*, 30 min) was applied to obtain the lysate soluble fraction. The soluble fraction was added to 0.5 mL of Ni-NTA resin (QIAGEN) for protein binding (2 h), and then the mixture was loaded onto a gravity-flow column. Proteins were washed with washing buffer (50 mM Tris-HCl, 300 mM NaCl, 20 mM imidazole, and 1.0 mM TCEP, pH 8.0) and eluted with elution buffer (50 mM Tris-HCl, 300 mM NaCl, 300 mM imidazole, and 1.0 mM TCEP, pH 8.0). The elution buffer containing the purified proteins was finally exchanged with the exchange buffer (50 mM Tris-HCl, 300 mM NaCl, 10% glycerol, and 1.0 mM TCEP, pH 8.0) and concentrated. The obtained proteins were used for in vitro assay and stored at −80 °C (Supplementary Fig. 8). Source data of SDS–PAGE gels for purified proteins are provided as a Source data file. For the protein crystallization experiment, CtdE was further purified by size-exclusion chromatography on a Superdex 75 Increase 10/300 GL (GE Healthcare) column with 20 mM Tris, pH 8.0, 100 mM NaCl, and 1 mM dithiothreitol. The concentration of the purified CtdE was determined by measuring absorbance at 280 nm and using an absorption coefficient of 67,380 M^−1^ cm^−1^ calculated using ProtParam on the ExPASy server.

### In vitro activity assay

The standard enzyme assay containing 100 μM FAD, 500 μM substrate, 5 mM NADPH, and 2 μM enzyme in 50 μL reaction buffer (50 mM Tris-HCl, pH 7.6) was performed at 28 °C for 2 h. The reactions were quenched with 50 μL LCMS grade methanol and centrifuged to remove solid material. The samples were analyzed on an Agilent 6120B Single Quadrupole LCMS using an Agilent Poroshell 120 EC-C18 column (3.0 × 150 mm) with the following time program: 5–95% acetonitrile over 25 min, 95% acetonitrile for 5 min, 95–5% acetonitrile over 1 min, and 5% acetonitrile for 4 min. A 0.1% of formic acid was added to H_2_O. The flow rate was 0.5 mL min^−1^, and the reactions were monitored at 254 nm.

### Mutagenesis of CtdE

Primers for *ctdE* mutagenesis were ordered from IDT. After PCR amplification and gel purification, the mutated DNA fragments were cloned into pET29 vectors using Gibson assembly. The mutant plasmids were verified by DNA sequencing and transformed into *E. coli* BL21(DE3) for protein expression. *E. coli* cells were cultured in 0.5 L LB medium to an OD_600_ value of 0.5. Protein expression was induced with 0.24 mM IPTG for 13–16 h at 16 °C. Cells were collected by centrifugation and were resuspended in 30 mL lysis buffer (50 mM Tris-HCl, 300 mM NaCl, 5 mM imidazole, and 1.0 mM TCEP, pH 8.0). After sonication, the lysis mixture was centrifuged at 12,000 × *g* (4 °C, 30 min) to remove cell debris. A total of 0.5 mL of Ni-NTA resin was added to clear cell lysate for protein binding. Proteins were washed with washing buffer (50 mM Tris-HCl, 300 mM NaCl, 20 mM imidazole, and 1.0 mM TCEP, pH 8.0) and eluted with elution buffer (50 mM Tris-HCl, 300 mM NaCl, 300 mM imidazole, and 1.0 mM TCEP, pH 8.0). Finally, eluted samples containing pure proteins were exchanged with an exchange buffer (50 mM Tris-HCl, 300 mM NaCl, 10% glycerol, and 1.0 mM TCEP, pH 8.0) and stored at −80 °C (Supplementary Fig. 8). Source data of SDS–PAGE gels for purified proteins are provided as a Source data file. The conversion and the relative activities of CtdE mutants were measured by the relative product rates compared with the WT CtdE. The error bars represent the standard deviation (s.d.) of three independent replicates. The concentration of products **4** and **5** were estimated by standard carves of **4** and **5** that were generated from peak areas at 254 nm (UV) by HPLC. The data are shown in Fig. 5c, d and Supplementary Fig. 9.

### Kinetic assay

To determine the kinetic parameters of CtdE, the reactions were performed in 50 µL reaction containing Tris-HCl buffer (50 mM, pH = 7.6), 2 μM CtdE, 5 mM NADH, 200 μM FAD, and 20–1000 μM substrate (**2** or **3**) at 28 °C. Reactions were quenched by adding equal volume cold methanol at 10, 15, and 20 min, respectively. The quenched samples were analyzed on an Agilent Technologies 6120 Quadrupole LCMS (with UV-detector) using Agilent Eclipse Plus C18 column (4.6 × 100 mm) for quantitative analyses. Kinetic data fitting was performed using GraphPad Prism 8. *K*_M_, *k*_cat_, and *k*_cat_/*K*_M_ values represent the mean ± s.d. of three independent replicates. The result is shown in Supplementary Fig. 9.

### Crystallization and single-crystal X-ray diffraction analyses of compounds 2 and 4

Colorless needles of compounds **2** and **4** were crystallized from a CH_3_CN solution and a CHCl_3_/CH_3_OH solution by slow evaporation at room temperature, respectively. Data were collected on a Rigaku Oxford Diffraction XtalLAB Synergy-S using Cu Kα radiation at Rigaku Corp. Using Olex2 (ref. ^56^), the structure was solved with the SHELXT structure solution program using Intrinsic Phasing^57^, and refined with the SHELXL refinement package using least squares minimization^58^. The crystallographic data have been deposited at the Cambridge Crystallographic Data Centre with deposition numbers CCDC 2057621 for **2** and CCDC 2057622 for **4**. The detailed data and final refinement of **2** and **4** are presented in Supplementary Tables 10 and 11, respectively.

### Crystallization and structure determination of CtdE

Crystallization screenings of CtdE with co-purified FAD at a protein concentration of 11 mg mL^−1^ were carried out by hanging-drop vapor diffusion using the Mosquito crystallization robot (TTP LabTech) and visualized by 9901 stereo zoom microscope (Carl Zeiss) at 25 °C. The CtdE in complex with FAD was crystallized with Molecular Dimensions Morpheus II crystallization screen ID 2-30 (100 mM amino acids II, 0.1 M buffer system 5, pH 7.5, and 32.5% v/v precipitant mix 6). For co-crystallization of CtdE in complex with substrate **3**, the CtdE with 4.5 mg mL^−1^ was diluted to 15 mL with 20 mM Tris (pH 8.0), 100 mM NaCl, and 1 mM dithiothreitol. A total of 20 μM substrate **3** in 100% dimethyl sulfoxide was added to the CtdE with a final 40-fold excess molar concentration relative to CtdE. The complex was concentrated to 14 mg mL^−1^ after 1-h incubation at 4 °C. The CtdE in complex with substrate **3** was crystallized with 0.1 M calcium chloride dihydrate, 20% w/v PEG6000, and 10% v/v ethylene glycol. The crystals were 0.1–0.2 mm Cryoloops (Hampton Research, USA) and flashed frozen directly in liquid nitrogen.

X-ray diffraction data for the CtdE crystals were collected on beamline 5.0.2 at Advanced Light Source, Lawrence Berkeley National Laboratory. The data were processed with IMOSFLM as implemented in the CCP4 suite^59^. Space groups were confirmed using POINTLESS^60^. The crystal structure of PhqK (PDB ID: 6PVI) was used as a search model for molecular replacement using PHASER^61^. The atomic model was then subjected to refinement using PHENIX^62^, and further model building using COOT^63^ based on the different maps. Data collection and refinement statistics following the final refinement cycle are given in Supplementary Table 12. The structural alignments and calculations of RMSD were carried out using the Chimera^64^. Figures were generated using PyMOL (https://pymol.org/2/).

### Molecular dynamics simulation

The 3D structures of FAD, **2**, and **3** were optimized at the AM1 level. Then the Antechamber module in the Amber 18 package was used to assign the BBC charges for them. The Amber *ff14SB* force field was assigned for the protein, while the general Amber force field (*gaff2*) was assigned for FAD, **2**, and **3**. The protein–ligand complexes were solvated in a truncated octahedral water box with a buffer of 12 Å, and the TIP3P model was assigned for water molecules. Na^+^ ions were added to neutralize the system.

To remove bad contacts in the initial structures of four complex systems, each of them was minimized for 3000 steps (1000 steps with the steepest descent algorithm followed by 2000 steps with the conjugate gradient algorithm). Then each system was heated gradually from 0 to 300 K within 1 ns. After the heating process, 1 ns equilibrium simulation was performed at 300 K to further relax the system. Subsequently, 500 ns MD simulation was conducted for each system and the structures were recorded at a time interval of 4 ps. To further improve the sampling efficiency, a time step of 2 fs was used. The SHAKE algorithm was used to restrain chemical bonds with hydrogen atoms. The Langevin thermostat was used to control the temperature during the simulation, and the collision frequency was set to 2.0 ps^−1^. The particle mesh Ewald method was used to treat the long-range electrostatic interactions and the non-bond cutoff was set to 12 Å. All the MD simulations were performed on NVIDIA 2080TI GPUs with the *pmemd.cuda* module in the Amber 18 software. Pairwise RMSDs, which can reflect the conformational variability among the sampled conformations, were calculated by using the MDAnalysis software^52^.

### Binding free energy calculation

Molecular mechanics generalized born surface area was used to calculate the binding affinity of CdtE with compounds **2** and **3**. ∆*G*_bind_ = ∆*G*_comp_ − ∆*G*_pro_ − ∆*G*_lig_ = ∆*E*_ele_ + ∆*E*_vdW_ + ∆*G*_pol_ + ∆*G*_nonpol_ − *T*∆*S* (1), where ∆*G*_comp_, ∆*G*_pro_, and ∆*G*_lig_, represent free energies of the complex, protein, and substrates, respectively. The two terms ∆*E*_ele_ and ∆*E*_vdW_ are electrostatic and van der Waals interactions of substrates with proteins, respectively. ∆*G*_pol_ and ∆*G*_nonpol_ are the polar and nonpolar solvation free energies, among which ∆*G*_pol_ can be computed with the GB model and ∆*G*_nonpol_ is solved with the empirical equation: ∆*G*_nonpol_ = γ − ∆SASA + β (2), where the parameter γ − ∆SASA represents the surface tension and the difference in the solvent-accessible surface areas induced by substrate bindings. The parameter γ and β were set to 0.0072 kcal mol Å^−2^ and 0 kcal mol^−1^ in our work, separately. We neglected the calculation of the entropy change because the structures of the compounds in this work are relatively similar and the normal mode of calculation is very time-consuming.

### Quantum chemical calculation

All quantum mechanical calculations were performed with Gaussian 16. Geometry optimizations were calculated with the M06-2X density functional and the 6-31G(d) basis set. Single point energies were calculated using M06-2X and the 6-311++G(d,p) basis set. The solvent effect was taken into the PCM model.

### Reporting summary

Further information on research design is available in the Nature Research Reporting Summary linked to this article.

## Supplementary information

Supplementary Information

## Data Availability

Data supporting this work are available within the paper and the supplementary files. All additional data supporting the current study in the article or its supplementary files are available from the corresponding author upon request. Coordinates and associated structure factors of CtdE have been deposited in the Protein Data Bank (PDB) with accession codes 7KPQ and 7KPT. The crystallographic data of small molecules have been deposited at the Cambridge Crystallographic Data Centre with deposition numbers CCDC 2057621 for **2** and CCDC 2057622 for **4**. Energies and molecular coordinates of calculated structures are provided in the Supplementary Information file.  Source data are provided with this paper.

## Source data

Source Data
