# Supplementary material for: Structural basis of the stereoselective formation of the spirooxindole ring in the biosynthesis of citrinadins
Source: Nat Commun. 2021 Jul 6;12:4158. doi: 10.1038/s41467-021-24421-0 (PMC8260726; doi:10.1038/s41467-021-24421-0)
Supplement: Supplementary file 1 — Supplementary Information [file 41467_2021_24421_MOESM1_ESM.docx]

**Supplementary Information**

Structural basis of the stereoselective formation of the spirooxindole ring in the biosynthesis of citrinadins

Zhiwen Liu^1,11^, Fanglong Zhao^1,11^, Boyang Zhao^2,11^, Jie Yang^1^, Joseph Ferrara^3^, Banumathi Sankaran^4^, B. V. Venkataram Prasad^2,5^, Biki Bapi Kundu^6^, George N. Phillips, Jr.^7,8^, Yang Gao^7^, Liya Hu^5^, Tong Zhu^9*^ & Xue Gao^1,8,10*^

^1^Department of Chemical and Biomolecular Engineering, Rice University, Houston, Texas 77005, USA.

^2^Department of Molecular Virology and Microbiology, Baylor College of Medicine, Houston, Texas 77030, USA.

^3^Rigaku Americas Corporation, 9009 New Trails Drive, The Woodlands, Texas 77381, USA.

^4^Department of Molecular Biophysics and Integrated Bioimaging, Berkeley Center for Structural Biology, Lawrence Berkeley National Laboratory, Berkeley, California, 94720, USA.

^5^Verna and Marrs McLean Department of Biochemistry and Molecular Biology, Baylor College of Medicine, Houston, Texas 77030, USA.

^6^PhD Program in Systems, Synthetic, and Physical Biology, Rice University, Houston, Texas 77005, USA.

^7^Department of Biosciences, Rice University, Houston, Texas 77005, USA.

^8^Department of Chemistry, Rice University, Houston, TX 77005, USA.

^9^Shanghai Engineering Research Center of Molecular Therapeutics & New Drug Development, School of Chemistry and Molecular Engineering, East China Normal University, Shanghai, China.

^10^Department of Bioengineering, Rice University, Houston, Texas 77005, USA.

^11^These authors contributed equally.

*e-mail: [xue.gao@rice.edu](mailto:xue.gao@rice.edu); [tzhu@lps.ecnu.edu.cn](mailto:tzhu@lps.ecnu.edu.cn)

**Table of Contents**

[Supplementary Tables S4](#_Toc60637238)

**Supplementary Table 1** Strains and plasmids in this study. S4

**Supplementary Table 2** Primers used in this study. S5

**Supplementary Table 3** BLASTP CtdE homologs in NCBI databases. S7

**Supplementary Table 4** NMR data of **1** and 21*R*-citrinadin A in reference (*δ* in ppm, *J* in Hz). S9

**Supplementary Table 5** NMR data of compound **2** (*δ* in ppm, *J* in Hz). S10

**Supplementary Table 6** NMR data of compound **3** (*δ* in ppm, *J* in Hz). S11

**Supplementary Table 7** NMR data of compound **4** (*δ* in ppm, *J* in Hz). S12

**Supplementary Table 8** NMR data of compound **5** (*δ* in ppm, *J* in Hz). S13

**Supplementary Table 9** NMR data of compound **u1** (*δ* in ppm, *J* in Hz). S14

**Supplementary Table 10** Crystal data and structure refinement for compound **2**. S15

**Supplementary Table 11** Crystal data and structure refinement for compound **4**. S16

**Supplementary Table 12** Crystallographic data collection and structure refinement statistics. S17

**Supplementary Table 13** Structural homologs of CtdE identified by DALI server. S18

**Supplementary Table 14** Calculation of the binding free energy of β-face pose and α-face pose of substrates in CtdE. S19

Supplementary Figures S20

**Supplementary Fig. 1** Representative prenylated indole alkaloids. S20

**Supplementary Fig. 2** FPMOs of NotB, PhqK, NotI/NotI’, BvnB and FqzB catalyzed reactions. S21

**Supplementary Fig. 3** ECD spectra analysis of **1-5**. S22

**Supplementary Fig. 4** Comparison analysis of the biosynthetic gene clusters of PIAs. S23

**Supplementary Fig. 5** PCR confirmation of *ctd* mutants. S24

**Supplementary Fig. 6** LCMS traces of metabolic extracts from △*ctdU* mutant, △*ctdE* mutant and wild-type of *P. citrinum.* S25

**Supplementary Fig. 7** UV and MS spectra of compounds **1**-**5** and **u1**. S26

**[Supplementary Fig. 8](#_Toc60637260)** [SDS-PAGE (12%) analysis of purified CtdE, PhqK and CtdE site-directed mutant enzymes. S](#_Toc60637260)27

**Supplementary Fig. 9** Kinetic analysis of CtdE catalyzed reactions of **2** and **3**. S28

**Supplementary Fig. 10** Summary of biosynthetic pathways in PIAs reveal evolutionary branches. S29

**Supplementary Fig. 11** Alignment of CtdE and its structural homolog PhqK. S30

**Supplementary Fig. 12** The cut-way view of surface representation of CtdE-FAD and CtdE-FAD-**3** complexes. S31

**Supplementary Fig. 13** Electrostatic surface potential of CtdE-FAD-**3** complex. S32

**Supplementary Fig. 14** *In vitro* assays of CtdE and PhqK. S33

**Supplementary Fig. 15** Overlay of 10 representative binding poses from 500 ns MD simulations of **3** and **2**. S34

**Supplementary Fig. 16** Per-residue free energy decomposition of **3**. S34

[**Supplementary Fig. 17** The water molecule 290 in the active side of CtdE-FAD-**3** structure. S35](#_Toc60637270)

**Supplementary Fig. 18** DFT calculated Gibbs free energies for the C2 and C3 hydroxylation steps by using the truncated structures. S36

[**Supplementary Fig. 19-1**^1^H NMR spectrum of **1** in CDCl_3_. S3](#_Toc60637271)7

[**Supplementary Fig. 19-2**^13^C NMR spectrum of **1** in CDCl_3_. S3](#_Toc60637272)8

[**Supplementary Fig. 19-3** DEPT135 and ^13^C NMR spectra of **1** in CDCl_3_. S3](#_Toc60637273)9

[**Supplementary Fig. 19-4** ^1^H-^1^H COSY NMR spectrum of **1** in CDCl_3_. S40](#_Toc60637274)

[**Supplementary Fig. 19-5** HSQC NMR spectrum of **1** in CDCl_3_. S41](#_Toc60637275)

[**Supplementary Fig. 19-6** HMBC NMR spectrum of **1** in CDCl_3_. S4](#_Toc60637276)2

[**Supplementary Fig. 19-7** ROESY NMR spectrum of **1** in CDCl_3_. S43](#_Toc60637277)

[**Supplementary Fig. 20-1** ^1^H NMR spectrum of **2** in CDCl_3_. S44](#_Toc60637278)

[**Supplementary Fig. 20-2** ^13^C NMR spectrum of **2** in CDCl_3_. S45](#_Toc60637279)

[**Supplementary Fig. 20-3** DEPT135 and ^13^C NMR spectra of **2** in CDCl_3_. S4](#_Toc60637280)6

[**Supplementary Fig. 20-4** ^1^H-^1^H COSY NMR spectrum of **2** in CDCl_3_. S47](#_Toc60637281)

[**Supplementary Fig. 20-5** HSQC NMR spectrum of **2** in CDCl_3_. S48](#_Toc60637282)

[**Supplementary Fig. 20-6** HMBC NMR spectrum of **2** in CDCl_3_. S49](#_Toc60637283)

[**Supplementary Fig. 20-7** NOESY NMR spectrum of **2** in CDCl_3_. S50](#_Toc60637284)

[**Supplementary Fig. 20-8** HRMS spectrum of **2**. S51](#_Toc60637284)

[**Supplementary Fig. 21-1** ^1^H NMR spectrum of **3** in CDCl_3_. S52](#_Toc60637285)

[**Supplementary Fig. 21-2** ^13^C NMR spectrum of **3** in CDCl_3_. S53](#_Toc60637286)

[**Supplementary Fig. 21-3** DEPT135 and ^13^C NMR spectra of **3** in CDCl_3_. S54](#_Toc60637287)

[**Supplementary Fig. 21-4** ^1^H-^1^H COSY NMR spectrum of **3** in CDCl_3_. S55](#_Toc60637288)

[**Supplementary Fig. 21-5** HSQC NMR spectrum of **3** in CDCl_3_. S56](#_Toc60637289)

[**Supplementary Fig. 21-6** HMBC NMR spectrum of **3** in CDCl_3_. S57](#_Toc60637290)

[**Supplementary Fig. 21-7** NOESY NMR spectrum of **3** in CDCl_3_. S58](#_Toc60637291)

[**Supplementary Fig. 21-8** HRMS spectrum of **3**. S59](#_Toc60637284)

[**Supplementary Fig. 22-1** ^1^H NMR spectrum of **4** in CDCl_3_. S60](#_Toc60637292)

[**Supplementary Fig. 22-2** ^13^C NMR spectrum of **4** in CDCl_3_. S61](#_Toc60637293)

[**Supplementary Fig. 22-3** DEPT135 and ^13^C NMR spectra of **4** in CDCl_3_. S62](#_Toc60637294)

[**Supplementary Fig. 22-4** ^1^H-^1^H COSY NMR spectrum of **4** in CDCl_3_. S63](#_Toc60637295)

[**Supplementary Fig. 22-5** HSQC NMR spectrum of **4** in CDCl_3_. S64](#_Toc60637296)

[**Supplementary Fig. 22-6** HMBC NMR spectrum of **4** in CDCl_3_. S65](#_Toc60637297)

[**Supplementary Fig. 22-7** NOESY NMR spectrum of **4** in CDCl_3_. S66](#_Toc60637298)

[**Supplementary Fig. 22-8** HRMS spectrum of **4**. S67](#_Toc60637284)

[**Supplementary Fig. 23-1** ^1^H NMR spectrum of **5** in CDCl_3_. S68](#_Toc60637299)

[**Supplementary Fig. 23-2** ^13^C NMR spectrum of **5** in CDCl_3_. S69](#_Toc60637300)

[**Supplementary Fig. 23-3** DEPT135 and ^13^C NMR spectra of **5** in CDCl_3_. S70](#_Toc60637301)

[**Supplementary Fig. 23-4** ^1^H-^1^H COSY NMR spectrum of **5** in CDCl_3_. S71](#_Toc60637302)

[**Supplementary Fig. 23-5** HSQC NMR spectrum of **5** in CDCl_3_. S72](#_Toc60637303)

[**Supplementary Fig. 23-6** HMBC NMR spectrum of **5** in CDCl_3_. S73](#_Toc60637304)

[**Supplementary Fig. 23-7** NOESY NMR spectrum of **5** in CDCl_3_. S74](#_Toc60637305)

[**Supplementary Fig. 24-1** ^1^H NMR spectrum of **u1** in CDCl_3_. S75](#_Toc60637299)

[**Supplementary Fig. 24-2** ^13^C NMR spectrum of **u1** in CDCl_3_. S76](#_Toc60637300)

[**Supplementary Fig. 24-3** DEPT135 and ^13^C NMR spectra of **u1** in CDCl_3_. S77](#_Toc60637301)

[**Supplementary Fig. 24-4** ^1^H-^1^H COSY NMR spectrum of **u1** in CDCl_3_. S78](#_Toc60637302)

[**Supplementary Fig. 24-5** HSQC NMR spectrum of **u1**in CDCl_3_. S79](#_Toc60637303)

[**Supplementary Fig. 24-6** HMBC NMR spectrum of **u1** in CDCl_3._ S80](#_Toc60637304)

[Energies and Molecular Coordinates of Calculated Structures S81](#_Toc60637306)

Supplementary [References S87](#_Toc60637306)

**Supplementary Tables**

**Supplementary Table 1** | Strains and plasmids in this study.

| **Strain or plasmid** | **Characteristics** | **Reference or source** |
| --- | --- | --- |
| **Strains** |  |  |
| ***E. coli*** |  |  |
| TOP 10 | *recA1* | Invitrogen |
| BL21-(DE3) | *recA1* | Stratagene |
| ***P. citrunum*** |  |  |
| *P. citrinum* ATCC 9849 | Wild type *P. citrinum* used in this study | ATCC |
| △*ctdE* | *ctdE* inactivation mutant of *P. citrinum* ATCC 9849 | This study |
| △*ctdQ* | *ctdQ* inactivation mutant of *P. citrinum* ATCC 9849 | This study |
| △*ctdU* | *ctdU* inactivation mutant of *P. citrinum* ATCC 9849 | This study |
| **Plasmids** |  |  |
| *pET29* | Amp^r^, vector for protein expression | This study |
| *pET29-mbp* | Amp^r^, vector for protein fusion expression with MBP | This study |
| *pET29-phqK* | Amp^r^, vector for PhqK expression | This study |
| *pET29-ctdE* | Amp^r^, vector for CtdE expression | This study |
| *PUC57-Amp* | Amp^r^, vector for gene cloning | Addgene |
| *ctdUKO-P* | Amp^r^, gene inactivation plasmid used for △*ctdU* mutant construction | This study |
| *ctdEKO-P* | Amp^r^, gene inactivation plasmid used for △*ctdE* mutant construction | This study |
| *ctdQKO-P* | Amp^r^, gene inactivation plasmid used for △*ctdQ* mutant construction | This study |
| *pET29*-*ctdE* (D60A) | Amp^r^, vector for CtdE (D60A) expression | This study |
| *pET29*-*ctdE* (D60N) | Amp^r^, vector for CtdE (D60N) expression | This study |
| *pET29*-*ctdE* (I61A) | Amp^r^, vector for CtdE (I61A) expression | This study |
| *pET29*-*ctdE* (Y112A) | Amp^r^, vector for CtdE (Y112A) expression | This study |
| *pET29*-*ctdE* (V119A) | Amp^r^, vector for CtdE (V119A) expression | This study |
| *pET29*-*ctdE* (R122A) | Amp^r^, vector for CtdE (R122A) expression | This study |
| *pET29*-*ctdE* (R122K) | Amp^r^, vector for CtdE (R122K) expression | This study |
| *pET29*-*ctdE* (I198A) | Amp^r^, vector for CtdE (I198A) expression | This study |
| *pET29*-*ctdE* (R200A) | Amp^r^, vector for CtdE (R200A) expression | This study |
| *pET29*-*ctdE* (R200K) | Amp^r^, vector for CtdE (R200K) expression | This study |
| *pET29*-*ctdE* (L227A) | Amp^r^, vector for CtdE (L227A) expression | This study |
| *pET29*-*ctdE* (H229A) | Amp^r^, vector for CtdE (H229A) expression | This study |
| *pET29*-*ctdE* (L238A) | Amp^r^, vector for CtdE (L238A) expression | This study |
| *pET29*-mbp-*ctdE* (Y249A) | Amp^r^, vector for MBP-CtdE (Y249A) expression | This study |
| *pET29*-*ctdE* (M253A) | Amp^r^, vector for CtdE (M253A) expression | This study |
| *pET29*-*ctdE* (T333A) | Amp^r^, vector for CtdE (T333A) expression | This study |
| *pET29*-*ctdE* (I405A) | Amp^r^, vector for CtdE (I405A) expression | This study |

**Supplementary Table 2** | Primers used in this study.

| **Primer** | **Sequence** | **Description** |
| --- | --- | --- |
| ***ctdE-up-F*** | 5’-tcatgtcaacgctcctctctt-3’ | For colony PCR verification of △*ctdE* |
| ***ctdE-dn-R*** | 5’-cgcgaatagtgcttgaaaaa-3’ |  |
| ***ctdQ-up-F*** | 5’-gatggcgagctcacttaca-3’ | For colony PCR verification of △*ctdQ* |
| ***ctdQ-dn-R*** | 5’-actgtgcttcccccttgaa-3’ |  |
| ***ctdU-up-F*** | 5’-agttgaatcatgtggccaag-3’ | For colony PCR verification of △*ctdU* |
| ***ctdU-dn-R*** | 5’-ggagcaagtgatatggcatt-3’ |  |
| ***hyg-R*** | 5’-ctcggagggcgaagaatc-3’ | For colony PCR verification of *ctd* mutants |
| ***hyg-F*** | 5’-gcggtaccgtctgctgct-3’ |  |
| ***phqK-Exp-F*** | 5’-ctttaagaaggagatataccatgggtagcctgggcgaggaag-3’ | Plasmid construction for PhqK expression |
| ***phqK-Exp-R*** | 5’-ttagtgatggtggtggtgatgcgggcttttgttttgcagc-3’ |  |
| ***ctdE-Exp-F*** | 5’-ctttaagaaggagatataccatgaccaaaacaccagaagc-3’ | Plasmid construction for CtdE and CtdE mutants expression |
| ***ctdE-Exp-R*** | 5’-ttagtgatggtggtggtgatggaagtcccggcgctcactcac-3’ |  |
| ***ctdE(D60A)-up-R*** | 5’-gttaggaccaatgctgaagattgccccaatatgcatcatcttc-3’ | Plasmid construction for CtdE (D60A) expression |
| ***ctdE(D60A)-dn-F*** | 5’-gaagatgatgcatattggggcaatcttcagcattggtcctaac-3’ | Plasmid construction for CtdE (D60A) expression |
| ***ctdE(D60N)-up-R*** | 5’-tcggcgttaggaccaatgctgaagatattcccaatatgcatcatcttc-3’ | Plasmid construction for CtdE (D60N) expression |
| ***ctdE(D60N)-dn-F*** | 5’-gaagatgatgcatattgggaatatcttcagcattggtcctaacgccga-3’ | Plasmid construction for CtdE (D60N) expression |
| ***ctdE(I61A)-up-R*** | 5’-gcgttaggaccaatgctgaatgcatccccaatatgcatcatcttcg-3’ | Plasmid construction for CtdE (I61A) expression |
| ***ctdE(I61A)-dn-F*** | 5’-gatgatgcatattggggatgcattcagcattggtcctaacgcc-3’ | Plasmid construction for CtdE (I61A) expression |
| ***ctdE(Y112A)-up-R*** | 5’-gtatccttctccttcacgtgccccctccatggtattgacgttc-3’ | Plasmid construction for CtdE (Y112A) expression |
| ***ctdE(Y112)-dn-F*** | 5’-acgtcaataccatggagggggcacgtgaaggagaaggatacgtg-3’ | Plasmid construction for CtdE (Y112A) expression |
| ***ctdE(V119A)-up-R*** | 5’-cactgcttctgcgcggttgattgcgtatccttctccttcacggtac-3’ | Plasmid construction for CtdE (V119A) expression |
| ***ctdE(V119A)-dn-F*** | 5’-gtgaaggagaaggatacgcaatcaaccgcgcagaagcagt-3’ | Plasmid construction for CtdE (V119A) expression |
| ***CtdE(R122A)-up-R*** | 5’-gaagaaaatatccactgcttctgctgcgttgatcacgtatccttctcc-3’ | Plasmid construction for CtdE (R122A) expression |
| ***CtdE(R122A)-dn-F*** | 5’-gaagcagtggatattttcttcgaatacgcgcagagtttgggt-3’ | Plasmid construction for CtdE (R122A) expression |
| ***CtdE(R122K)-up-R*** | 5’-gaagaaaatatccactgcttctgctttgttgatcacgtatccttctcc-3’ | Plasmid construction for CtdE (R122K) expression |
| ***CtdE(R122K)-dn-F*** | 5’-gaagcagtggatattttcttcgaatacgcgcagagtttgggt-3’ | Plasmid construction for CtdE (R122K) expression |
| ***CtdE(R122N)-up-R*** | 5’-gaagaaaatatccactgcttctgcgttgttgatcacgtatccttctcc-3’ | Plasmid construction for CtdE (R122N) expression |
| ***CtdE(R122N)-dn-F*** | 5’-gaagcagtggatattttcttcgaatacgcgcagagtttgggt-3’ | Plasmid construction for CtdE (R122N) expression |
| ***CtdE(R122L)-up-R*** | 5’-gaagaaaatatccactgcttctgctaggttgatcacgtatccttctcc-3’ | Plasmid construction for CtdE (R122L) expression |
| ***CtdE(R122L)-dn-F*** | 5’-gaagcagtggatattttcttcgaatacgcgcagagtttgggt-3’ | Plasmid construction for CtdE (R122L) expression |
| ***CtdE(R122E)-up-R*** | 5’-gaagaaaatatccactgcttctgcttcgttgatcacgtatccttctcc-3’ | Plasmid construction for CtdE (R122E) expression |
| ***CtdE(R122E)-dn-F*** | 5’-gaagcagtggatattttcttcgaatacgcgcagagtttgggt-3’ | Plasmid construction for CtdE (R122E) expression |
| ***CtdE(I198A)-up-R*** | 5’-atggcataccccgatcgatatgctgcacttccggtcttcttg-3’ | Plasmid construction for CtdE (I198A) expression |
| ***CtdE(I198A)-dn-F*** | 5’-caagaagaccggaagtgcagcatatcgatcggggtatgccatg-3’ | Plasmid construction for CtdE (I198A) expression |
| ***CtdE(R200A)-up-R*** | 5’-gctcctccatggcataccccgatgcatagattgcacttccggtc-3’ | Plasmid construction for CtdE (R200A) expression |
| ***CtdE(R200A)-dn-F*** | 5’-gaccggaagtgcaatctatgcatcggggtatgccatggaggagc-3’ | Plasmid construction for CtdE (R200A) expression |
| ***CtdE(R200K)-up-R*** | 5’-gctcctccatggcataccccgatttatagattgcacttccggtc-3’ | Plasmid construction for CtdE (R200K) expression |
| ***CtdE(R200K)-dn-F*** | 5’-gaccggaagtgcaatctataaatcggggtatgccatggaggag-3’ | Plasmid construction for CtdE (R200K) expression |
| ***CtdE(L227A)-up-R*** | 5’-ttgccgataaagtggtatgcttggtctacgtcttcttttccttc-3’ | Plasmid construction for CtdE (L227A) expression |
| ***CtdE(L227A)-dn-F*** | 5’-ggaaaagaagacgtagaccaagcataccactttatcggcaaggacattactg-3’ | Plasmid construction for CtdE (L227A) expression |
| ***CtdE(H229A)-up-R*** | 5’-tgtccttgccgataaatgcgtatagttggtctacgtcttc-3’ | Plasmid construction for CtdE (H229A) expression |
| ***CtdE(H229A)-dn-F*** | 5’-gaagacgtagaccaactatacgcatttatcggcaaggacattac-3’ | Plasmid construction for CtdE (H229A) expression |
| ***CtdE(L238A)-up-R*** | 5’-tccccttcggccagttcccactgctacagtaatgtccttgccgataaag-3’ | Plasmid construction for CtdE (L238A) expression |
| ***CtdE(L238A)-dn-F*** | 5’-ggacattactgtagcagtgggaactggccgaag-3’ | Plasmid construction for CtdE (L238A) expression |
| ***MBP-CtdE(Y249A)-up-F*** | 5’-gacaaggaaaacctgtattttcagggcatgaccaaaacaccagaagctcc-3’ | Plasmid construction for MBP-CtdE (Y249A) expression |
| ***MBP-CtdE(Y249A)-up-R*** | 5’-ctcttgtgcatgcatccccatgcaacatcctttccccttcg-3’ | Plasmid construction for MBP-CtdE (Y249A) expression |
| ***MBP-CtdE(Y249A)-dn-F*** | 5’-cgaaggggaaaggatgttgcatggggatgcatgcacaagagcct-3’ | Plasmid construction for MBP-CtdE (Y249A) expression |
| ***MBP-CtdE(Y249A)-dn-R*** | 5’-gatctcagtggtggtggtggtggtgctcgaggaagtcccggcgctcactca-3’ | Plasmid construction for MBP-CtdE (Y249A) expression |
| ***CtdE(M253A)-up-R*** | 5’-catcgtgaaggctcttgtgtgcgcatccccagtaaacatcct-3’ | Plasmid construction for CtdE (M253A) expression |
| ***CtdE(M253A)-dn-F*** | 5’-aggatgtttactggggatgcgcacacaagagccttcacgatg-3’ | Plasmid construction for CtdE (M253A) expression |
| ***CtdE(T333A)-up-R*** | 5’-cttggtttgctccttgacctgcattggggagaaaaggatgagcg-3’ | Plasmid construction for CtdE (T333A) expression |
| ***CtdE(T333A)-dn-F*** | 5’-ctcatccttttctccccaatgcaggtcaaggagcaaaccaagcaattg-3’ | Plasmid construction for CtdE (T333A) expression |
| ***CtdE(I405A)-up-R*** | 5’-gcgcgttgccattgttggagcgtcttcgtcc-3’ | Plasmid construction for CtdE (I405A) expression |
| ***CtdE(I405A)-dn-F*** | 5’-gatggtgaaaaggacgaagacgctccaacaatggcaacgcgcccagcctggatttattc-3’ | Plasmid construction for CtdE (I405A) expression |

**Supplementary Table 3** | BLASTP CtdE homologs in NCBI databases.

| **Description** | **Organism** | **Total Score** | **Query Cover** | **E value** | **Per. Ident** | **Accession** |
| --- | --- | --- | --- | --- | --- | --- |
| **NotI**: FAD-dependent monooxygenase | *Aspergillus protuberus* | 458 | 89% | 6e^-86^ | 45.24% | AGC83580.1 |
| **NotB**: FAD-dependent monooxygenase | *Aspergillus protuberus* | 184 | 94% | 6e^-60^ | 37.75% | AGC83573.1 |
| **PhqK**: FAD monooxygenase | *Penicillium fellutanum* | 180 | 92% | 2e^-47^ | 34.30% | AGA37278.1 |
| Asperlicin C monooxygenase | *Aspergillus alliaceus* | 176 | 92% | 2e^-51^ | 41.84% | [P0DOW1.1](https://www.ncbi.nlm.nih.gov/protein/P0DOW1.1?report=genbank&log$=prottop&blast_rank=2&RID=7ZMV35WS01R) |
| **RoqM:** FAD-dependent oxidoreductase | *Penicillium rubens* wisconsin 54-1255 | 177 | 91% | 7e^-52^ | 39.24% | [B6HJU4.1](https://www.ncbi.nlm.nih.gov/protein/B6HJU4.1?report=genbank&log$=prottop&blast_rank=1&RID=7ZMV35WS01R) |
| **NodY1:** FAD-dependent oxidoreductase | *Hypoxylon pulicicidum* | 173 | 91% | 2e^-50^ | 36.93% | [A0A2I6PJ01.1](https://www.ncbi.nlm.nih.gov/protein/A0A2I6PJ01.1?report=genbank&log$=prottop&blast_rank=3&RID=7ZMV35WS01R) |
| **TqaH:** FAD-dependent oxidoreductase | *Penicillium aethiopicum* | 171 | 91% | 3e^-54^ | 41.00% | ADY16696.1 |
| **NodY2:** FAD-dependent oxidoreductase | *Hypoxylon pulicicidum* | 168 | 93% | 2e^-48^ | 36.82% | [A0A2I6PIZ8.1](https://www.ncbi.nlm.nih.gov/protein/A0A2I6PIZ8.1?report=genbank&log$=prottop&blast_rank=4&RID=7ZMV35WS01R) |
| **FqzB**: FAD-dependent monooxygenase | *Aspergillus fumigatus* Af293 | 119 | 76% | 3e^-35^ | 35.00% | Q4WLW7.1 |
| **Fsr3**: FAD-dependent monooxygenase | *Fusarium fujikuroi* IMI 58289 | 95.9 | 72% | 2e^-21^ | 31.66% | [S0DQN6.1](https://www.ncbi.nlm.nih.gov/protein/S0DQN6.1?report=genbank&log$=prottop&blast_rank=6&RID=7ZMV35WS01R) |

Attached figure from **Supplementary Table 4**

**Supplementary Table 4** | NMR data of **1**^a^ and 21*R*-citrinadin A in reference^1^ (*δ* in ppm, *J* in Hz).

|  | 21*R*-Citrinadin A in Ref. | | |  | **1** | | | | | | |
| --- | --- | --- | --- | --- | --- | --- | --- | --- | --- | --- | --- |
| **Position** | **^1^H** | **^13^C** |  |  |  | **^1^H** | **^13^C** |  | **^1^H-^1^H COSY** | **HMBC** | **ROESY** |
| **1** | 9.55 s |  | NH |  |  | 9.56 s |  | NH |  | C-3, 3a |  |
| **2** |  | 185.8 | C |  |  |  | 185.9 | C |  |  |  |
| **3** |  | 60.9 | C |  |  |  | 61.0 | C |  |  |  |
| **3a** |  | 136.1 | C |  |  |  | 136.3 | C |  |  |  |
| **4** | 7.67 d (7.2) | 133.3 | CH |  |  | 7.69 d (7.8) | 133.4 | CH | H-5 | C-3, 6, 7a | H-26, 29 |
| **5** | 7.13 dd (7.2, 7.2) | 121.9 | CH |  |  | 7.15 dd (7.8, 7.8) | 122.0 | CH | H-4, 6 | C-3a, 7 |  |
| **6** | 7.70 d (8.4, 7.2) | 127.0 | CH |  |  | 7.72 d (7.8) | 127.1 | CH | H-5 | C-4, 7a |  |
| **7** |  | 117.2 | C |  |  |  | 117.3 | C |  |  |  |
| **7a** |  | 143.0 | C |  |  |  | 143.2 | C |  |  |  |
| **8** | 2.09, | 40.9 | CH_2_ |  | a | 2.11 d (14.2) | 41.0 | CH_2_ |  | C-2, 3a, 18 |  |
|  | 2.05, (Abq, 13.8) |  |  |  | b | 2.07d (14.2) |  |  |  | C-2, 3a, 10, 19 |  |
| **9** |  | 68.7 | C |  |  |  | 68.8 | C |  |  |  |
| **10** | 3.18 d (10.8) | 50.1 | CH_2_ |  | α | 3.17d (10.8) | 50.3 | CH_2_ |  | C-12, 16, 18 | H-17α, 27, 18-OH |
|  | 2.58 d (10.8) |  |  |  | β | 2.57 d (10.8) |  |  |  | C-12, 16, 18 | H-12 |
| **11** |  |  | N |  |  |  |  | N |  |  |  |
| **12** | 3.03 quin | 54.0 | CH |  |  | 3.03 m | 54.1 | CH | H-13, 27 | C-14, 16 | H-10β |
| **13** | 2.07-2.01 | 36.1 | CH_2_ |  | a | 2.04 | 36.3 | CH_2_ | H-12, 14 |  |  |
|  | 1.83-1.79 |  |  |  | b | 1.80 |  |  | H-12, 14 | C-15 |  |
| **14** | 5.23-5.22 m | 67.9 | CH |  |  | 5.22 m | 68.1 | CH | H-13, 15 | C-12, 16 |  |
| **15** | 1.83-1.79 | 39.0 | CH_2_ |  | a | 1.80 | 39.2 | CH_2_ | H-14, 16 | C-13 |  |
|  | 1.52 |  |  |  | b | 1.50 m |  |  | H-14, 16 |  |  |
| **16** | 3.22-3.16 m | 42.2 | CH |  |  | 3.18 m | 42.3 | CH | H-15, 17 |  | H-17α |
| **17** | 1.56 ddd (14.7, 11.4, 3.6) | 35.0 | CH_2_ |  | α | 1.56 dd (13.2, 3.6) | 35.3 | CH_2_ | H-16 | C-9 | H-10α, 16 |
|  | 1.39-1.32 |  |  |  | β | 1.35 m |  |  | H-16 |  |  |
| **18** |  | 83.8 | C |  |  |  | 84.0 | C |  |  |  |
| **18-OH** | 4.55 d (3.0) |  | OH |  |  | 4.54 d (2.5) |  | OH |  | C-17, 19 |  |
| **19** |  | 52.0 | C |  |  |  | 52.2 | C |  |  |  |
| **20** |  | 194.8 | C |  |  |  | 194.9 | C |  |  |  |
| **21** | 4.05 s | 64.2 | CH |  |  | 4.05 s | 64.4 | CH |  | C-24 |  |
| **22** |  | 61.5 | C |  |  |  | 61.6 | C |  |  |  |
| **23** | 1.58 s | 24.3 | CH_3_ |  |  | 1.60 s | 24.5 | CH_3_ |  | C-21, 24 |  |
| **24** | 1.25 s | 18.6 | CH_3_ |  |  | 1.25 s | 18.7 | CH_3_ |  | C-21, 23 |  |
| **26** | 2.13 s | 29.7 | CH_3_ |  |  | 2.30 s | 29.9 | CH_3_ |  | C-9 | H-4 |
| **27** | 1.22 d (7.2) | 13.5 | CH_3_ |  |  | 1.21 d (6.9) | 13.7 | CH_3_ | H-12 | C-13 | H-10α, 17α |
| **28** | 0.99 s | 22.1 | CH_3_ |  |  | 0.99 s | 22.3 | CH_3_ |  | C-3, 18. 29 | H-17α, 18-OH |
| **29** | 1.39-1.32 | 28.3 | CH_3_ |  |  | 1.35 s | 28.4 | CH_3_ |  | C-3, 18. 28 | H-4 |
| **1’** |  | 171.0 | C |  |  |  | 171.1 | C |  |  |  |
| **2’** | 2.70 d (10.2) | 74.6 | CH |  |  | 2.70 d (10.5) | 74.8 | CH | H-4’ | C-5’, 6’ |  |
| **4’** | 2.07-2.01 | 27.5 | CH |  |  | 2.04 | 27.6 | CH | H-2’, 5’, 6’ |  |  |
| **5’** | 0.90 d (6.6) | 19.3 | CH_3_ |  |  | 0.90 d (6.6) | 19.4 | CH_3_ | H-4’ | C-2’, 6’ |  |
| **6’** | 0.98 d (6.6) | 20.0 | CH_3_ |  |  | 0.98 d (6.6) | 20.1 | CH_3_ | H-4’ | C-2’, 4’ |  |
| **7’** | 2.31 s | 41.6 | CH_3_ |  |  | 2.31 s | 41.7 | CH_3_ |  | C-2’ |  |
| **8’** | 2.31 s | 41.6 | CH_3_ |  |  | 2.31 s | 41.7 | CH_3_ |  | C-2’ |  |

^a^Measured in CDCl_3_, 600 MHz for ^1^H and 150 MHz for ^13^C NMR. Overlapped signals are reported without designating multiplicity.

|  |  |  |
| --- | --- | --- |

**Supplementary Table 5** | NMR data of compound **2** (*δ* in ppm, *J* in Hz)^a^.

| **Position** |  | **^1^H** | **^13^C** |  | **^1^H-^1^H COSY** | **HMBC** | **NOESY** |
| --- | --- | --- | --- | --- | --- | --- | --- |
| **1** |  | 7.93 s |  | NH |  | C-2, 3, 3a, 7a | H-7, 28, 29 |
| **2** |  |  | 141.5 | C |  |  |  |
| **3** |  |  | 103.4 | C |  |  |  |
| **3a** |  |  | 127.1 | C |  |  |  |
| **4** |  | 7.43 d (7.7) | 118.0 | CH | H-5 | C-3, 6, 7a | H-8α |
| **5** |  | 7.11 dd (7.7, 7.7) | 119.7 | CH | H-4, 6 | C-3a, 7 |  |
| **6** |  | 7.17 dd (7.7, 7.7) | 122.0 | CH | H-5, 7 | C-4, 7a |  |
| **7** |  | 7.33 d (7.7) | 111.0 | CH | H-6 | C-3a, 5 | H-1 |
| **7a** |  |  | 136.4 | C |  |  |  |
| **8** | β | 2.89 d (16.9) | 28.5 | CH_2_ |  | C-2, 3a, 10 | H-10β, 18 |
|  | α | 2.63 d (16.9) |  |  |  | C-2, 3a, 18 | H-4, 25 |
| **9** |  |  | 54.7 | C |  |  |  |
| **10** | β | 3.16 d (10.8) | 62.1 | CH_2_ |  | C-16, 18 | H-8β |
|  | α | 2.63 d (10.8) |  |  |  | C-8, 12, 16, 18 | H-12 |
| **12** |  | 2.34 m | 57.5 | CH | H-13, 27 |  | H-10α |
| **13** | a | 1.64 m | 34.1 | CH_2_ | H-12, 14 | C-15 |  |
|  | b | 1.22 m |  |  | H-12, 14 | C-27 |  |
| **14** | a | 1.93 m | 20.9 | CH_2_ | H-13, 15 | C-12 |  |
|  | b | 1.60 m |  |  | H-13, 15 | C-16 |  |
| **15** | a | 2.19 m | 29.2 | CH_2_ | H-14 | C-13 |  |
|  | b | 1.31 dt (13.2, 4.3) |  |  | H-14 | C-17, 30 |  |
| **16** |  |  | 58.8 | C |  |  |  |
| **17** | β | 2.13 dd (13.7, 10.4) | 35.1 | CH_2_ | H-18 | C-15, 19, 30 |  |
|  | α | 1.74 dd (13.7, 3.3) |  |  | H-18 | C-15, 19, 30 |  |
| **18** |  | 2.06 dd (10.4, 3.3) | 45.8 | CH | H-17 | C-8, 10, 16, 28, 29 | H-8β, 28 |
| **19** |  |  | 31.4 | C |  |  |  |
| **25** |  | 5.61 s |  | NH |  | C-8, 16 | H-8α, 29 |
| **27** |  | 1.10 d (6.0) | 21.3 | CH_3_ | H-12 | C-13 |  |
| **28** |  | 1.26 s | 24.9 | CH_3_ |  | C-2, 18, 29 | H-1 |
| **29** |  | 1.19 s | 21.3_3_ | CH_3_ |  | C-2, 18, 28 | H-1 |
| **30** |  |  | 174.2 | C |  |  |  |

^a^Measured in CDCl_3_, 600 MHz for ^1^H and 150 MHz for ^13^C NMR. Overlapped signals are reported without designating multiplicity.

|  |  |
| --- | --- |

**Supplementary Table 6** | NMR data of compound **3** (*δ* in ppm, *J* in Hz)^a^.

| **Position** |  | **^1^H** | **^13^C** |  | **^1^H-^1^H COSY** | **HMBC** | **NOESY** |
| --- | --- | --- | --- | --- | --- | --- | --- |
| 1 |  | 7.87 s |  | NH |  | C-2, 3, 3a, 7a | H-7, 28, 29 |
| 2 |  |  | 141.1 | C |  |  |  |
| 3 |  |  | 103.7 | C |  |  |  |
| 3a |  |  | 127.1 | C |  |  |  |
| 4 |  | 7.29 d (7.7) | 116.0 | CH | H-5 | C-3, 6, 7a | H-8*α* |
| 5 |  | 7.05 dd (7.7, 7.7) | 120.0 | CH | H-4, 6 | C-3a, 7 |  |
| 6 |  | 6.98 d (7.7) | 121.7 | CH | H-5, 7 | C-4, 7a |  |
| 7 |  |  | 123.7 | C |  |  |  |
| 7a |  |  | 135.6 | C |  |  |  |
| 8 | β | 2.88 d (17.0) | 29.3 | CH_2_ |  | C-2, 3a, 10 | H-10β, 18 |
|  | α | 2.77 d (17.0) |  |  |  | C-2, 3a, 18 | H-4, 25 |
| 9 |  |  | 54.8 | C |  |  |  |
| 10 | β | 3.15 d (10.8) | 62.1 | CH_2_ |  | C-16, 18 | H-8β |
|  | α | 2.62 d (10.8) |  |  |  | C-8, 12, 16, 18 | H-12 |
| 12 |  | 2.33 m | 57.5 | CH | H-13, 27 |  | H-10α |
| 13 | a | 1.65 m | 35.1 | CH_2_ | H-12, 14 | C-15 |  |
|  | b | 1.22 m |  |  | H-12, 14 | C-27 |  |
| 14 | a | 1.93 m | 20.9 | CH_2_ | H-13, 15 | C-12 |  |
|  | b | 1.60 m |  |  | H-13, 15 | C-16 |  |
| 15 | a | 2.13 dd (13.8, 10.4) | 31.5 | CH_2_ | H-14 | C-13 |  |
|  | b | 1.31 dd (10.4, 4.3) |  |  | H-14 | C-17, 30 |  |
| 16 |  |  | 58.8 | C |  |  |  |
| 17 | β | 2.13 dd (13.8, 10.4) | 35.9 | CH_2_ | H-18 | C-15, 19, 30 |  |
|  | α | 1.74 dd (13.8, 3.4) |  |  | H-18 | C-15, 19, 30 | H-29 |
| 18 |  | 2.06 dd (10.4, 3.4) | 45.8 | CH | H-17 | C-8, 10, 16, 28, 29 | H-8β, 28 |
| 19 |  |  | 34.1 | C |  |  |  |
| 20 |  | 3.56 m | 31.0 | CH_2_ | H-21 | C-6, 7a, 22 |  |
| 21 |  | 5.41 t (7.3) | 122.6 | CH | H-20 | C-23, 24 | H-23 |
| 22 |  |  | 133.2 | C |  |  |  |
| 23 |  | 1.78 s | 25.8 | CH_3_ |  | C-22, 24 | H-21 |
| 24 |  | 1.85 s | 18.1 | CH_3_ |  | C-22, 23 |  |
| 25 |  | 5.60 s |  | NH |  | C-8, 16 | H-8*α*, 29 |
| 27 |  | 1.10 d (6.0) | 21.4 | CH_3_ | H-12 | C-13 |  |
| 28 |  | 1.24 s | 28.5 | CH_3_ |  | C-2, 18, 29 | H-1, 18 |
| 29 |  | 1.18 s | 24.9 | CH_3_ |  | C-2, 18, 28 | H-1, 17*α*, 25 |
| 30 |  |  | 174.2 | C |  | C-8, 16 | H-8*α*, 29 |

^a^Measured in CDCl_3_, 600 MHz for ^1^H and 150 MHz for ^13^C NMR. Overlapped signals are reported without designating multiplicity.

**Supplementary Table 7** | NMR data of compound **4** (*δ* in ppm, *J* in Hz)^a^.

| **Position** |  | **^1^H** | **^13^C** |  | **^1^H-^1^H COSY** | **HMBC** | **NOESY** |
| --- | --- | --- | --- | --- | --- | --- | --- |
| **1** |  | 7.37 s |  | NH |  | C-3, 3a, 7a |  |
| **2** |  |  | 183.0 | C |  |  |  |
| **3** |  |  | 62.8 | C |  |  |  |
| **3a** |  |  | 130.1 | C |  |  |  |
| **4** |  | 7.31 d (7.6) | 126.4 | CH | H-5 | C-3, 6, 7a | H-8β, 29 |
| **5** |  | 7.22 dd (7.6, 7.6) | 122.0 | CH | H-4, 6 | C-3a, 7 |  |
| **6** |  | 7.03 dd (7.6, 7.6) | 128.4 | CH | H-5, 7 | C-4, 7a |  |
| **7** |  | 6.84 d (7.6) | 109.4 | CH | H-6 | C-3a, 5 |  |
| **7a** |  |  | 141.2 | C |  |  |  |
| **8** | α | 2.44 d (15.4) | 41.6 | CH_2_ |  | C-2, 10, 19 | H-10α |
|  | β | 2.06 d (15.4) |  |  |  | C-2, 3a, 18 | H-4, 25, 29 |
| **9** |  |  | 61.8 | C |  |  |  |
| **10** | α | 3.62 d (9.0) | 65.0 | CH_2_ |  | C-12, 16, 18 | H-8α, 18 |
|  | β | 2.41 d (9.0) |  |  |  | C-12, 16, 18 | H-12 |
| **12** |  | 2.20 m | 59.3 | CH | H-13, 27 |  | H-10β |
| **13** | a | 1.63 | 34.7 | CH_2_ | H-12, 14 | C-15 |  |
|  | b | 1.25 m |  |  | H-12, 14 |  |  |
| **14** | a | 1.83 m | 21.1 | CH_2_ | H-13, 15 |  |  |
|  | b | 1.61 |  |  | H-13, 15 |  |  |
| **15** | a | 2.29 m | 32.2 | CH_2_ | H-14 | C-13 |  |
|  | b | 1.38 td (13.5, 3.9) |  |  | H-14 | C-30 |  |
| **16** |  |  | 59.7 | C |  |  |  |
| **17** | α | 1.89 dd (13.3, 9.1) | 35.6 | CH_2_ | H-18 | C-9, 15, 30 | H-28 |
|  | β | 1.55 m |  |  | H-18 | C-19, 30 | H-29 |
| **18** |  | 3.12 dd (9.1, 9.1) | 46.8 | CH | H-17 | C-3, 10, 28, 29 | H-10α, 28 |
| **19** |  |  | 47.7 | C |  |  |  |
| **25** |  | 6.71 s |  | NH |  |  | H-8β, 29 |
| **27** |  | 1.08 d (6.0) | 21.7 | CH_3_ | H-12 | C-13 |  |
| **28** |  | 0.77 s | 23.1 | CH_3_ |  | C-3, 18, 29 | H-17α, 18 |
| **29** |  | 0.99 s | 20.9 | CH_3_ |  | C-3, 18, 28 | H-4, 8β, 17β, 25 |
| **30** |  |  | 174.5 | C |  |  |  |

^a^Measured in CDCl_3_, 600 MHz for ^1^H and 150 MHz for ^13^C NMR. Overlapped signals are reported without designating multiplicity.

**Supplementary Table 8** | NMR data of compound **5** (*δ* in ppm, *J* in Hz)^a^.

| **Position** |  | **^1^H** | **^13^C** |  | **^1^H-^1^H COSY** | **HMBC** | **NOESY** |
| --- | --- | --- | --- | --- | --- | --- | --- |
| **1(NH)** |  | 7.49 s |  |  |  | C-3, 3a, 7a |  |
| **2** |  |  | 183.2 | C |  |  |  |
| **3** |  |  | 62.9 | C |  |  |  |
| **3a** |  |  | 130.1 | C |  |  |  |
| **4** |  | 7.22 d (7.6) | 124.1 | CH | H-5 | C-3, 6, 7a | H-8*β*, 29 |
| **5** |  | 7.03 dd (7.6, 7.6) | 121.9 | CH | H-4, 6 | C-3a, 7 |  |
| **6** |  | 6.96 d (7.6) | 128.8 | CH | H-5 | C-4, 7a |  |
| **7** |  |  | 122.4 | C |  |  |  |
| **7a** |  |  | 139.8 | C |  |  |  |
| **8** | α | 2.42 d (15.4) | 41.6 | CH_2_ |  | C-2, 10, 19 |  |
|  | β | 2.06 d (15.4) |  |  |  | C-2, 3a, 10, 18 | H-4, 25, 29 |
| **9** |  |  | 61.8 | C |  |  |  |
| **10** | α | 3.60 d (9.0) | 65.0 | CH_2_ |  | C-16, 18 | H-17α, 18 |
|  | β | 2.41 d (9.0) |  |  |  | C-12, 16, 18 | H-12 |
| **12** |  | 2.20 m | 59.3 | CH | H-13, 27 | C-10 | H-10β |
| **13** | a | 1.60 | 34.7 | CH_2_ | H-12, 14 | C-15 |  |
|  | b | 1.24 m |  |  | H-12, 14 | C-27 |  |
| **14** | a | 1.84 m | 21.1 | CH_2_ | H-13, 15 |  |  |
|  | b | 1.60 overlapped |  |  | H-13, 15 | C-15 |  |
| **15** | a | 2.30 m | 32.2 | CH_2_ | H-14 | C-13 |  |
|  | b | 1.38 td (12.3, 3.9) |  |  | H-14 | C-17, 30 |  |
| **16** |  |  | 59.7 | C |  |  |  |
| **17** | α | 1.89 dd (13.3, 9.1) | 35.7 | CH_2_ | H-18 | C-9, 15, 30 | H-18 |
|  | β | 1.54 dd (13.3, 9.1) |  |  | H-18 | C-19, 30 | H-29 |
| **18** |  | 3.12 dd (9.1) | 46.9 | CH | H-17 | C-3, 10, 28, 29 | H-10α, 17α, 28 |
| **19** |  |  | 47.6 | C |  |  |  |
| **20** | a | 3.31 dd (16.2, 7.5) | 30.4 | CH_2_ | H-21 | C-6, 7a, 22 |  |
|  | b | 3.25 dd (16.2, 6.4) |  |  | H-21 | C-6, 7a, 22 |  |
| **21** |  | 5.21 dd (6.4, 6.4) | 121.1 | CH | H-20 | C-23, 24 |  |
| **22** |  |  | 134.6 | C |  |  |  |
| **23** |  | 1.78 s | 25.9 | CH_3_ |  | C-21, 24 |  |
| **24** |  | 1.85 s | 18.2 | CH_3_ |  | C-21, 23 |  |
| **25 (NH)** |  | 5.60 s |  |  |  |  | H-8β, 29 |
| **27** |  | 1.08 d (6.0) | 21.6 | CH_3_ | H-12 | C-13 |  |
| **28** |  | 0.77 s | 23.2 | CH_3_ |  | C-3, 18, 29 | H-17α, 18 |
| **29** |  | 0.96 s | 20.9 | CH_3_ |  | C-3, 18, 28 | H-4, 8β, 17β, 25 |
| **30** |  |  | 174.6 | C |  |  |  |

^a^Measured in CDCl_3_, 600 MHz for ^1^H and 150 MHz for ^13^C NMR. Overlapped signals are reported without designating multiplicity.

**Supplementary Table 9** | NMR data of compound **u1** (*δ* in ppm, *J* in Hz)^a^.

| **Position** |  | **^1^H** | **^13^C** |  | **^1^H-^1^H COSY** | **HMBC** |
| --- | --- | --- | --- | --- | --- | --- |
| **1** |  | 7.41 s |  | NH |  | C-3, 3a |
| **2** |  |  | 185.9 | C |  |  |
| **3** |  |  | 62.3 | C |  |  |
| **3a** |  |  | 134.1 | C |  |  |
| **4** |  | 7.45 d (7.4) | 128.4 | CH | H-5 | C-3, 6, 7a |
| **5** |  | 7.06 dd (7.4, 7.4) | 122.7 | CH | H-4, 6 | C-3a, 7 |
| **6** |  | 7.18, dd (7.4, 7.4) | 127.5 | CH | H-5, 7 | C-4, 7a |
| **7** |  | 6.85, d (7.4) | 109.4 | CH | H-6 | C-3a, 6 |
| **7a** |  |  | 140.5 | C |  |  |
| **8** | α | 2.08 | 41.0 | CH_2_ |  | C-2, 3a, 18, 19 |
|  | β | 2.07 |  |  |  | C-2, 3a, 10 |
| **9** |  |  | 68.6 | C |  |  |
| **10** | α | 3.09 | 50.6 | CH_2_ |  |  |
|  | β | 2.57 |  |  |  | C-16 |
| **12** |  | 3.05 | 55.5 | CH | H-13, 27 |  |
| **13** | a | 1.81 m | 32.6 | CH_2_ | H-12, 14 |  |
|  | b | 1.52 m |  |  | H-12, 14 |  |
| **14** |  | 1.58 | 19.0 | CH_2_ | H-13, 15 |  |
|  |  | 1.50 |  |  |  |  |
| **15** | a | 1.51 | 35.2 | CH_2_ | H-14, 16 |  |
|  | b | 1.46 |  |  | H-14, 16 |  |
| **16** |  | 2.93 m | 47.4 | CH | H-15, 17 |  |
| **17** | α | 1.61 | 34.5 | CH_2_ | H-16 |  |
|  | β | 1.22 |  |  | H-16 |  |
| **18** |  |  | 84.1 | C |  |  |
| **18-OH** |  | 4.78 br s |  | OH |  |  |
| **19** |  |  | 51.7 | C |  |  |
| **26** |  | 2.93 s | 29.9 | CH_3_ |  | C-9 |
| **27** |  | 1.08 s | 10.7 | CH_3_ | H-12 | C-13 |
| **28** |  | 0.98 s | 22.2 | CH_3_ |  | C-3, 18. 29 |
| **29** |  | 1.37 s | 28.2 | CH_3_ |  | C-3, 18. 28 |

^a^Measured in CDCl_3_, 600 MHz for ^1^H and 150 MHz for ^13^C NMR, Overlapped signals are reported without designating multiplicity

**Supplementary Table 10** | Crystal data and structure refinement for compound **2**.

| Empirical formula | C_25_H_34_N_4_O_2_ |
| --- | --- |
| Formula weight | 422.56 |
| Temperature/K | 100.00(10) |
| Crystal system | orthorhombic |
| Space group | P2_1_2_1_2_1_ |
| a/Å | 7.26818(5) |
| b/Å | 16.00320(9) |
| c/Å | 19.51558(12) |
| α/° | 90 |
| β/° | 90 |
| **γ/°** | 90 |
| Volume/Å^3^ | 2269.94(2) |
| Z | 4 |
| ρ_calc_g/cm^3^ | 1.236 |
| μ/mm^‑1^ | 0.629 |
| F (000) | 912.0 |
| Crystal size/mm^3^ | 0.066 × 0.021 × 0.0126 |
| Radiation | Cu Kα (λ = 1.54184) |
| 2θ range for data collection/° | 7.144 to 150.368 |
| Index ranges | -9 ≤ h ≤ 8, -20 ≤ k ≤ 19, -23 ≤ l ≤ 24 |
| Reflections collected | 59777 |
| Independent reflections | 4590 [R_int_ = 0.0219, R_sigma_ = 0.0073] |
| Data/restraints/parameters | 4590/3/417 |
| Goodness-of-fit on F^2^ | 1.030 |
| Final R indexes [I>=2σ (I)] | R_1_ = 0.0236, wR_2_ = 0.0609 |
| Final R indexes [all data] | R_1_ = 0.0238, wR_2_ = 0.0611 |
| Largest diff. peak/hole / e Å^-3^ | 0.20/-0.13 |
| Flack parameter | -0.01(3) |

**Supplementary Table 11** | Crystal data and structure refinement for compound **4**.

| Empirical formula | C_23_H_29_N_3_O_2_ |
| --- | --- |
| Formula weight | 379.49 |
| Temperature/K | 100.00(10) |
| Crystal system | monoclinic |
| Space group | P2_1_ |
| a/Å | 11.35384(17) |
| b/Å | 7.16146(13) |
| c/Å | 12.3380(2) |
| α/° | 90 |
| β/° | 98.2149(15) |
| γ/° | 90 |
| Volume/Å^3^ | 992.91(3) |
| Z | 2 |
| ρ_calc_g/cm^3^ | 1.269 |
| μ/mm^‑1^ | 0.647 |
| F(000) | 408.0 |
| Crystal size/mm^3^ | 0.232 × 0.047 × 0.03 |
| Radiation | Cu Kα (λ = 1.54184) |
| 2θ range for data collection/° | 7.24 to 150.072 |
| Index ranges | -14 ≤ h ≤ 14, -8 ≤ k ≤ 8, -15 ≤ l ≤ 15 |
| Reflections collected | 18373 |
| Independent reflections | 3837 [R_int_ = 0.0296, R_sigma_ = 0.0203] |
| Data/restraints/parameters | 3837/1/369 |
| Goodness-of-fit on F^2^ | 1.058 |
| Final R indexes [I>=2σ (I)] | R_1_ = 0.0273, wR_2_ = 0.0674 |
| Final R indexes [all data] | R_1_ = 0.0289, wR_2_ = 0.0683 |
| Largest diff. peak/hole / e Å^-3^ | 0.12/-0.19 |
| Flack parameter | 0.09(8) |

**Supplementary Table 12** | Crystallographic data collection and structure refinement statistics.

|  | **CtdE-FAD** | **CtdE-FAD-3** |
| --- | --- | --- |
| **PDB entry** | 7KPQ | 7KPT |
| **Beamline** | Advanced Light Source (ALS)  502 beamline, Lawrence Berkeley National Laboratory, CA, USA | Advanced Light Source (ALS)  502 beamline, Lawrence Berkeley National Laboratory, CA, USA |
| **Resolution range (Å)** | 34.41-2.10 (2.18 – 2.10) | 33.82 -1.91 (1.98 – 1.91) |
| **Space group** | P 3_2_ 2 1 | C 1 2 1 |
| **Unit cell** |  |  |
| **a, b, c (Å)** | 89.00, 89.00, 108.52 | 157.28, 48.53, 67.64 |
| **α, β, γ (°)** | 90, 90, 120 | 90, 114.49, 90 |
| **Total reflections** | 29715 (1468) | 35699 (1774) |
| **Unique reflections** | 29506 (2881) | 34759 (2747) |
| **Multiplicity** | 9.7 (9.2) | 4.5 (4.6) |
| **Completeness (%)** | 99.64 (98.83) | 95.62 (76.45) |
| **Mean I/sigma(I)** | 8.5 (8.1) | 13.2 (7.7) |
| **Wilson B-factor** | 24.81 | 18.48 |
| **R-merge** | 0.069 (1.000) | 0.078 (0.849) |
| **R-meas** | 0.073 (1.070) | 0.088 (0.949) |
| **R-pim** | 0.023 (0.348) | 0.040 (0.421) |
| **Reflections used in refinement** | 29451 (2882) | 34759 (2747) |
| **Reflections used for R-free** | 1414 (139) | 1766 (137) |
| **R-work** | 0.1853 (0.2348) | 0.1697 (0.1925) |
| **R-free** | 0.2204 (0.2614) | 0.2014 (0.2359) |
| **Number of non-hydrogen atoms** | 3307 | 3654 |
| **macromolecules** | 2974 | 3211 |
| **ligands** | 53 | 90 |
| **solvent** | 280 | 353 |
| **RMS (bonds) (Å)** | 0.004 | 0.008 |
| **RMS (angles) (°)** | 0.810 | 1.010 |
| **Ramachandran favored (%)** | 98.41 | 98.77 |
| **Ramachandran allowed (%)** | 1.59 | 1.23 |
| **Ramachandran outliers (%)** | 0.00 | 0.00 |
| **Rotamer outliers (%)** | 0.32 | 0.30 |
| **Clashscore** | 1.34 | 3.39 |
| **Average B-factor (Å^2^)** | 32.37 | 22.04 |
| **macromolecules** | 32.28 | 21.13 |
| **ligands** | 23.15 | 18.34 |
| **solvent** | 35.16 | 31.33 |
| **Number of TLS groups** | 4 | 4 |
| **RSCC (FAD)** | 0.96 | 0.97 |
| **RSCC (substrate 3)** | - | 0.93 |
| **RSR (FAD)** | 0.12 | 0.09 |
| **RSR (substrate 3)** | - | 0.15 |

Values in brackets refer to the high-resolution outer shell.

**Supplementary Table** **13** | Structural homologs of CtdE identified by DALI server^2^.

| **# No** | **Protein** | **PDB-Chain** | **Z score** | **rmsd** | **Number of structurally equivalent residues** | **Total number of amino acids** | **%identity** |
| --- | --- | --- | --- | --- | --- | --- | --- |
| 1 | **PhqK** | 6pvi-A | 48.0 | 2.2 | 382 | 438 | 35 |
| 2 |  | 6pvg-A | 47.0 | 2.0 | 383 | 445 | 35 |
| 3 |  | 6pvf-A | 47.0 | 2.2 | 383 | 444 | 35 |
| 4 |  | 6pvh-A | 47.0 | 2.2 | 383 | 444 | 35 |
| 5 |  | 6pvj-A | 46.9 | 2.2 | 383 | 444 | 35 |
| 6 | **PhzS** | 2rgj-A | 39.1 | 2.4 | 335 | 376 | 22 |
| 7 |  | 3c96-A | 39.1 | 2.5 | 335 | 381 | 23 |
| 8 | **3-Hydroxybenzoate 6-hydroxylase (3HB6H)** | 4bk3-A | 38.3 | 2.9 | 340 | 395 | 21 |
| 9 |  | 5hym-A | 38.2 | 2.9 | 340 | 395 | 21 |
| 10 |  | 4bjz-A | 38.2 | 3.0 | 341 | 395 | 21 |
| 11 |  | 4bjy-A | 38.0 | 3.0 | 340 | 393 | 21 |
| 12 |  | 4bk2-A | 38.0 | 2.9 | 339 | 393 | 21 |
| 13 |  | 4bk1-A | 37.4 | 3.0 | 336 | 389 | 21 |
| 14 | **HpxO** | 3rp7-A | 36.8 | 2.7 | 334 | 389 | 19 |
| 15 |  | 3rp6-A | 36.7 | 2.8 | 336 | 392 | 19 |
| 16 | **2-methyl-3-hydroxypyridine-5-carboxylic acid oxygenase (MHPCO)** | 4h2p-C | 36.7 | 2.7 | 333 | 370 | 20 |
| 17 |  | 4h2p-B | 36.7 | 2.7 | 333 | 371 | 20 |
| 18 |  | 4h2p-D | 36.6 | 2.7 | 332 | 369 | 20 |
| 19 |  | 4h2p-A | 36.6 | 2.7 | 333 | 371 | 20 |
| 20 |  | 3alj-A | 36.6 | 2.7 | 332 | 369 | 20 |
| 21 |  | 4h2q-A | 36.6 | 2.7 | 333 | 370 | 20 |
| 22 |  | 5hxi-A | 36.5 | 2.8 | 332 | 369 | 20 |
| 23 |  | 3all-A | 36.5 | 2.8 | 333 | 370 | 20 |
| 24 |  | 3alm-A | 36.5 | 2.7 | 333 | 370 | 20 |
| 25 | **HpxO** | 3rp8-A | 36.5 | 2.7 | 334 | 381 | 19 |
| 26 | **MHPCO** | 4jy3-B | 36.5 | 2.7 | 333 | 370 | 20 |
| 27 | **TetX** | 5tue-B | 36.5 | 2.7 | 337 | 366 | 20 |
| 28 | **MHPCO** | 4h2n-A | 36.5 | 2.8 | 333 | 370 | 20 |
| 29 | **TetX** | 5tuf-B | 36.5 | 2.7 | 338 | 371 | 20 |
| 30 | **MHPCO** | 4jy2-A | 36.4 | 2.7 | 333 | 370 | 20 |

**Supplementary Table 14** | Calculation of the binding free energy (kcal mol^-1^) of β-face pose (FAD on the β-face of substrate) and α-face pose (FAD on the α-face of substrate) of substrates in CtdE.

| **System** | $\boldsymbol{\Delta E}_{\boldsymbol{vdw}}$ | $\boldsymbol{\Delta E}_{\boldsymbol{ele}}$ | $\boldsymbol{\Delta G}_{\boldsymbol{pol}}$ | $\boldsymbol{\Delta G}_{\boldsymbol{nopol}}$ | $\boldsymbol{\Delta G}_{\boldsymbol{bind}}$ |
| --- | --- | --- | --- | --- | --- |
| **2** in β-face pose | -53.38 | -14.58 | 18.76 | -6.28 | -55.48 |
| **2** in α-face pose | -40.14 | -19.62 | 21.11 | -4.74 | -40.06 |
| **3** in β-face pose | -65.96 | -15.65 | 15.38 | -7.55 | -73.78 |
| **3** in α-face pose | -65.51 | -14.32 | 19.86 | -7.56 | -67.52 |

**Supplementary Figures**

**Supplementary Fig. 1** | Representative prenylated indole alkaloids. (**a**) Spirooxindole PIAs possessing a bicyclo[2.2.2]diazaoctane ring, and (**b**) spirooxindoles PIAs lack the bicyclo[2.2.2]diazaoctane ring.

**Supplementary Fig. 2** | FPMOs of NotB^3^, PhqK^4^, NotI/NotI’^5^, BvnB^6^, and FqzB^7^ catalyzed reactions.


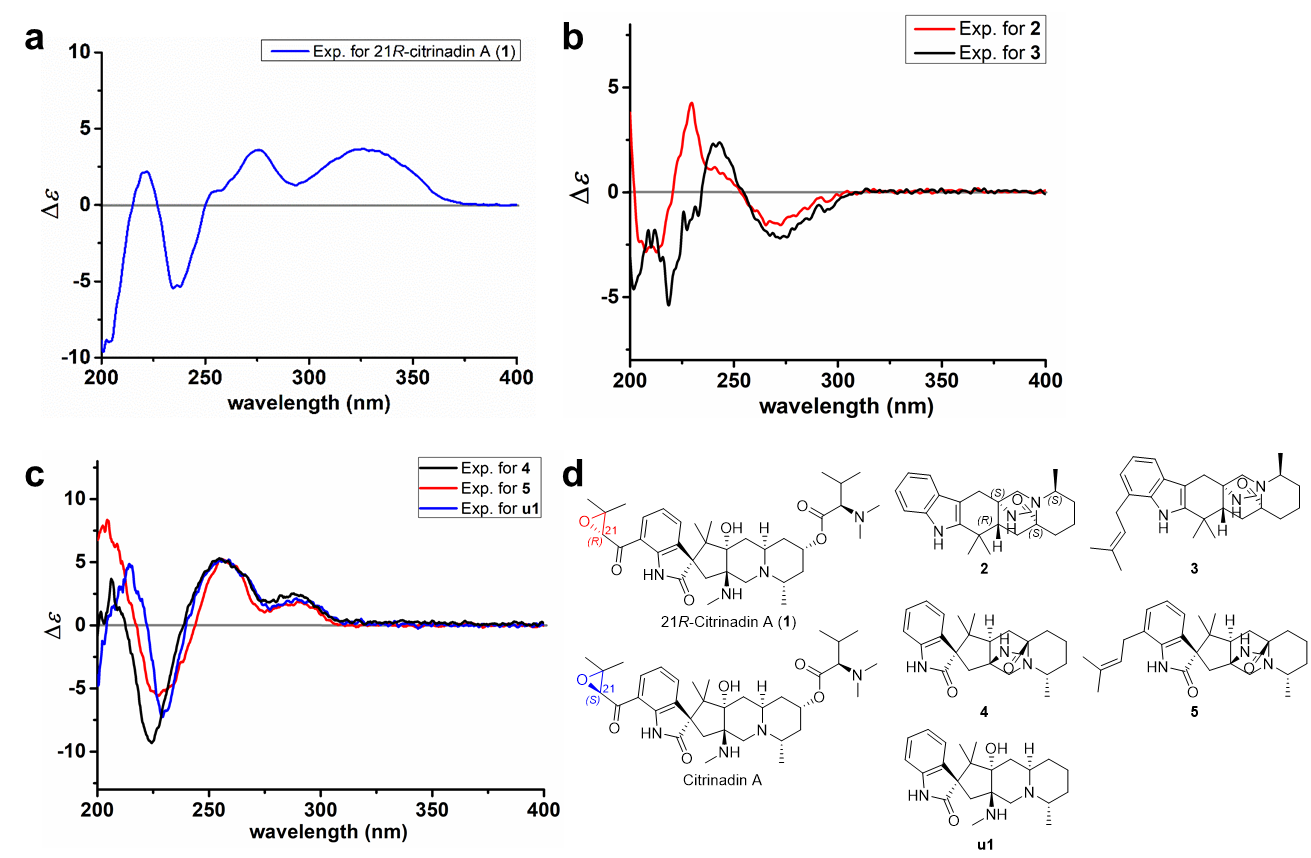


**Supplementary Fig. 3** | ECD spectra analysis of **1**-**5** and **u1**. (**a**) Experimental (Exp.) ECD spectra of 21*R*-citrinadin A (**1**), ECD spectrum of **1** is the same as that of 21*R*-citrinadin A, which is differ from that of citrinadin A in the reference^1^. (**b**) Experimental (Exp.) ECD spectra of **2** and **3**; (**c**) Experimental (Exp.) ECD spectra of **4**, **5**, and **u1**. (**d**) The chemical structures of citrinadin A, **1**-**5**, and **u1.**


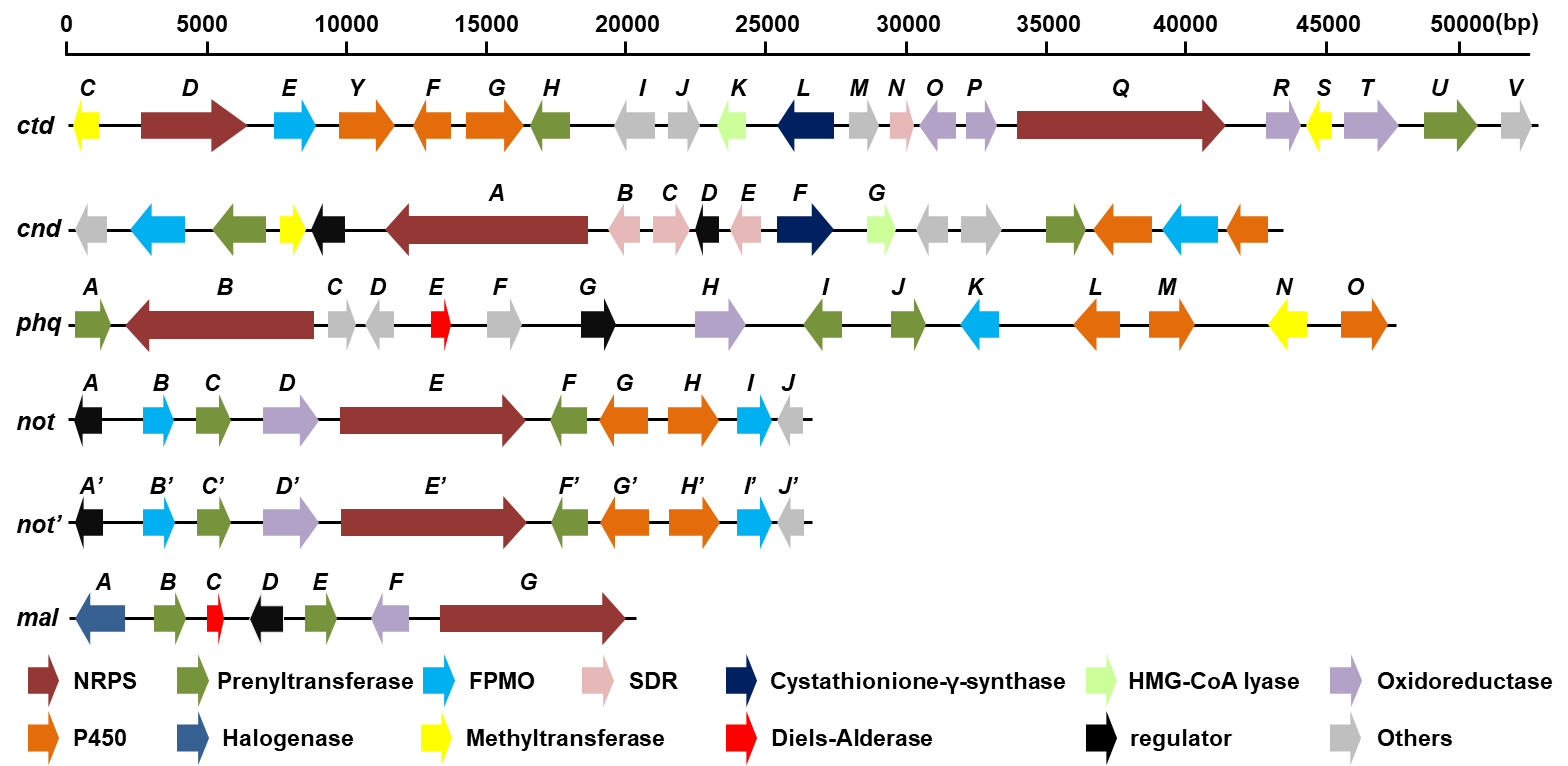


**Supplementary Fig. 4** | Comparison analysis of the biosynthetic gene clusters of PIAs^5^. 21*R*-citrinadin A (*ctd* in *Penicillin citrinum* ATCC 9849), citrinadin A (*cnd* in *P. citrinum* DSM1997)^8^, paraherquamides (*phq* in *P. fellutanum* ATCC20841), (-)-notoamide A (*not* in *Aspergillus protuberus* MF297-2), (+)-notoamide A (*not’* in *A. versicolor* NRRL35600), and malbrancheamide (*mal* in *Malbranchea aurantiaca* RRC1813).^9^ NRPS: nonribosomal peptide synthetase; FPMO: flavoprotein monooxygenase; P450: cytochrome P450; SDR: Short-chain dehydrogenase.


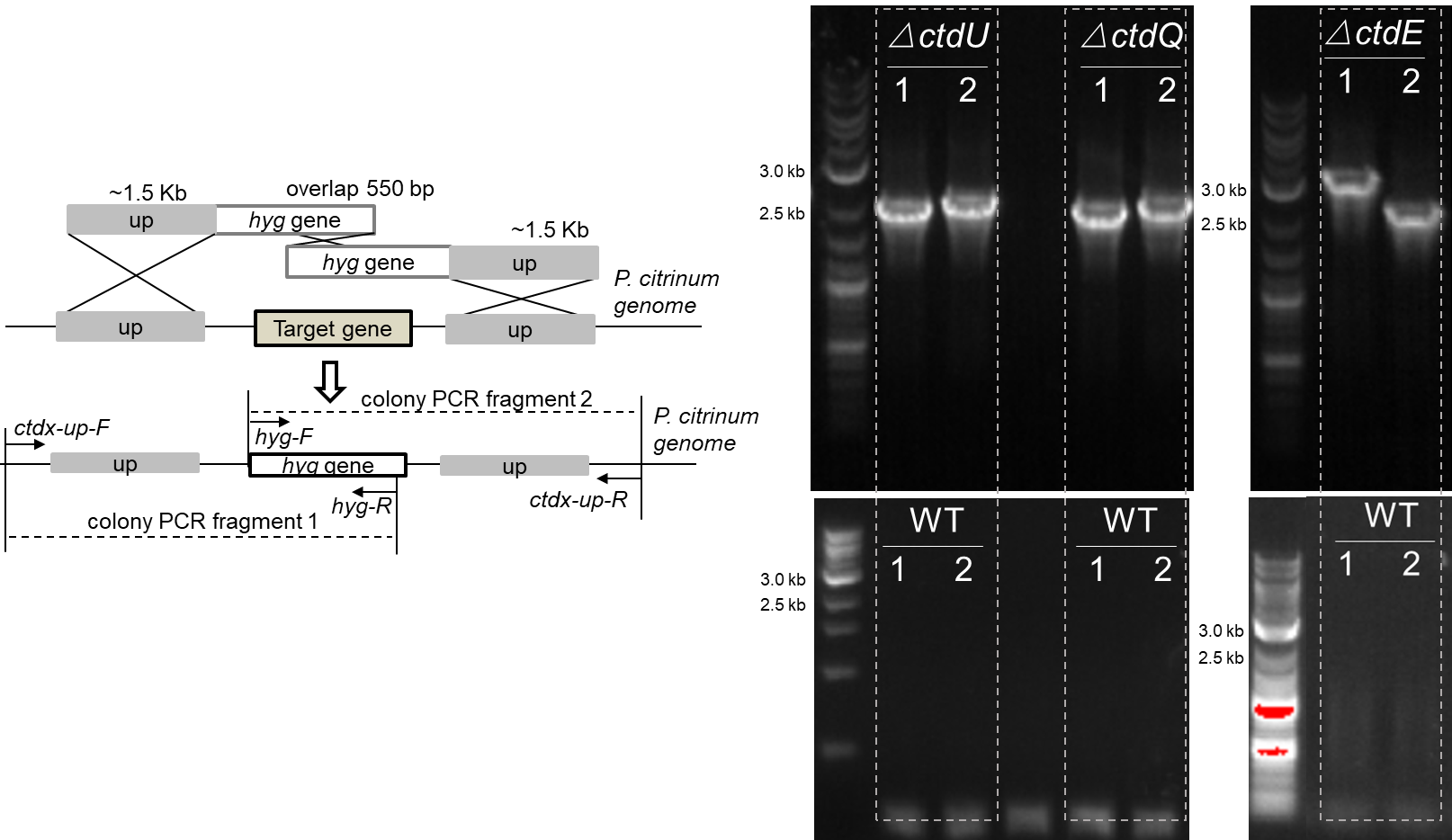


**Supplementary Fig. 5** | PCR confirmation of *ctd* mutants. *ctd* transformants were screened by PCR using primers *ctdx-up-F* and *hyg-R* for up-stream screening, primers *hyg-F* and *ctdx-dn-R* for down-stream screening. All experiments were repeated independently with similar results for three times.


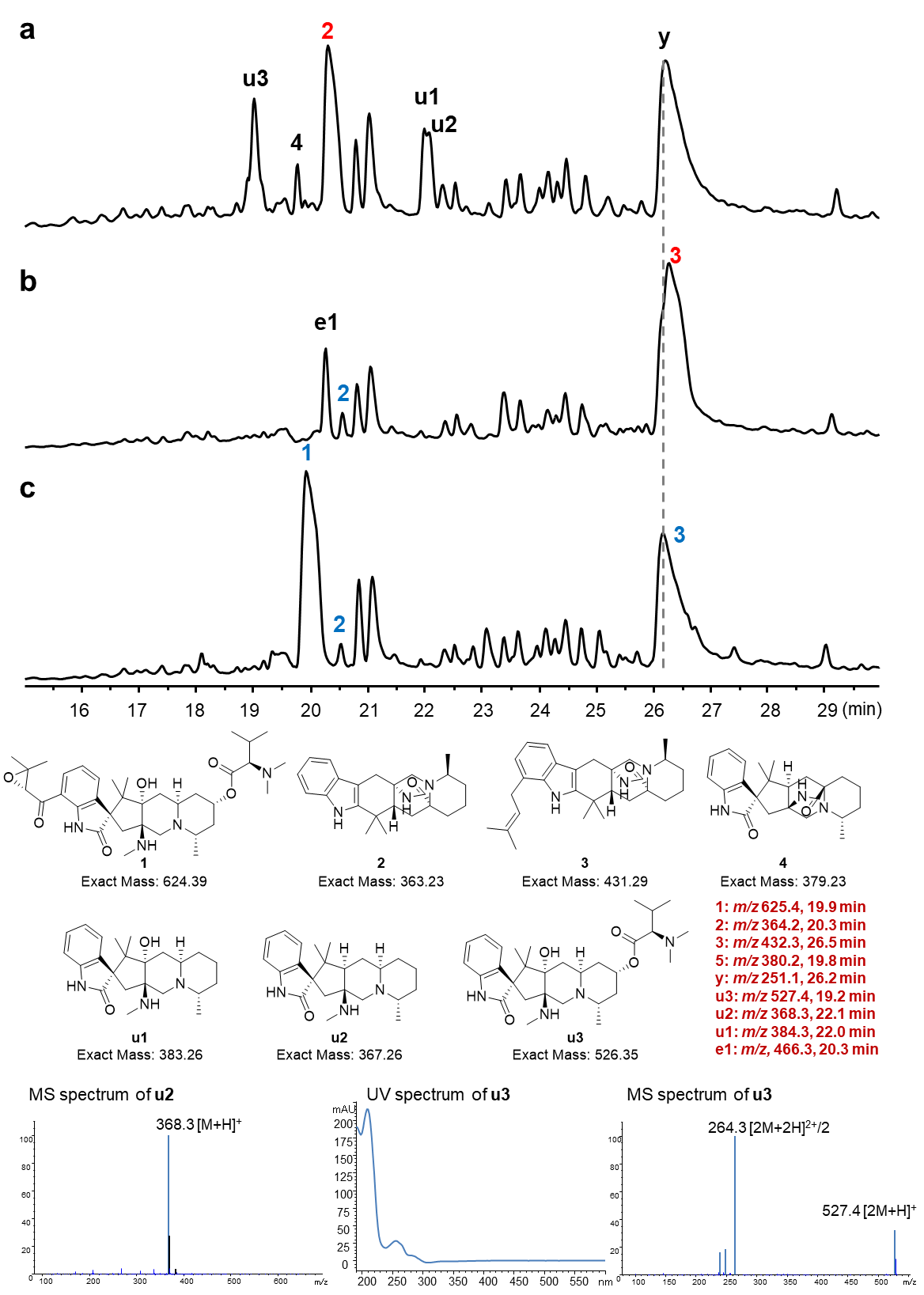


**Supplementary Fig. 6** | LCMS traces of metabolic extracts from (**a**) △*ctdU* mutant, (**b**) △*ctdE* mutant, and (**c**) wild-type of *P. citrinum*. The structures of intermediates **u2** and **u3** are proposed based on their MS and UV spectra analysis. Compound **y** represents citrinin^10^.


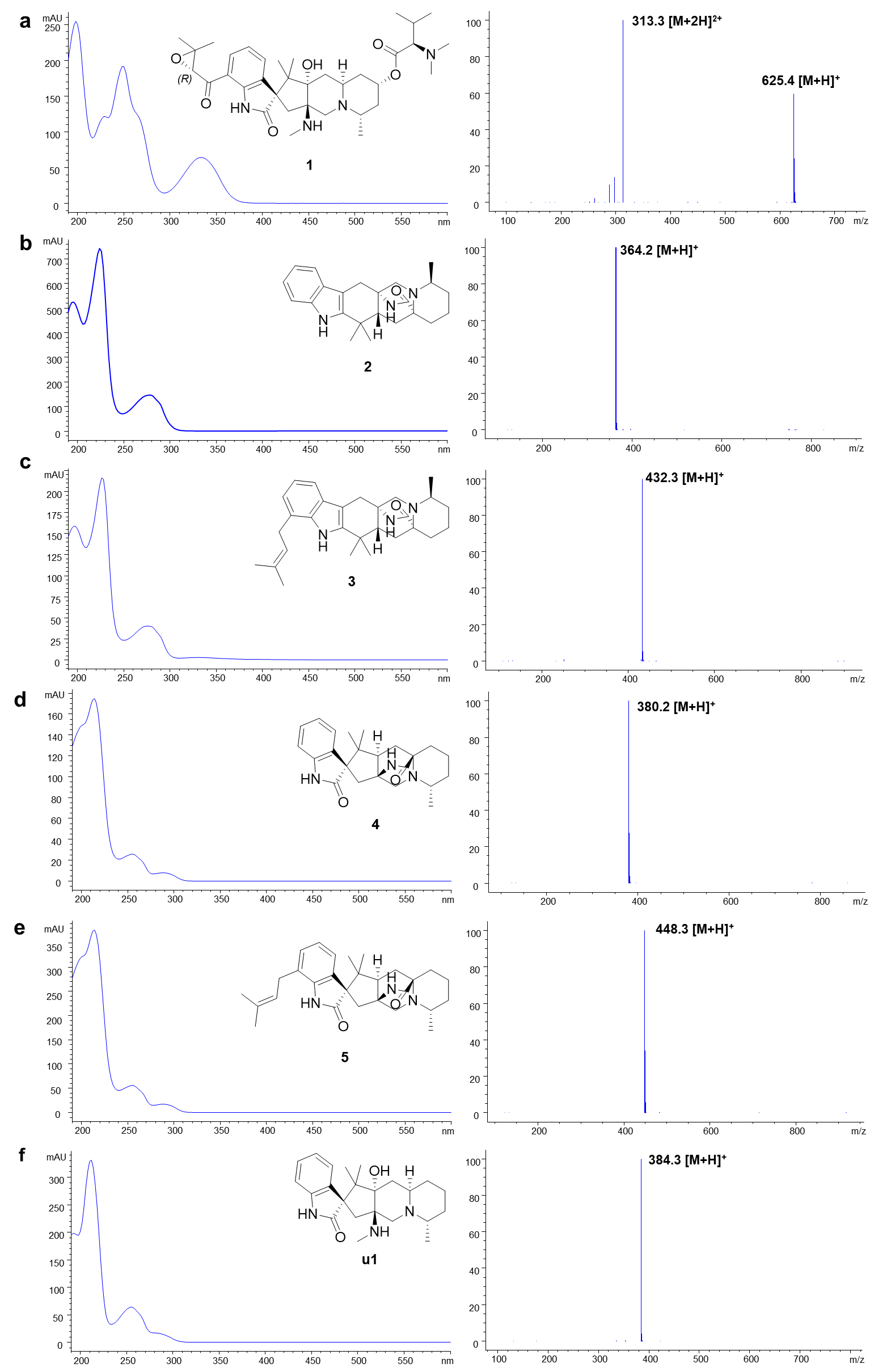


**Supplementary Fig. 7** | UV and MS spectra of compounds (**a**) **1**, (**b**) **2**, (**c**) **3**, (**d**) **4**, (**e**) **5**, and (**f**) **u1**.


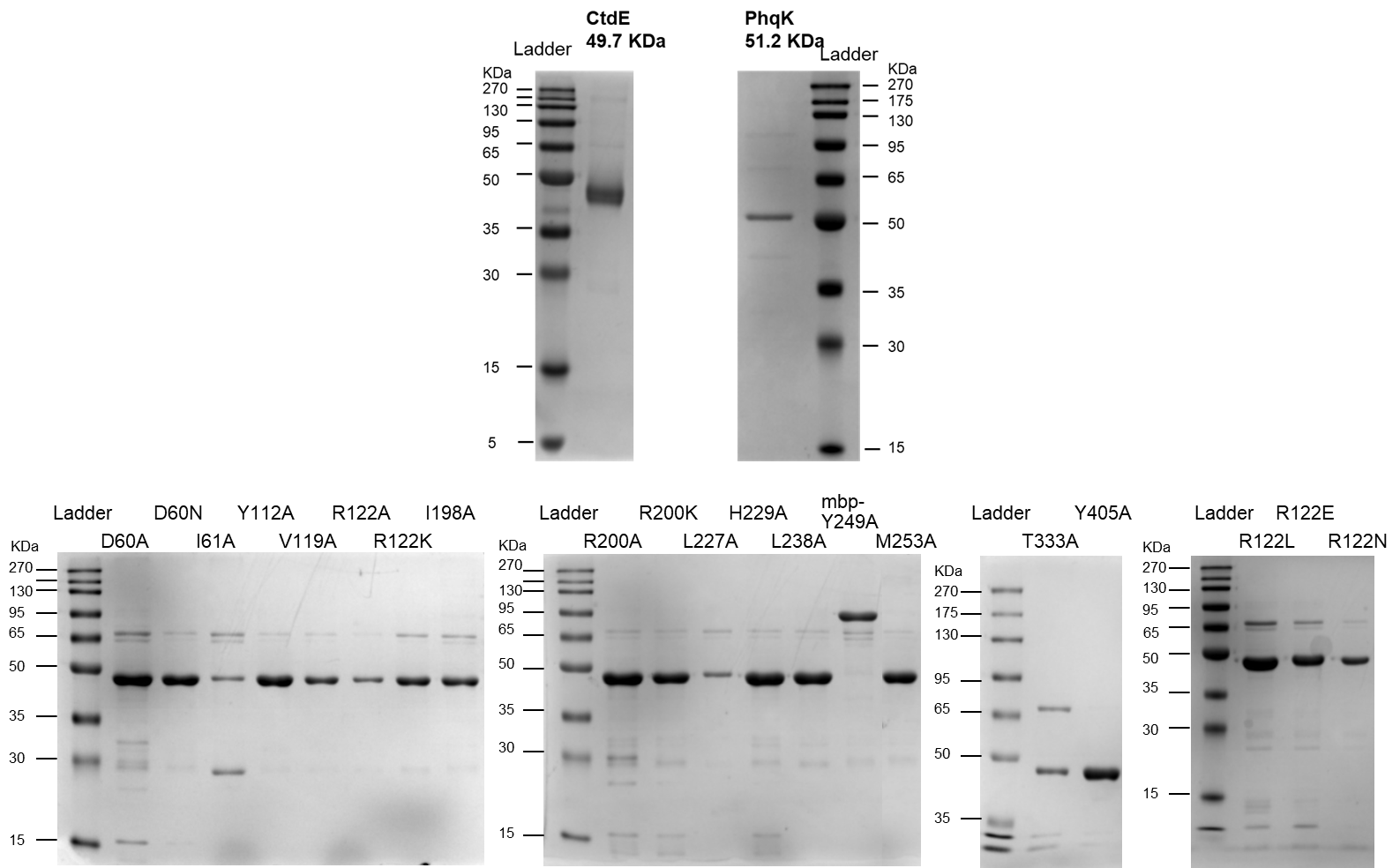


**Supplementary Fig. 8** | SDS-PAGE (12%) analysis of purified CtdE, PhqK and CtdE site-directed mutant enzymes. All experiments were repeated independently with similar results for three times.

**Supplementary Fig. 9** | Kinetic analysis of CtdE catalysed reactions of (**a**) **2** and (**b**) **3**, and standard curves of compounds (**c**) **4** and (**d**) **5**. For all measurements in **a** and **b**, data represent the average of triplicate independent experiments (centre values, mean; error bars, s.d.; n = 3). Reaction mixtures containing 2 μM CtdE, 5 mM NADH, 100 uM FAD and different concentrations of **2** and **3** (20 μM to 1.0 mM), respectively, were incubated at 28 °C for 10 min, 15 min, and 20 min, respectively. .

**Supplementary Fig. 10** | Summary of biosynthetic pathways in PIAs reveal evolutionary branches. **a**, (+)-malbrancheamide^11^ (*M. aurantiaca*) and spiromalbramide (spiromalbramide was only isolated from *M. graminicola*). **b**, paraherquamide N^4^. **c**, (+)-brevianamides^6^, **d**, 21*R*-citrinadin A.


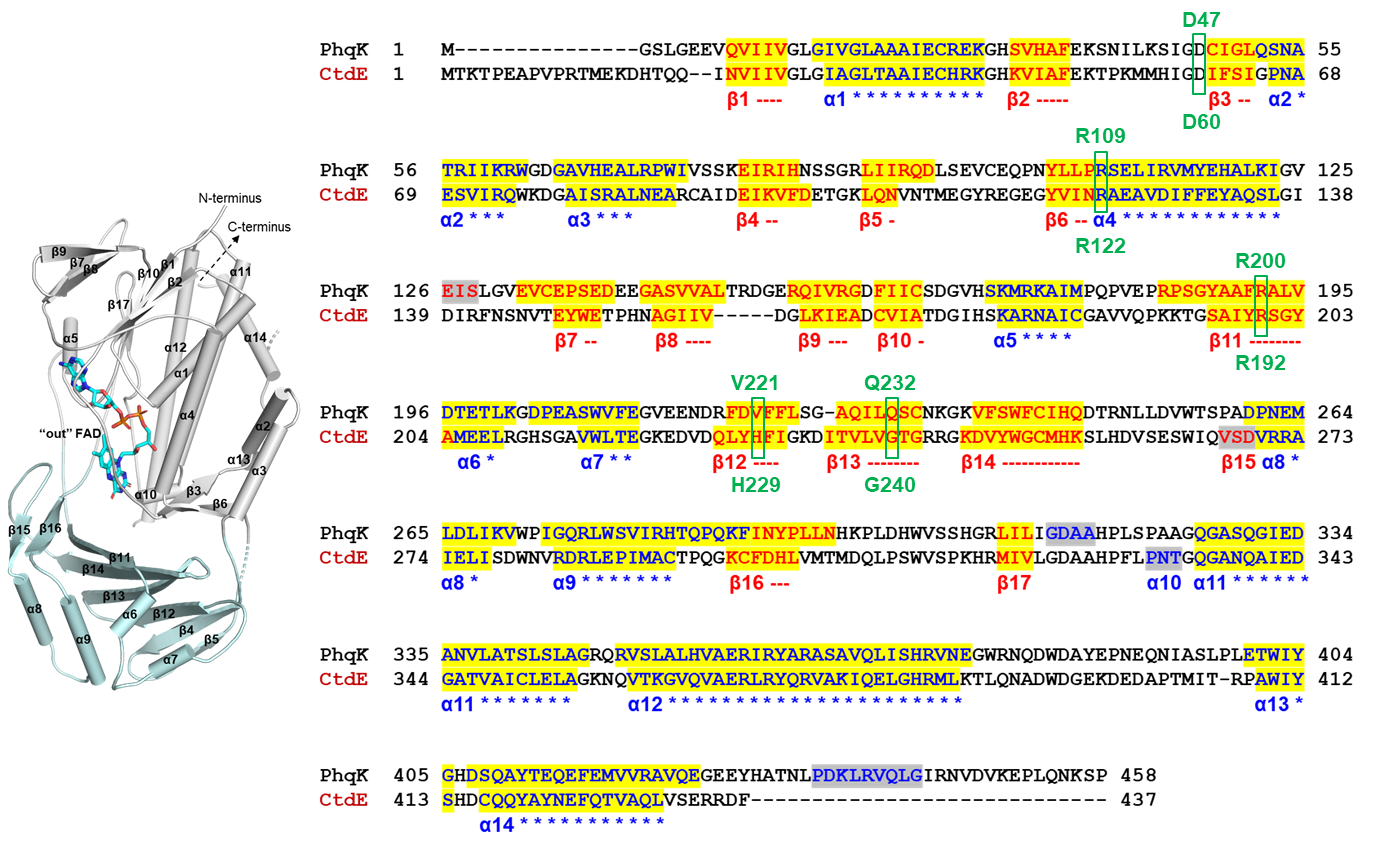


**Supplementary Fig. 11** | Alignment of CtdE and its structural homolog PhqK, the α-helix is colored in blue and the β-sheet is colored in red.

**
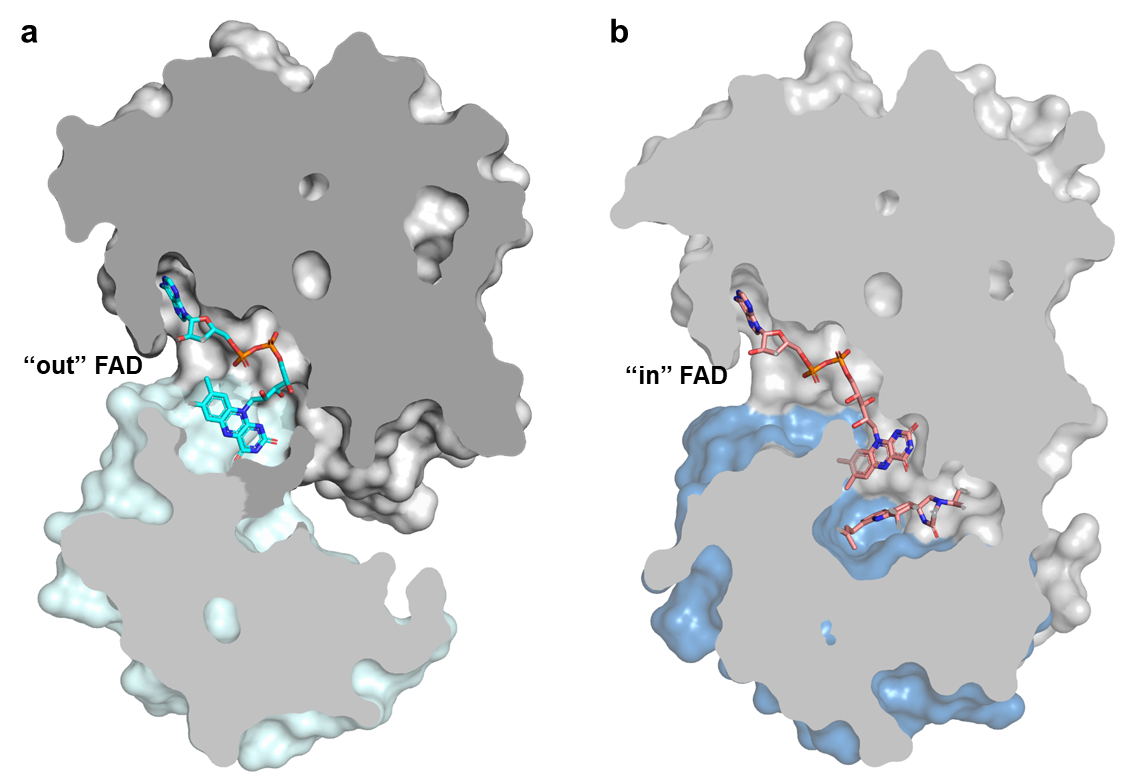
**

**Supplementary Fig. 12** | The cut-way view of surface representation of (**a**) CtdE-FAD complex shows the “out” FAD with an open substrate loading channel, and (**b**) CtdE-FAD-**3** complex shows “in” FAD and substrate **3** binding pocket which closes the substrate loading channel.

**
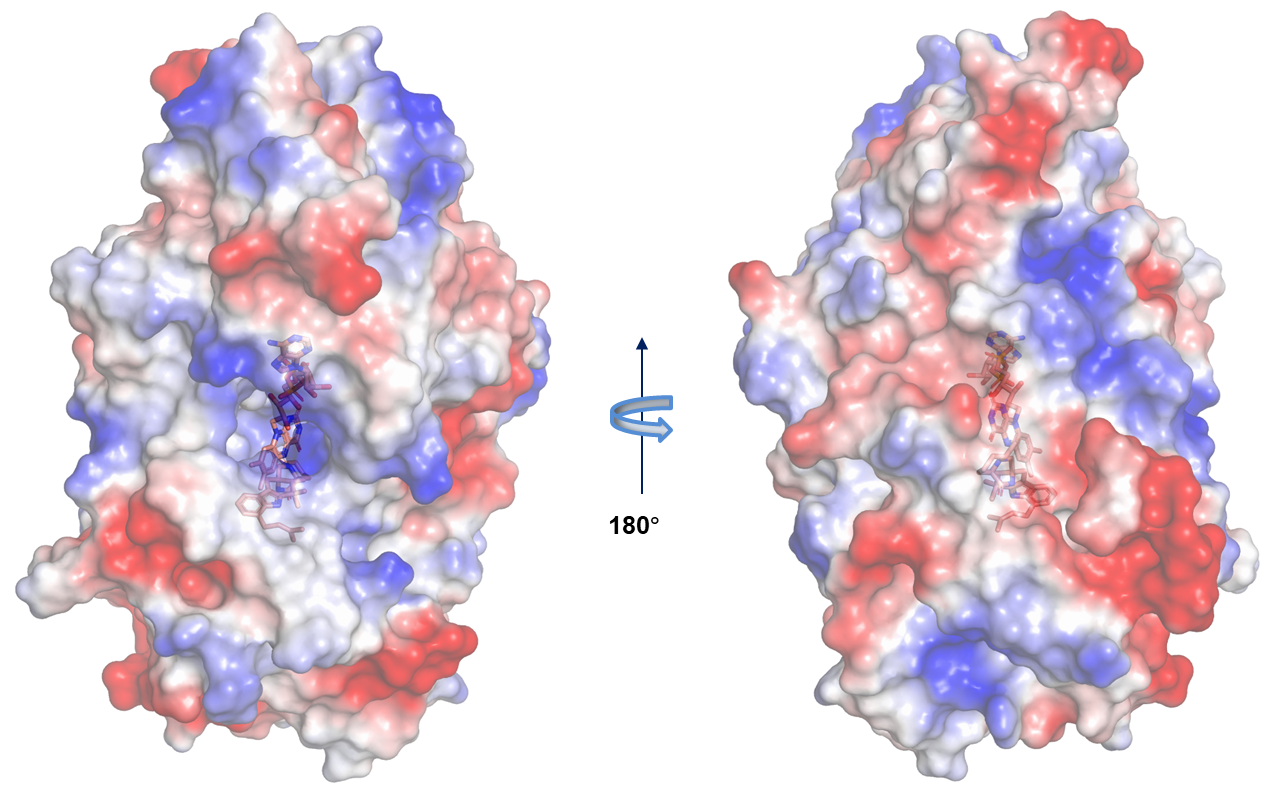
**

**Supplementary Fig. 13** | Electrostatic surface potential of CtdE-FAD-**3** complex.

**
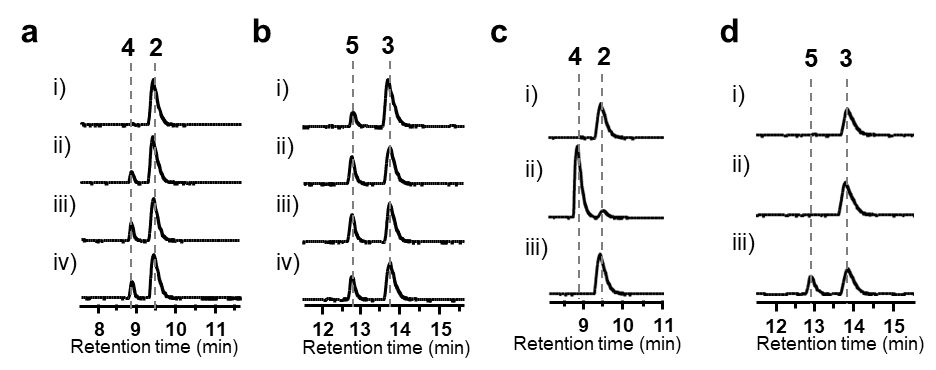
**

**Supplementary Fig. 14** | *In vitro* assays of CtdE and PhqK. **a**, *In vitro* assay of 2 μM CtdE with 500 μM **2**, 5 mM NADH, and 100 μM FAD in (i) Bis-tris buffer pH 6.0 (ii) Tris-HCl buffer pH 7.0, (iii) HEPES buffer pH 7.6 and (iv) Tris-HCl buffer pH 7.6. **b**, *In vitro* assay of 2 μM CtdE with 500 μM **3**, 5 mM NADH, and 100 μM FAD in (i) Bis-tris buffer pH 6.0, (ii) Tris-HCl buffer pH 7.0, (iii) HEPES buffer pH 7.6 and (iv) Tris-HCl buffer pH 7.6. **c**, *In vitro* assay of 500 μM **2**, 5 mM NADH, and 100 μM FAD with (i) 20 μM PhqK (ii) 20 μM CtdE and (iii) 20 μM boiled PhqK in Tris-HCl buffer pH 7.6. **d**, *In vitro* assay of 500 μM **2**, 5 mM NADH, 100 μM FAD with (i) 20 μM PhqK (ii) 20 μM CtdE and (iii) 20 μM boiled PhqK in Tris-HCl buffer pH 7.6.

**
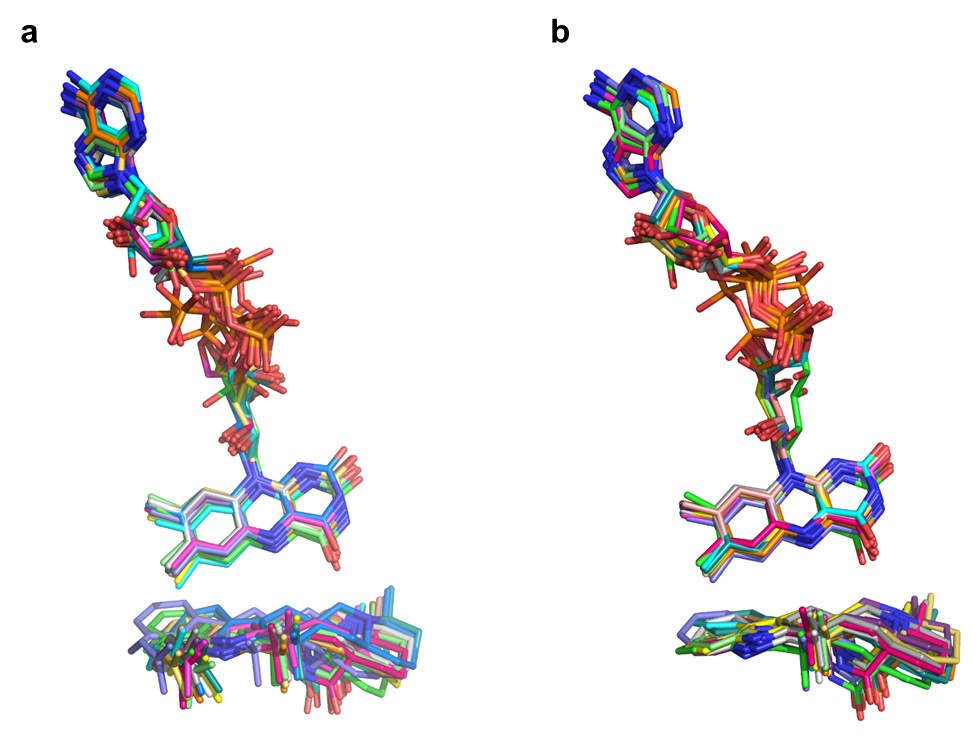
**

**Supplementary Fig. 15** | Overlay of 10 representative binding poses from 500 ns MD simulations of (**a**) **3** and (**b**) **2**.

**
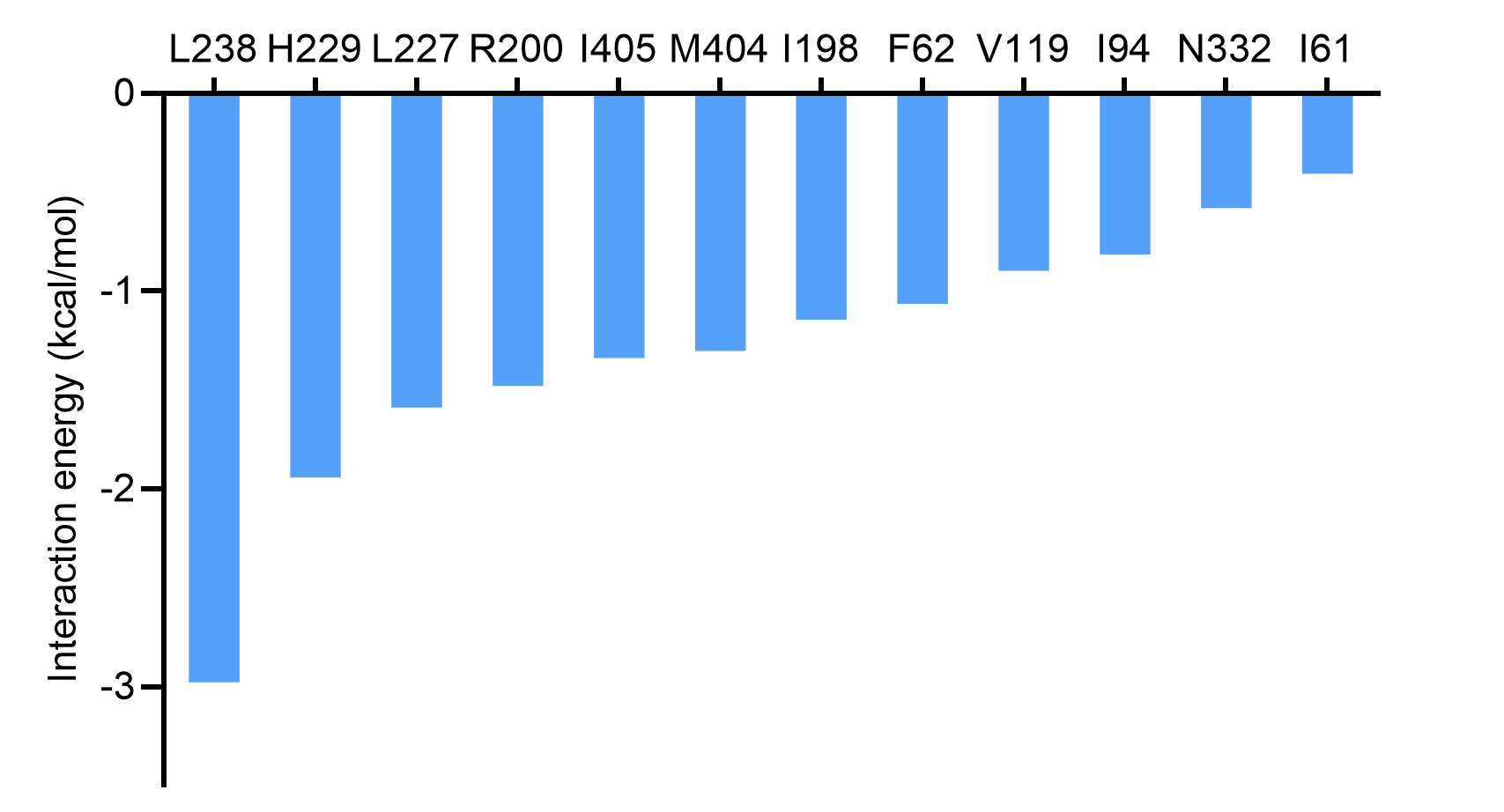
**

**Supplementary Fig. 16** | Per-residue free energy decomposition of **3**.


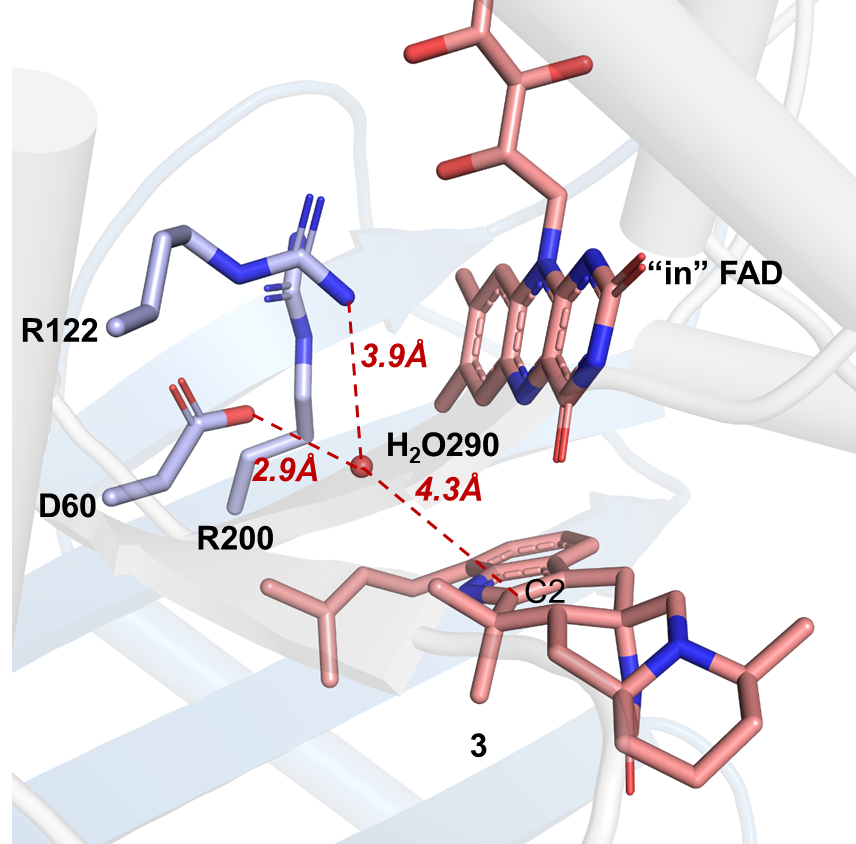


**Supplementary Fig. 17** | The water molecule 290 in the active side of CtdE-FAD-**3** structure.


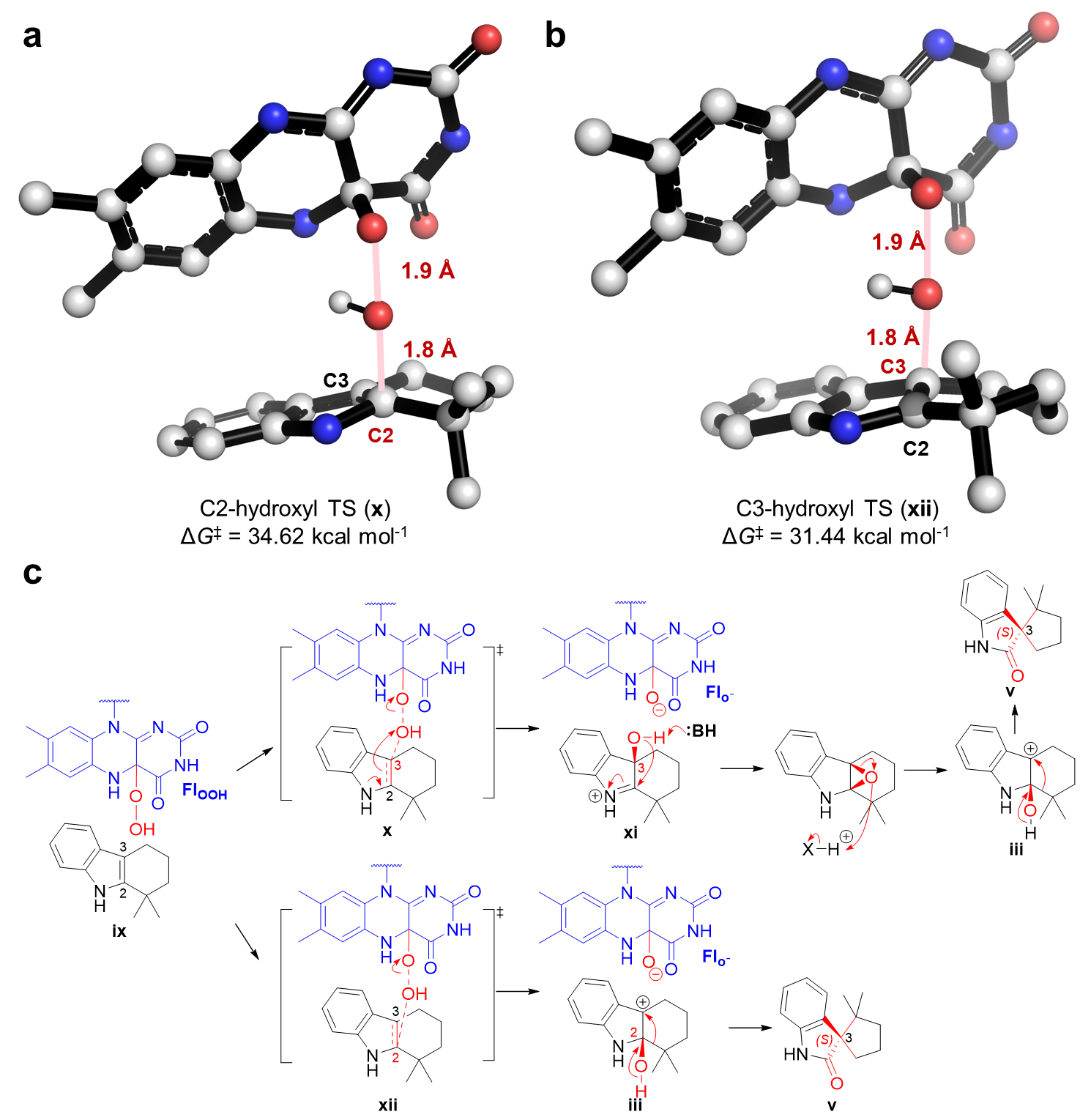


**Supplementary Fig. 18** | DFT calculated Gibbs free energies for the C2 and C3 hydroxylation steps by using the truncated structures. **a**, Structure of truncated C2-hydroxyl transition state (TS, **x**). **b**, Structure of the truncated C3-hydroxyl TS (**xii**). **c**, Proposed pathways of C2 and C3 hydroxylations by C4a-hydroperoxide flavin.


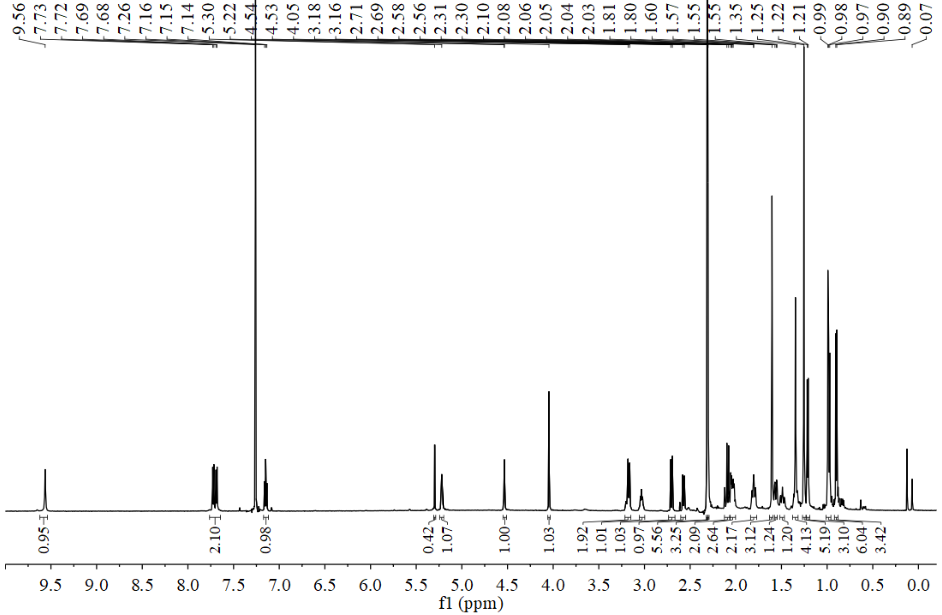

**Supplementary Fig. 19-1**| ^1^H NMR spectrum of **1** in CDCl_3_.


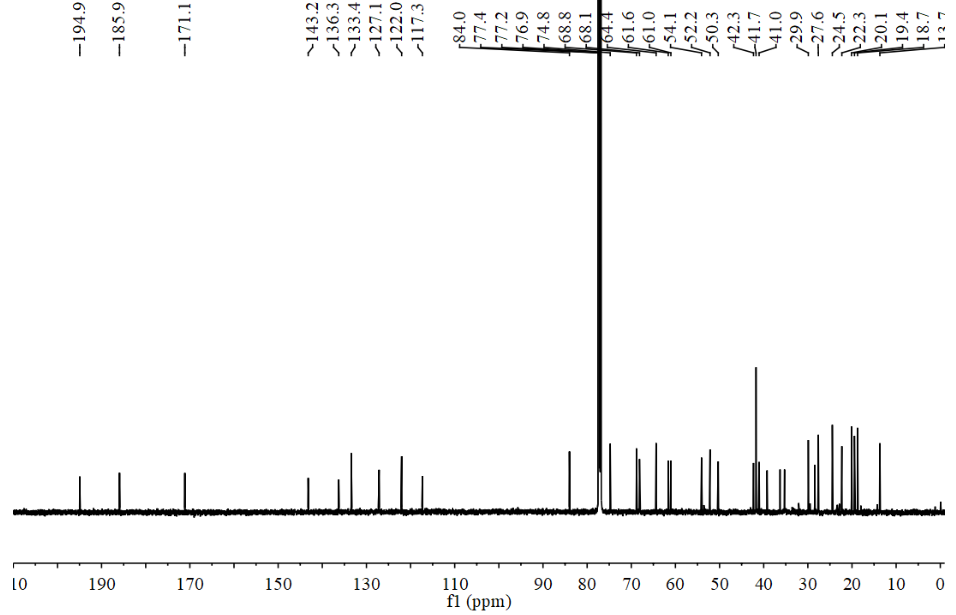

**Supplementary Fig. 19-2**| ^13^C NMR spectrum of **1** in CDCl_3_.


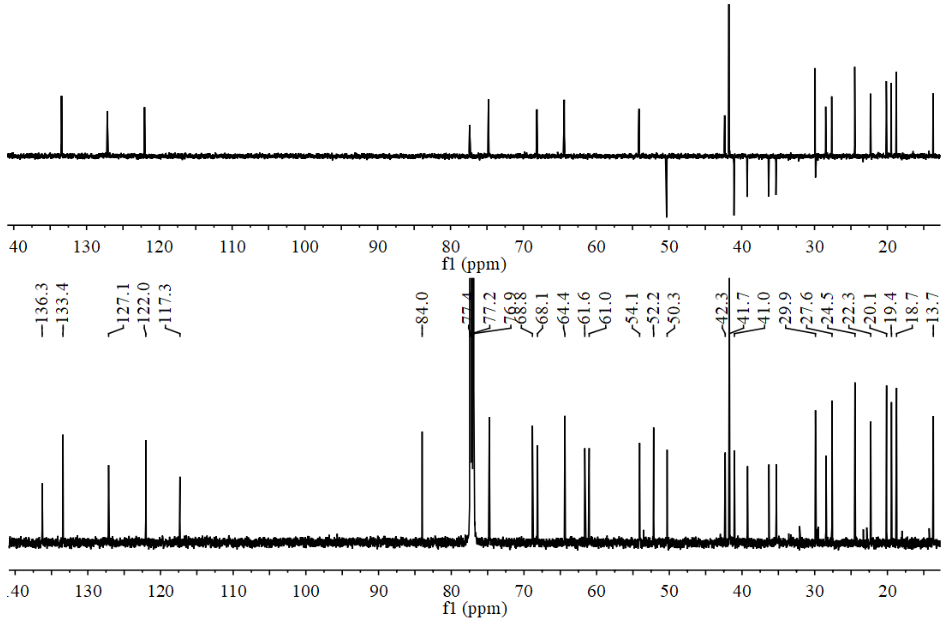

**Supplementary Fig. 19-3** | DEPT135 and ^13^C NMR spectra of **1** in CDCl_3_.


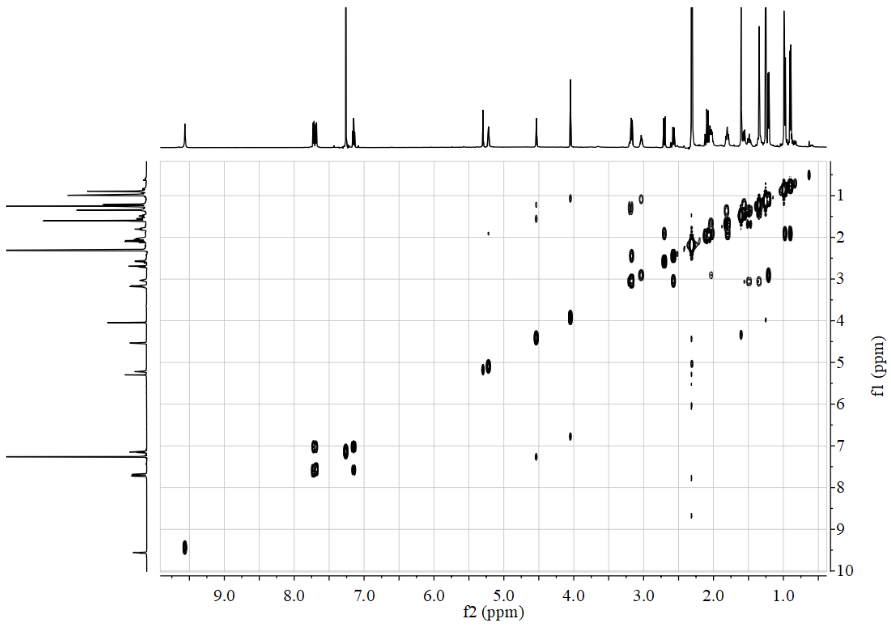

**Supplementary Fig. 19-4** | ^1^H-^1^H COSY NMR spectrum of **1** in CDCl_3_.


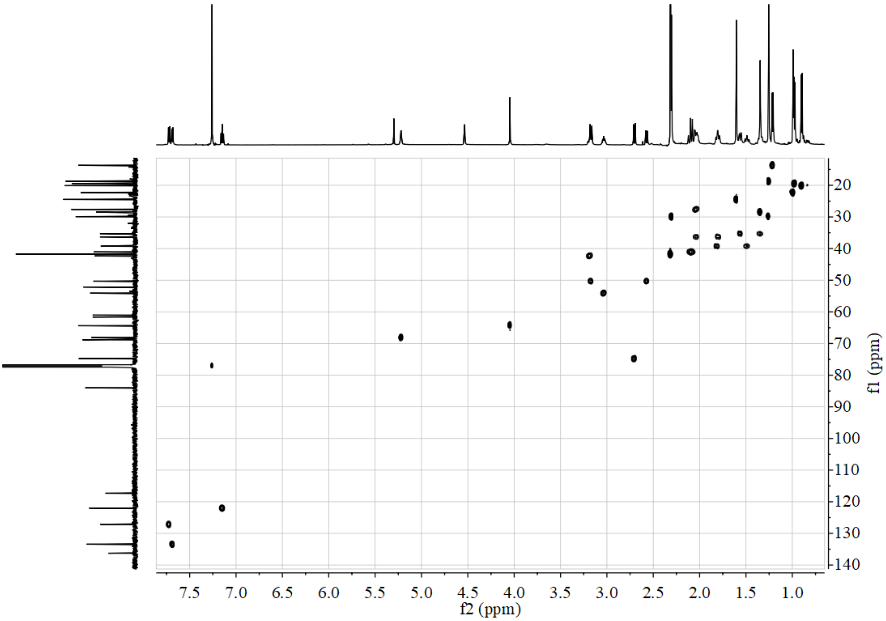

**Supplementary Fig. 19-5** | HSQC NMR spectrum of **1** in CDCl_3_.


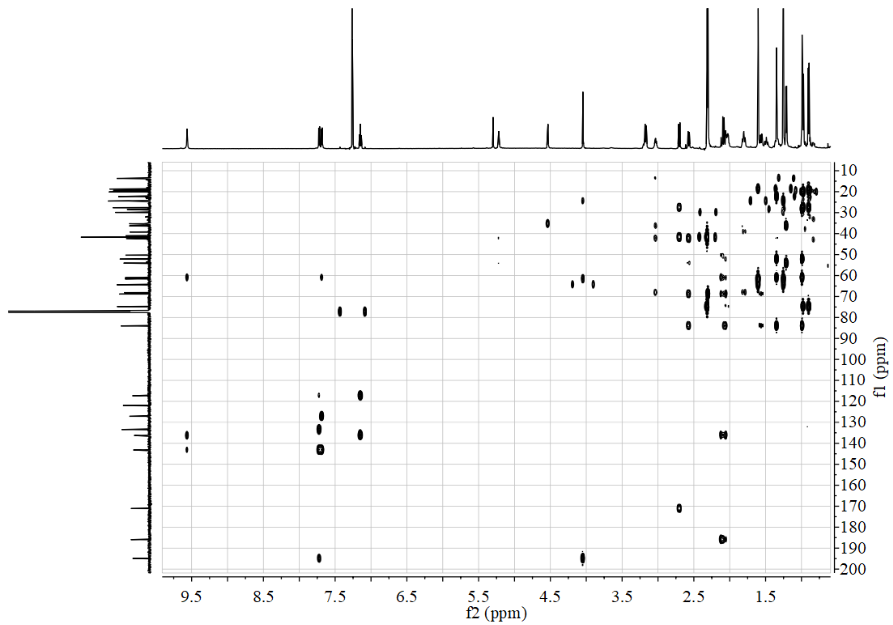

**Supplementary Fig. 19-6** | HMBC NMR spectrum of **1** in CDCl_3_.


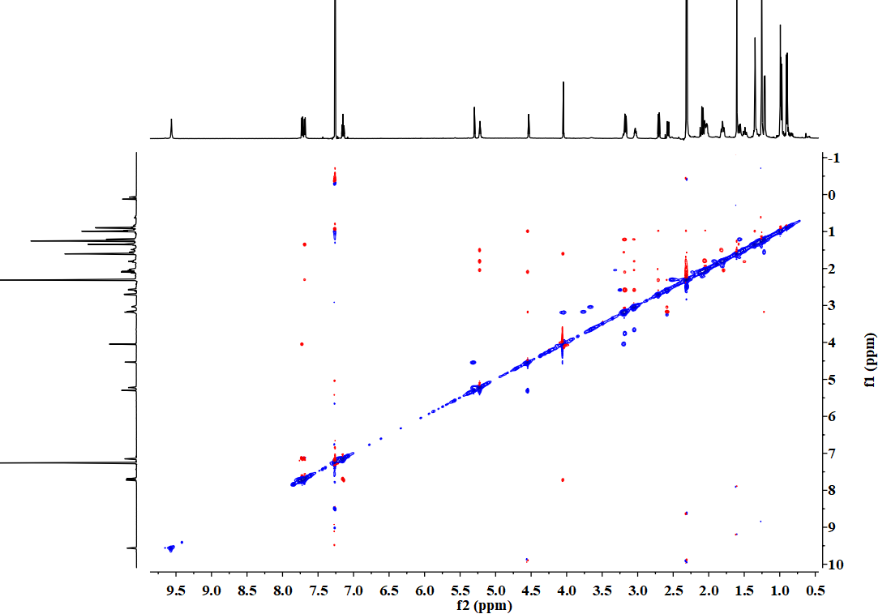

**Supplementary Fig. 19-7** | ROESY NMR spectrum of **1** in CDCl_3_.


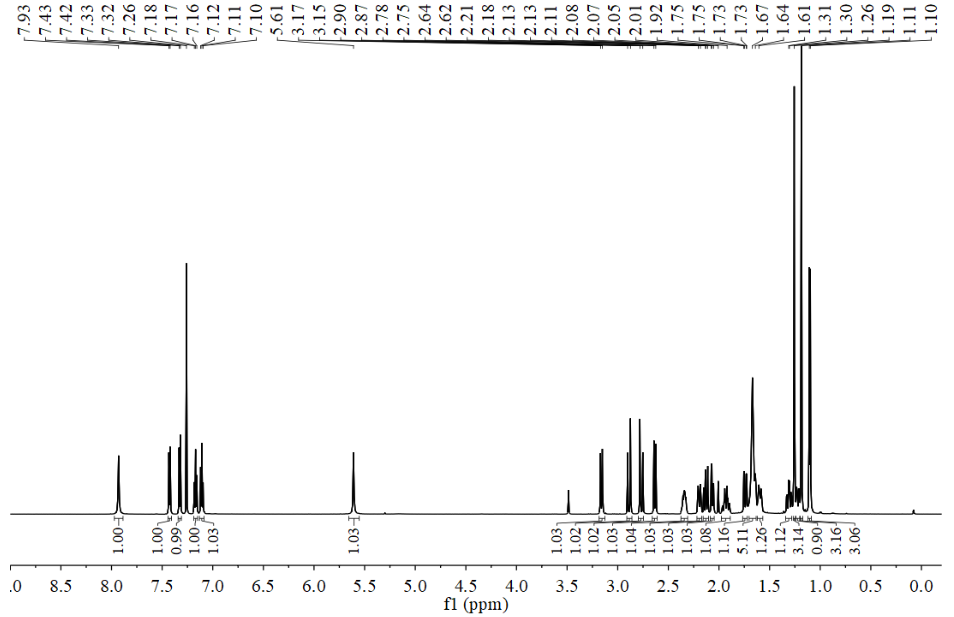

v

**Supplementary Fig. 20-1** | ^1^H NMR spectrum of **2** in CDCl_3_.


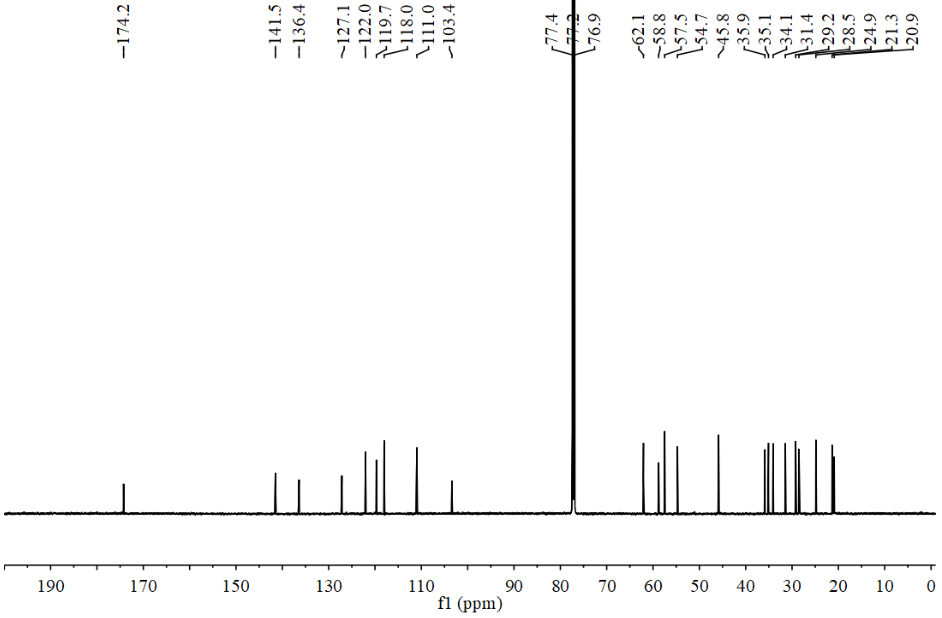

v

**Supplementary Fig. 20-2** | ^13^C NMR spectrum of **2** in CDCl_3_.


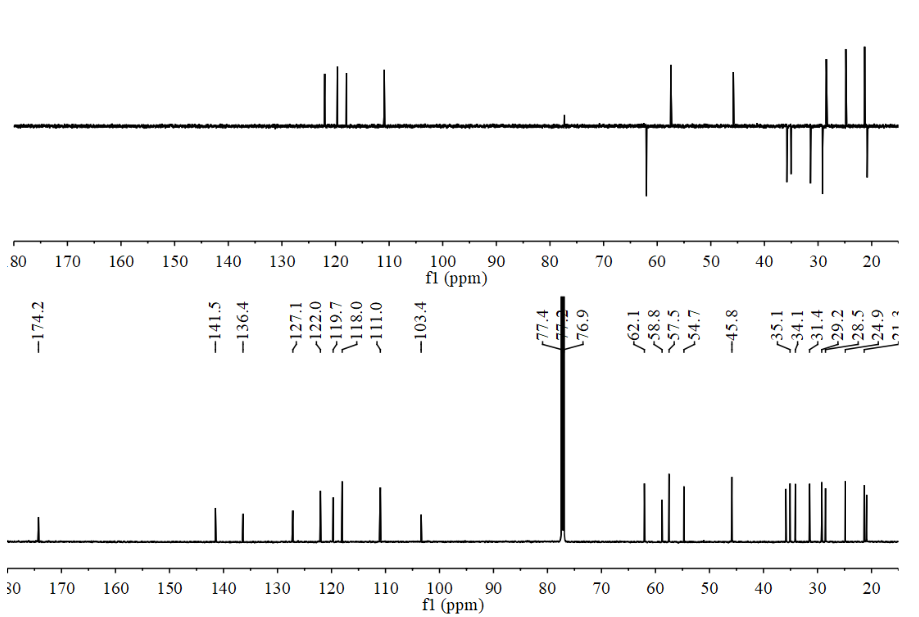

v

**Supplementary Fig. 20-3** | DEPT135 and ^13^C NMR spectra of **2** in CDCl_3_.


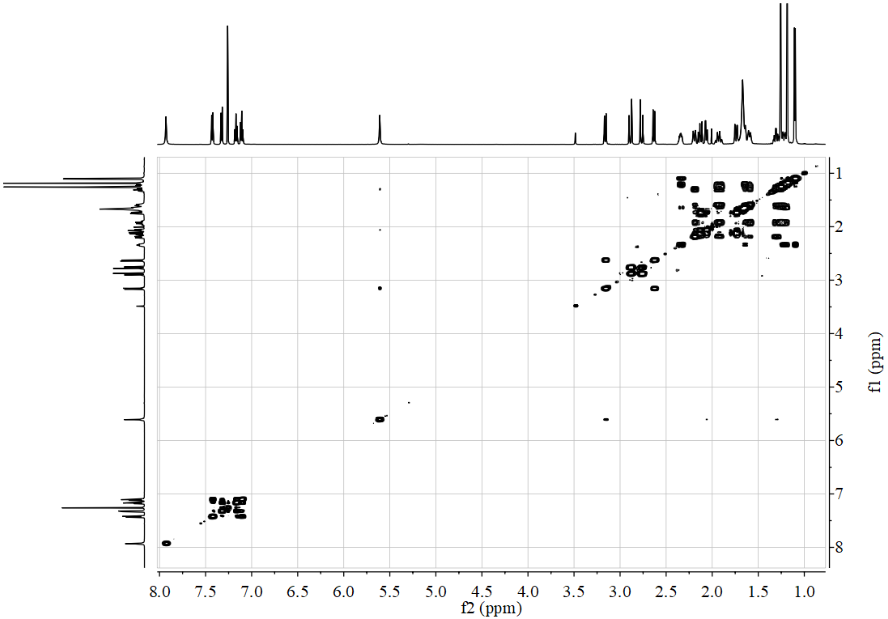

v

**Supplementary Fig. 20-4** | ^1^H-^1^H COSY NMR spectrum of **2** in CDCl_3_.


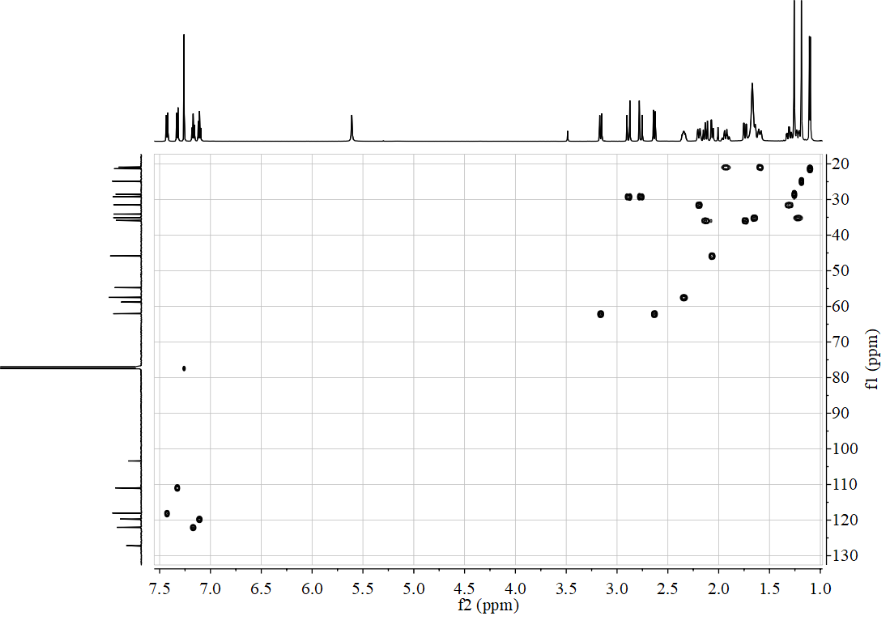

v

**Supplementary Fig. 20-5** | HSQC NMR spectrum of **2** in CDCl_3_.


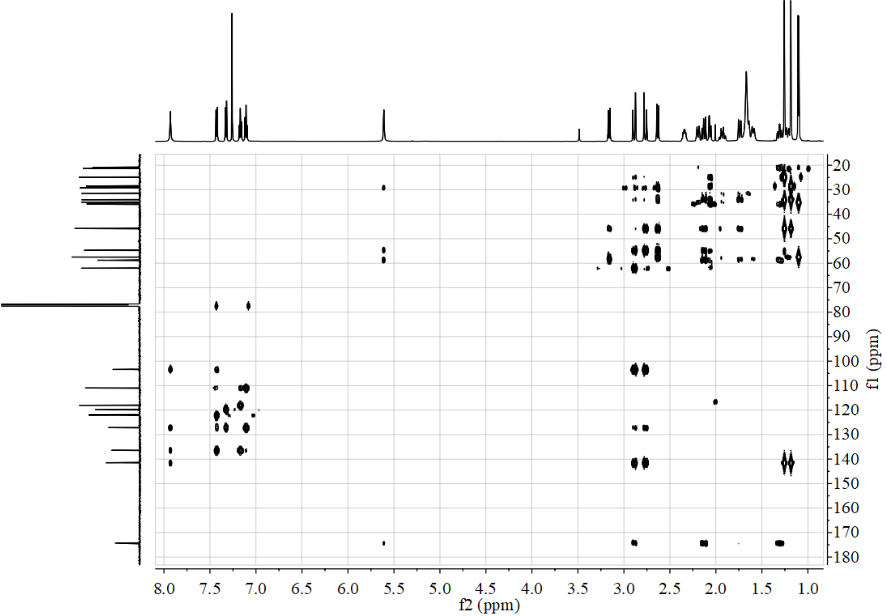

v

**Supplementary Fig. 20-6** | HMBC NMR spectrum of **2** in CDCl_3_.


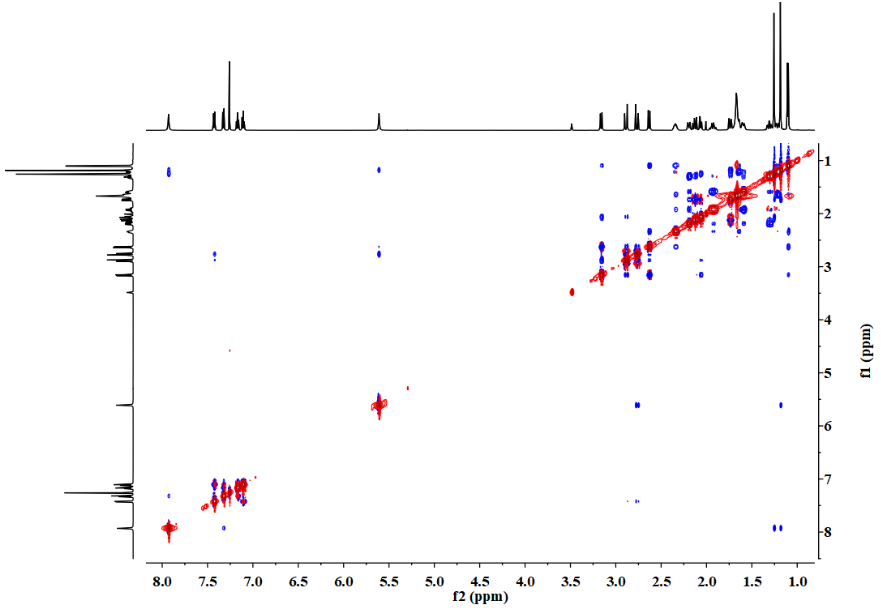

v

**Supplementary Fig. 20-7** | NOESY NMR spectrum of **2** in CDCl_3_.

**
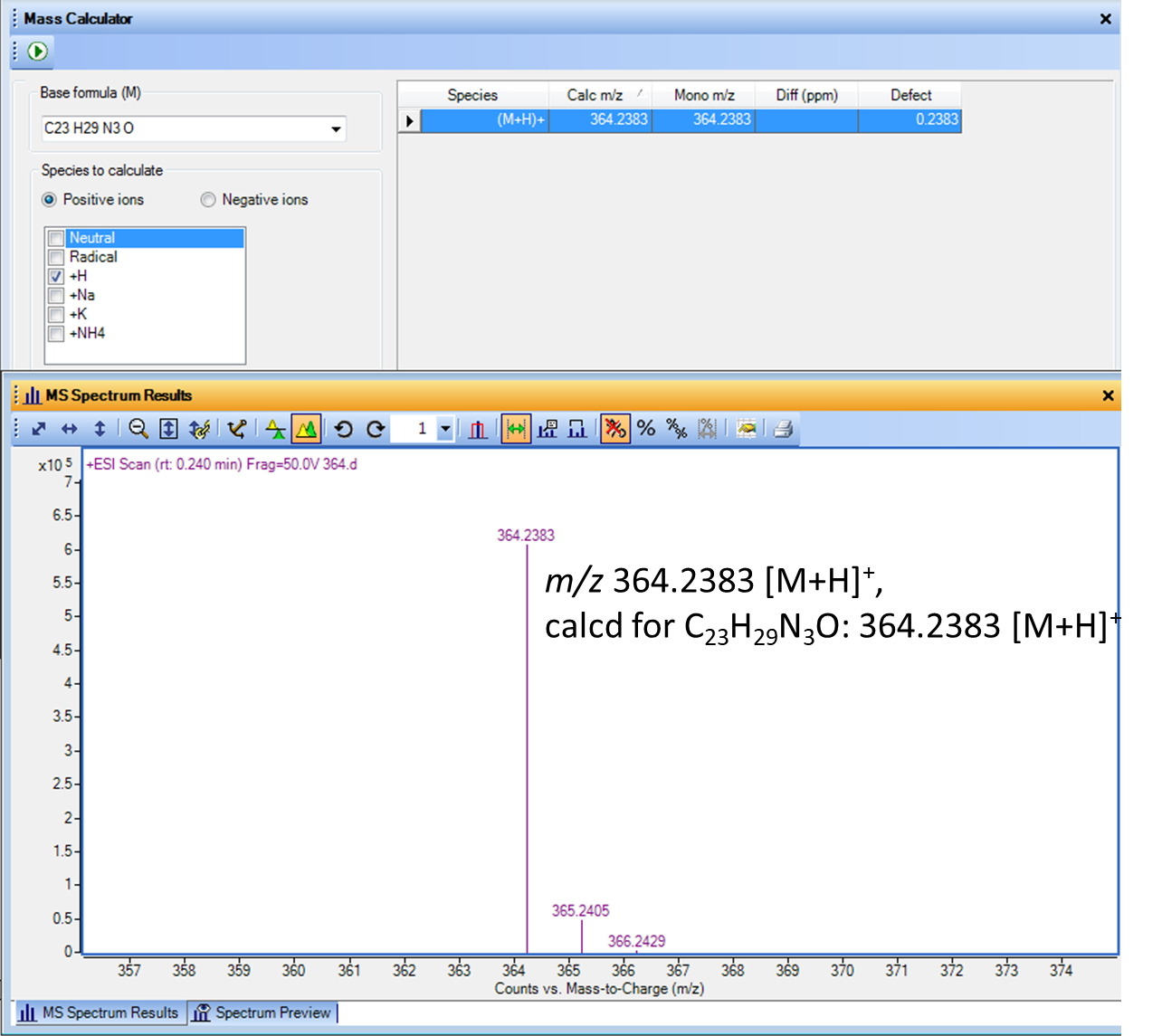
**

v

**Supplementary Fig. 20-8** | HRMS spectrum of **2**.


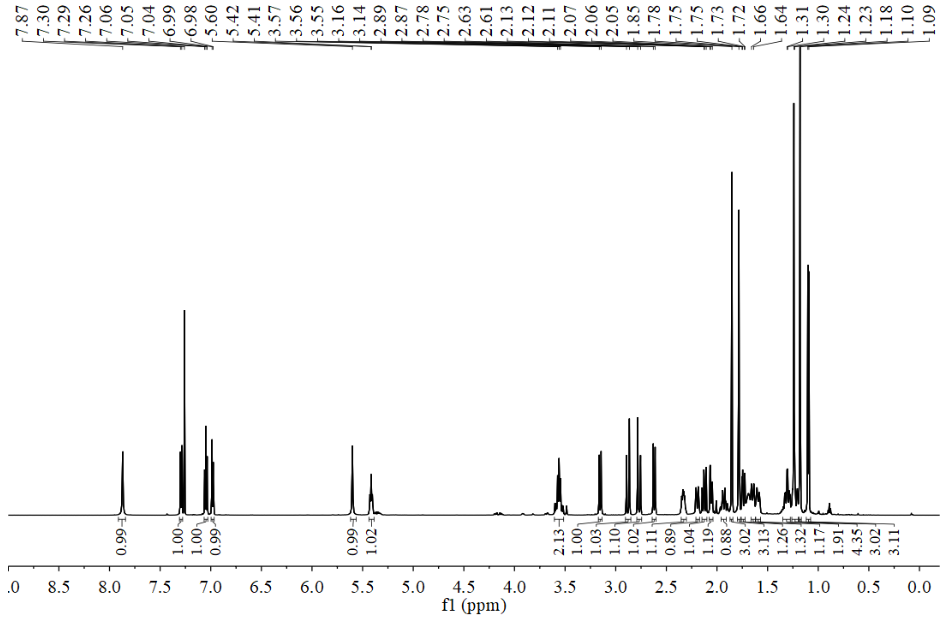

**Supplementary Fig. 21-1** | ^1^H NMR spectrum of **3** in CDCl_3_.


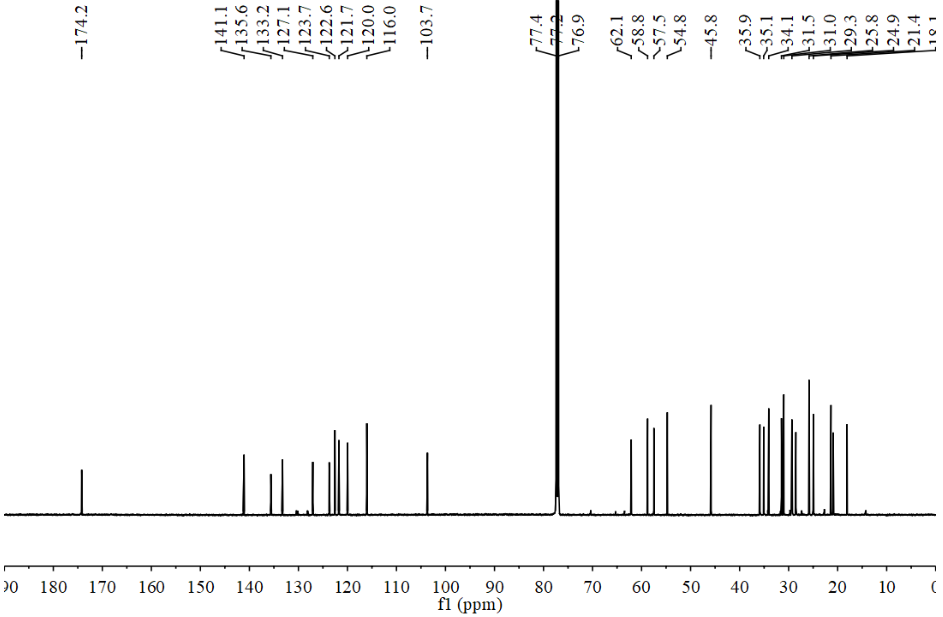

**Supplementary Fig. 21-2** | ^13^C NMR spectrum of **3** in CDCl_3_.


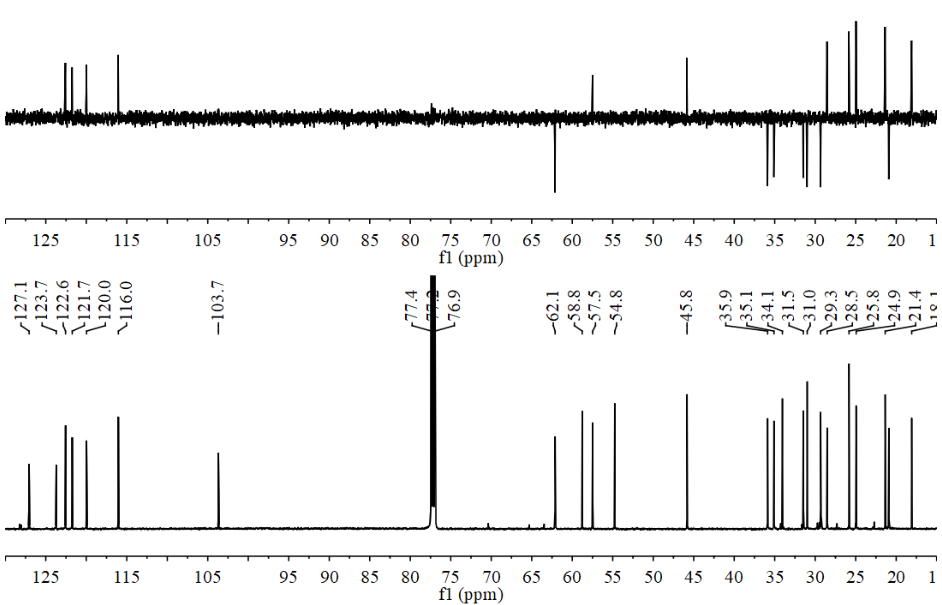

**Supplementary Fig. 21-3** | DEPT135 and ^13^C NMR spectra of **3** in CDCl_3_.


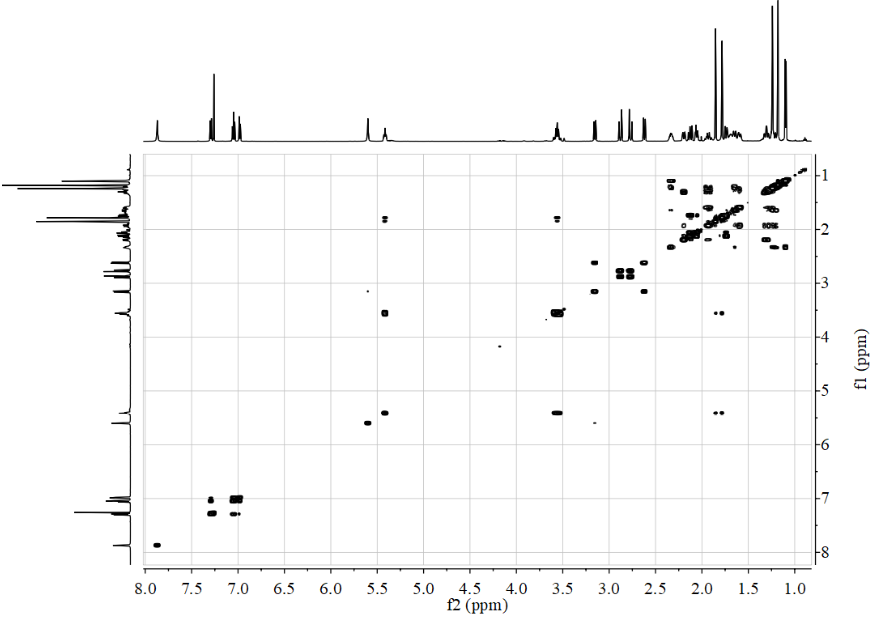

**Supplementary Fig. 21-4** | ^1^H-^1^H COSY NMR spectrum of **3** in CDCl_3_.


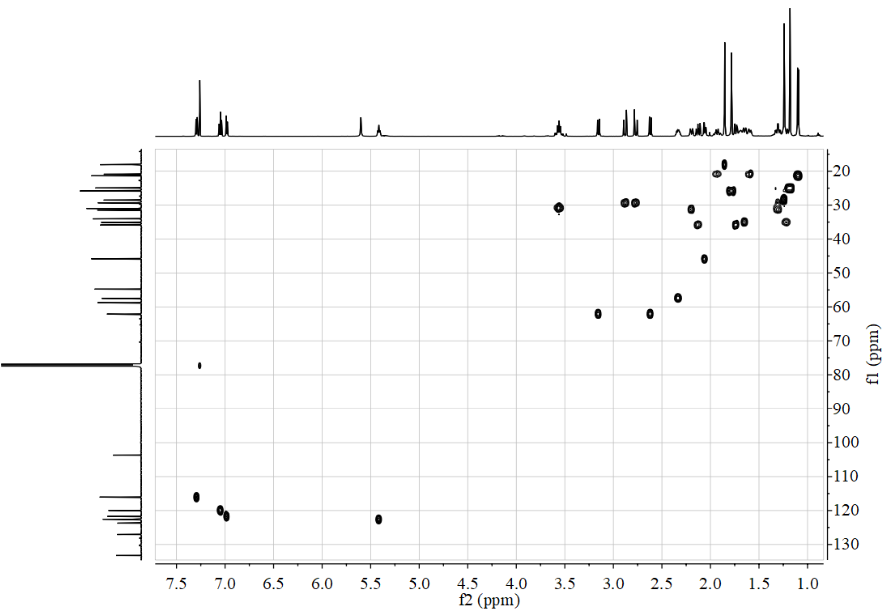

**Supplementary Fig. 21-5** | HSQC NMR spectrum of **3** in CDCl_3_.


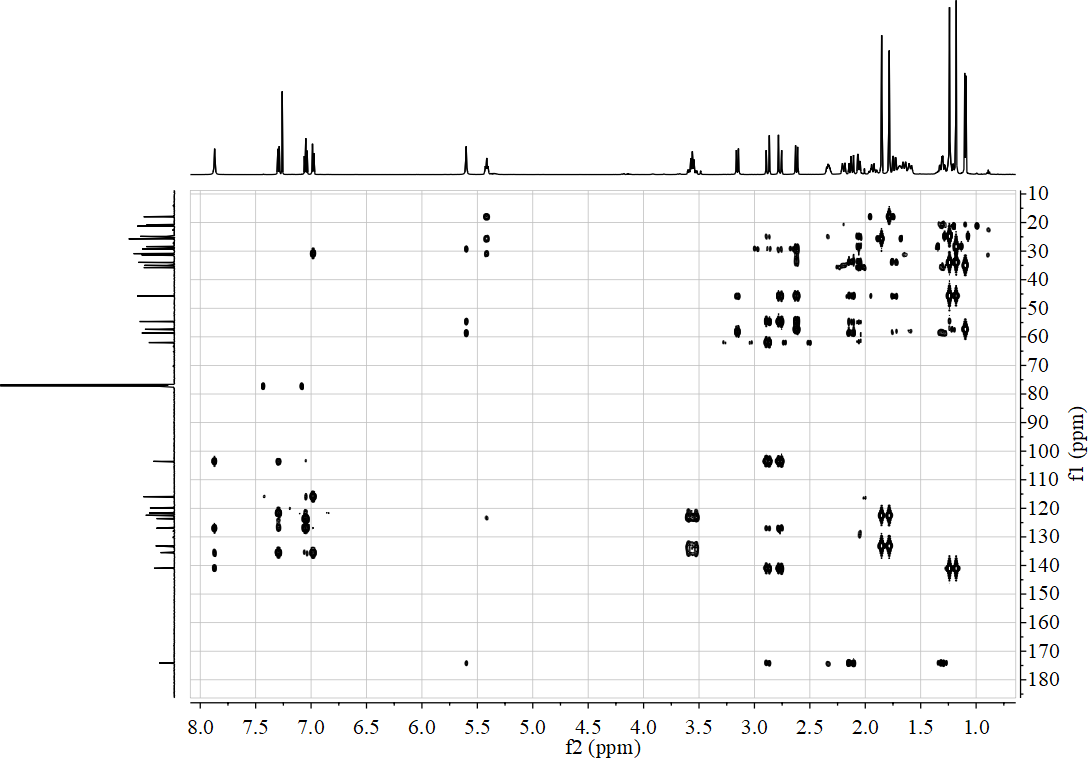

**Supplementary Fig. 21-6** | HMBC NMR spectrum of **3** in CDCl_3_.


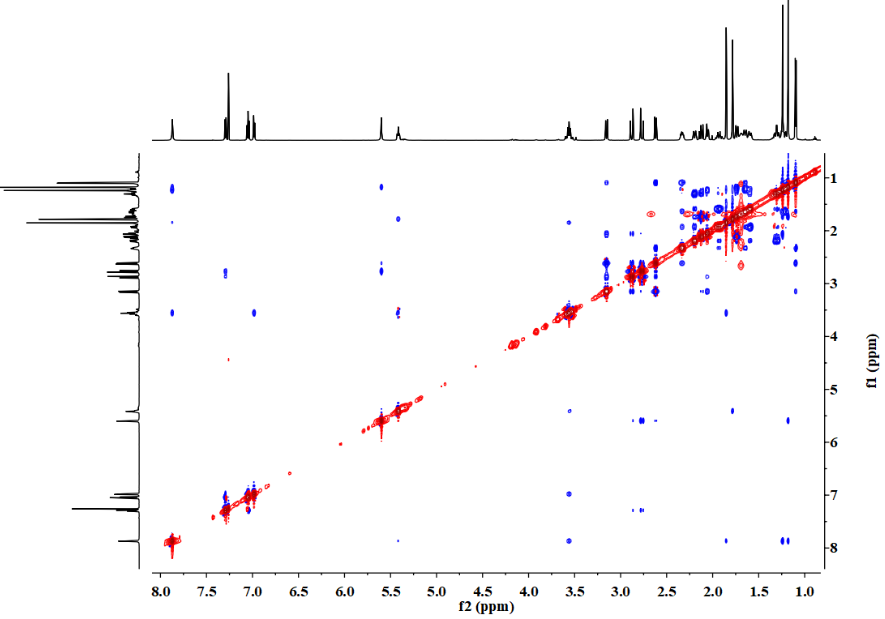

**Supplementary Fig. 21-7** | NOESY NMR spectrum of **3** in CDCl_3_.

**
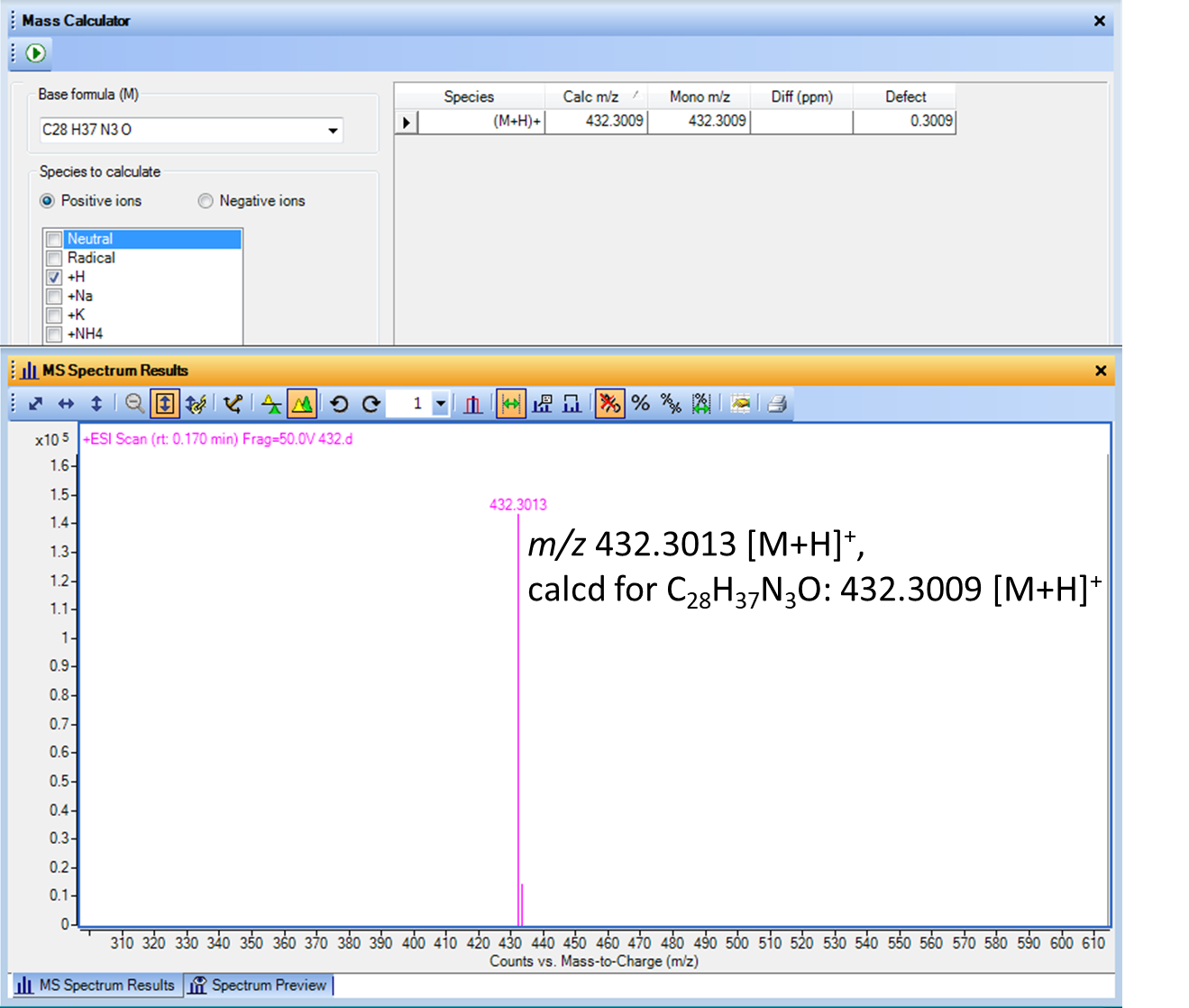
**

**Supplementary Fig. 21-8** | HRMS spectrum of **3**.


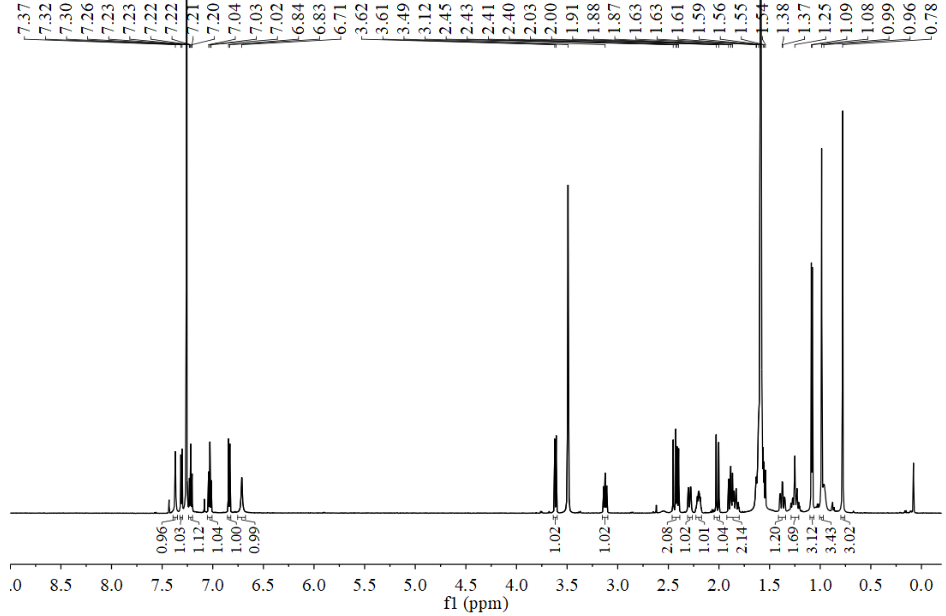

**Supplementary Fig. 22-1** | ^1^H NMR spectrum of **4** in CDCl_3_.


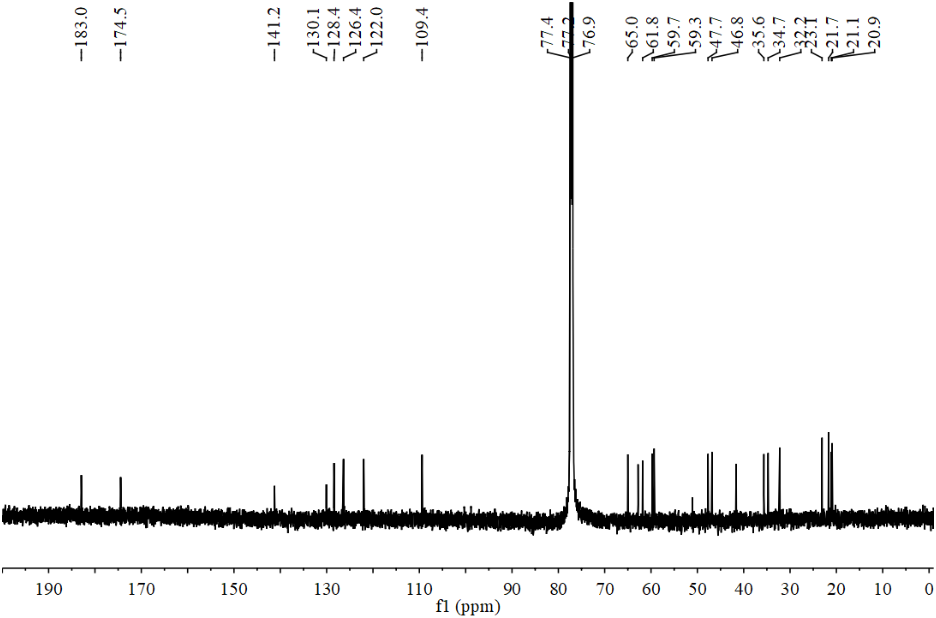

**Supplementary Fig. 22-2** | ^13^C NMR spectrum of **4** in CDCl_3_.


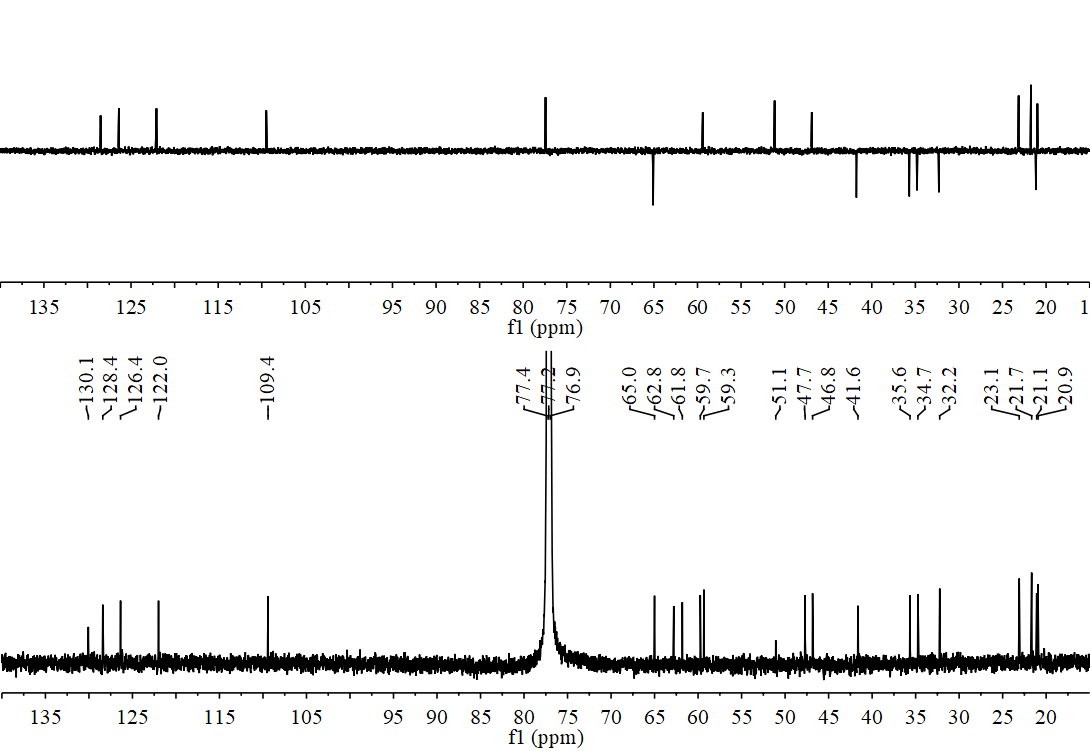

**Supplementary Fig. 22-3** | DEPT135 and ^13^C NMR spectra of **4** in CDCl_3_.


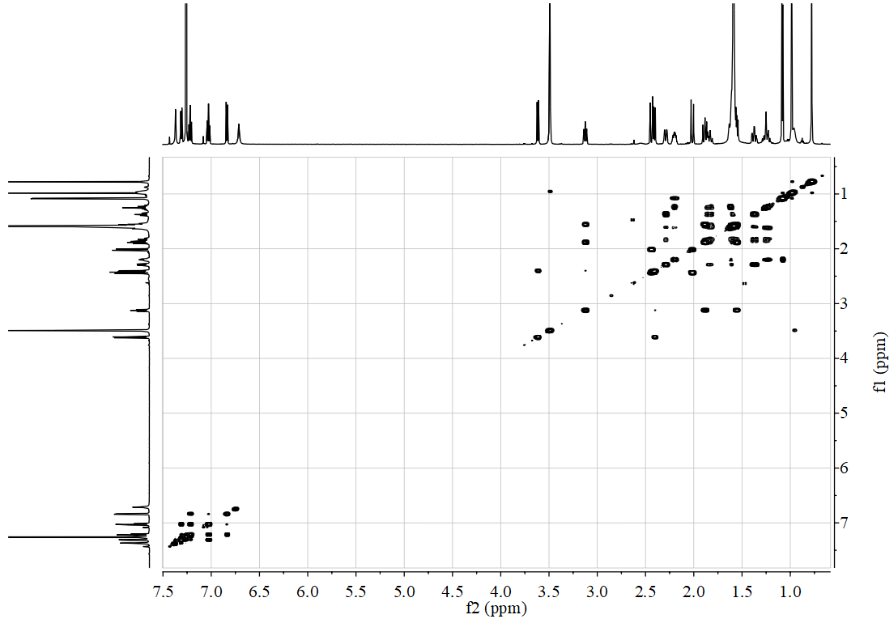

**Supplementary Fig. 22-4** | ^1^H-^1^H COSY NMR spectrum of **4** in CDCl_3_.


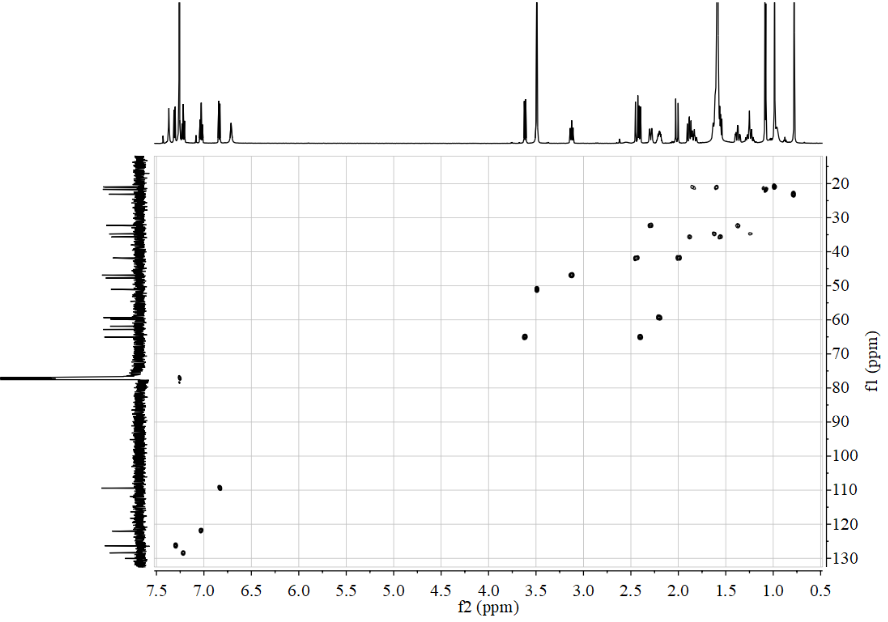

**Supplementary Fig. 22-5** | HSQC NMR spectrum of **4** in CDCl_3_.


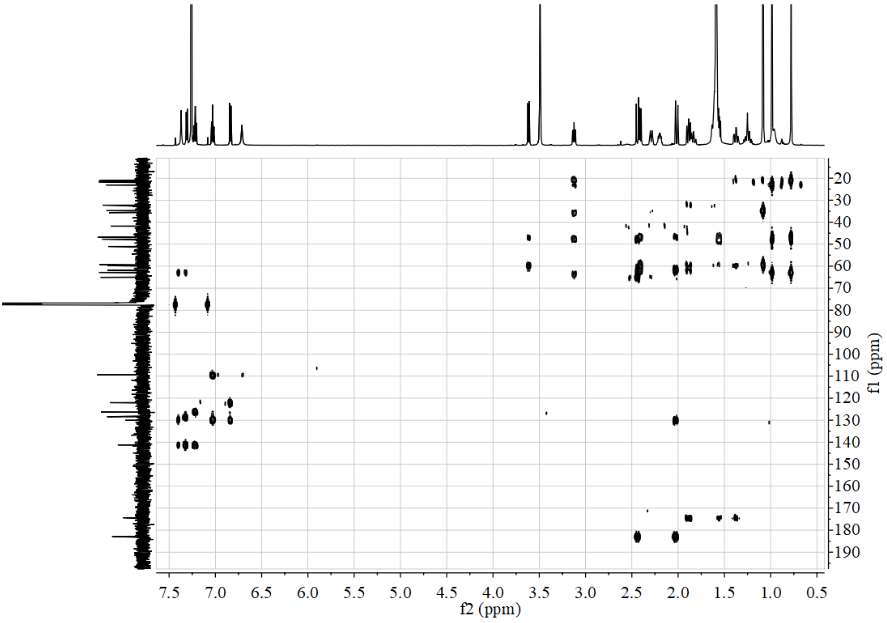

**Supplementary Fig. 22-6** | HMBC NMR spectrum of **4** in CDCl_3_.


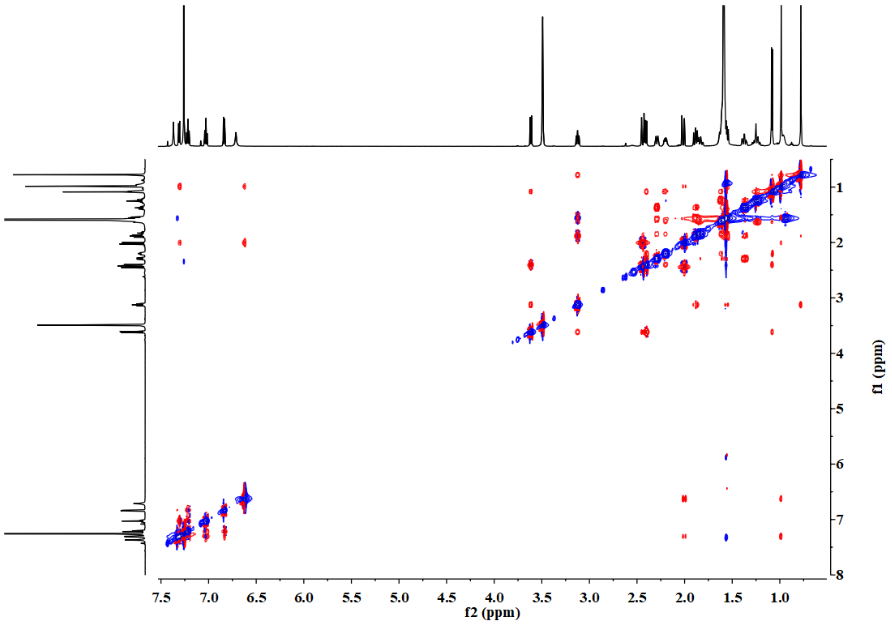

**Supplementary Fig. 22-7** | NOESY NMR spectrum of **4** in CDCl_3_.

**
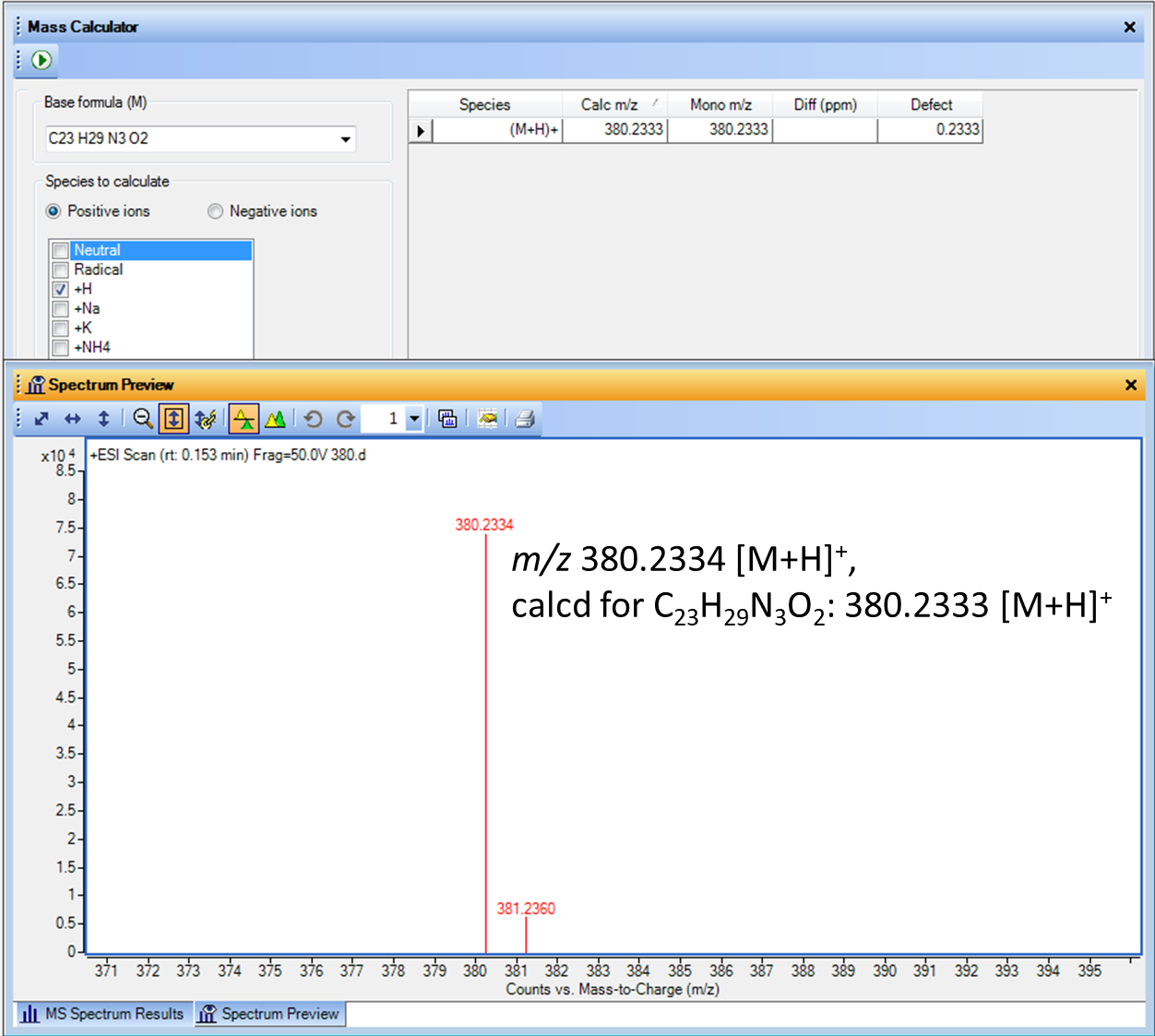
**

**Supplementary Fig. 22-8** | HRMS spectrum of **4**.


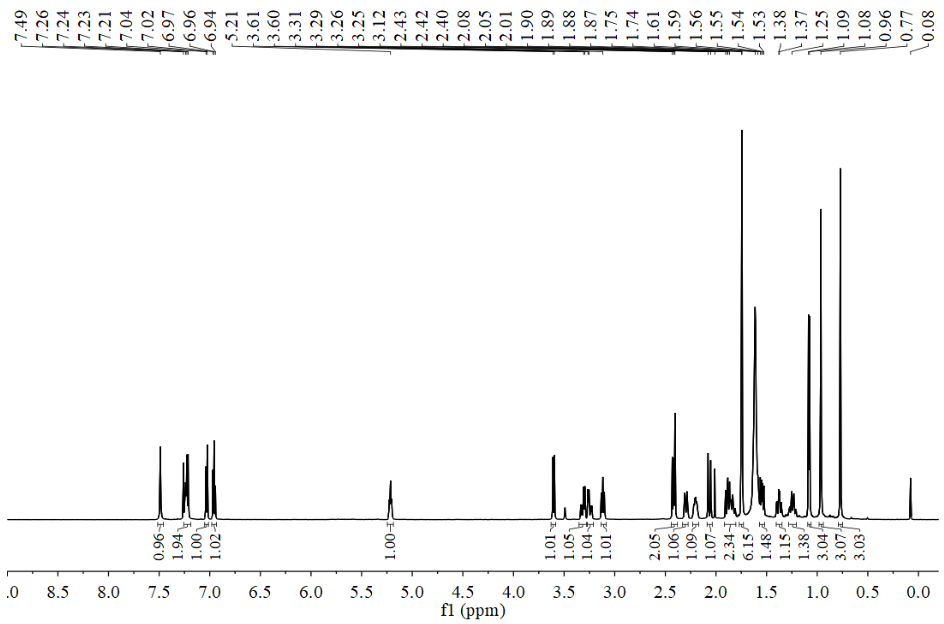

**Supplementary Fig. 23-1** | ^1^H NMR spectrum of **5** in CDCl_3_.


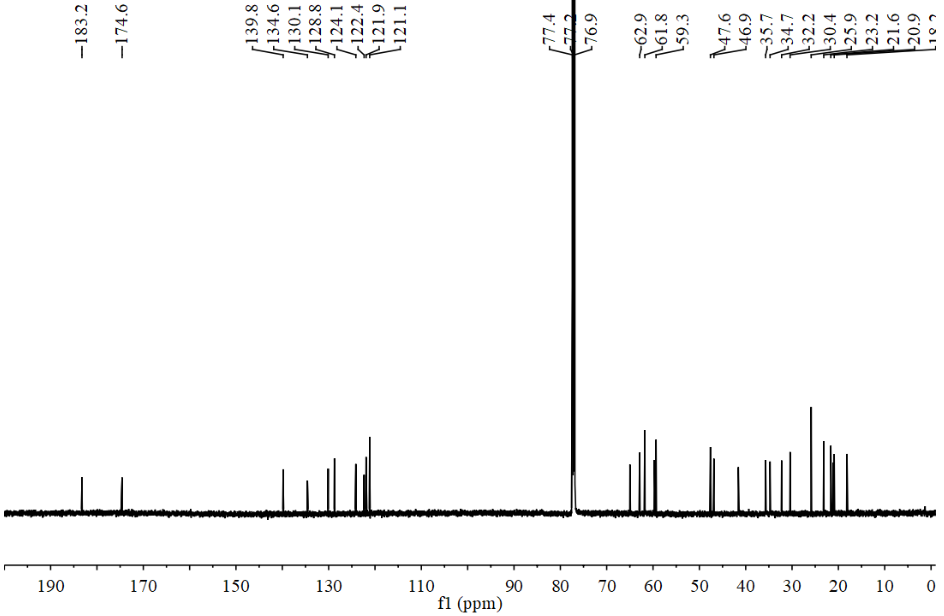

**Supplementary Fig. 23-2** | ^13^C NMR spectrum of **5** in CDCl_3_.


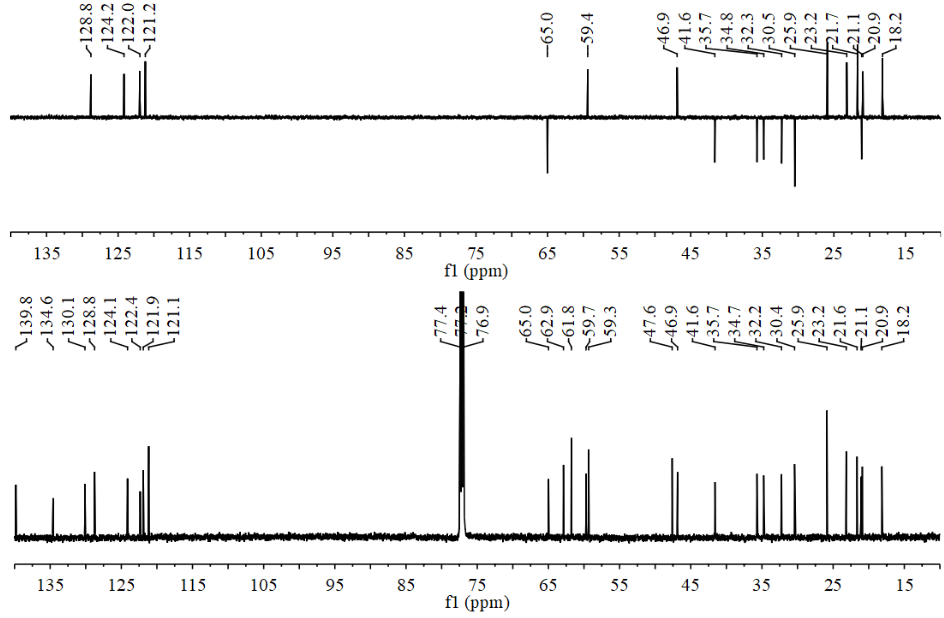

**Supplementary Fig. 23-3** | DEPT135 and ^13^C NMR spectra of **5** in CDCl_3_.


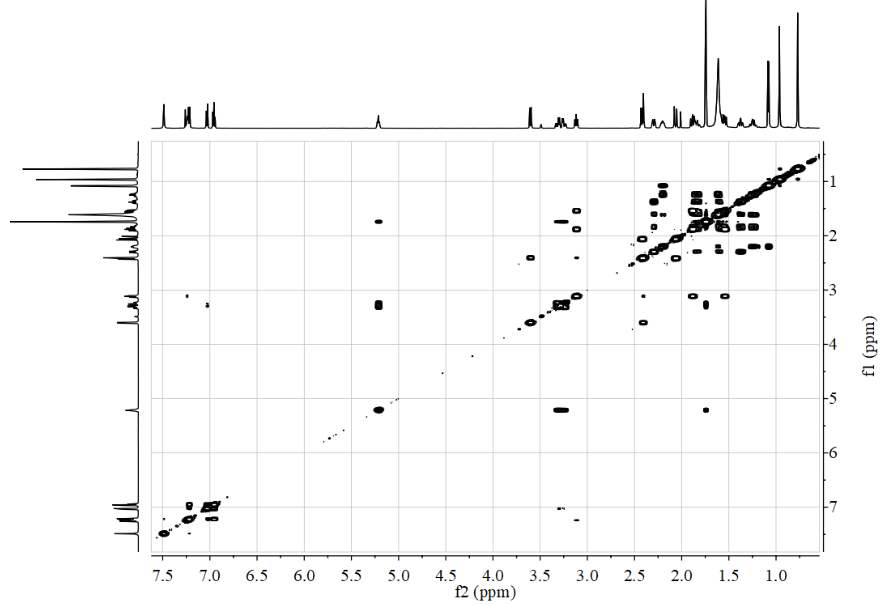

**Supplementary Fig. 23-4** | ^1^H-^1^H COSY NMR spectrum of **5** in CDCl_3_.


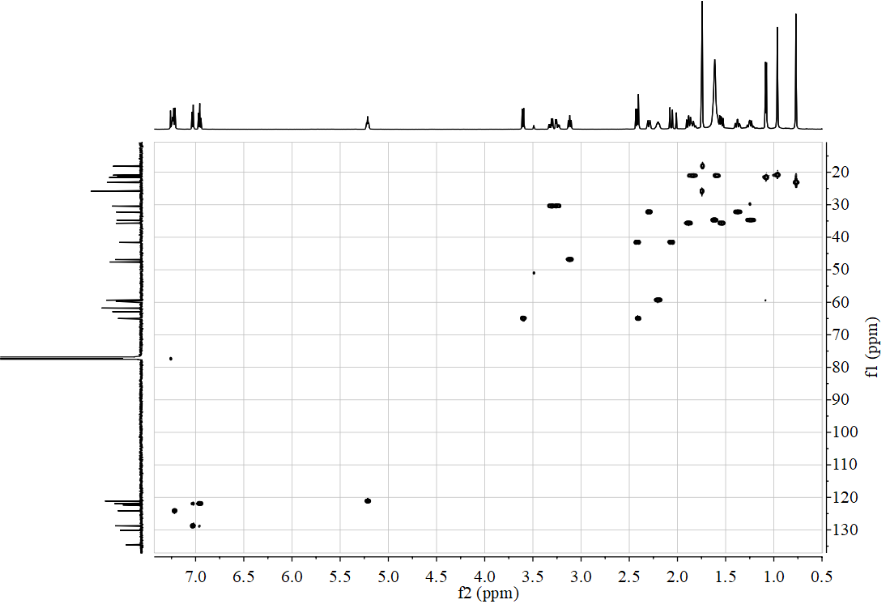

**Supplementary Fig. 23-5** | HSQC NMR spectrum of **5** in CDCl_3_.


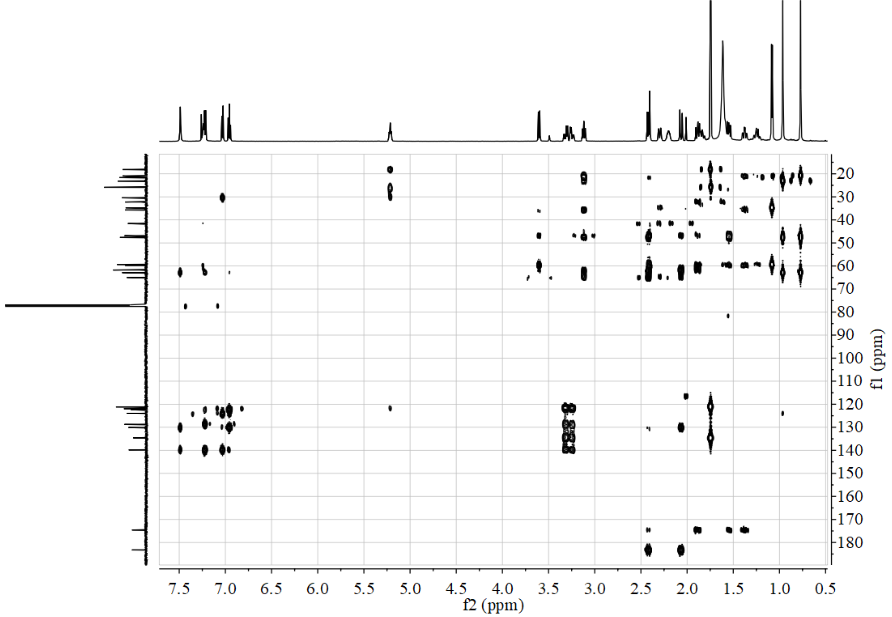

**Supplementary Fig. 23-6** | HMBC NMR spectrum of **5** in CDCl_3_.


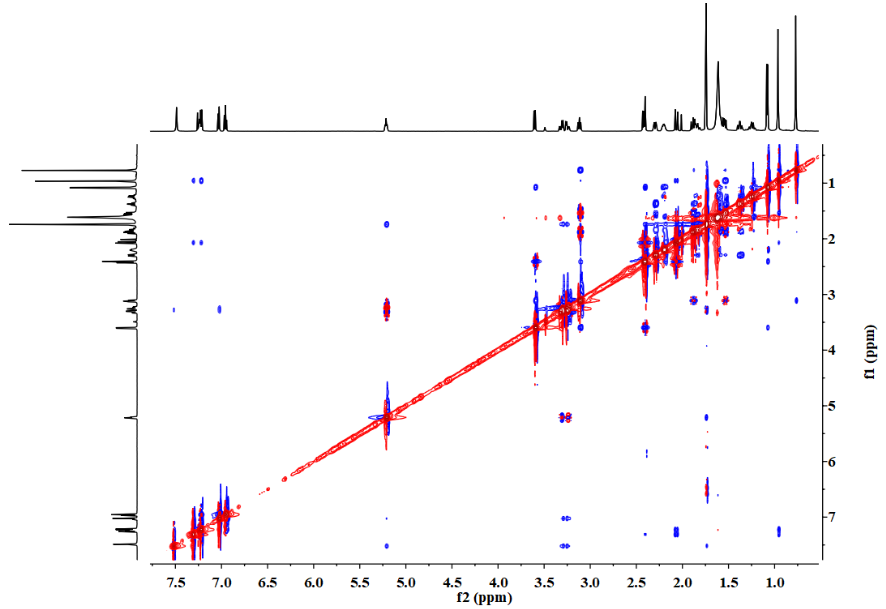

**Supplementary Fig. 23-7** | NOESY NMR spectrum of **5** in CDCl_3_.


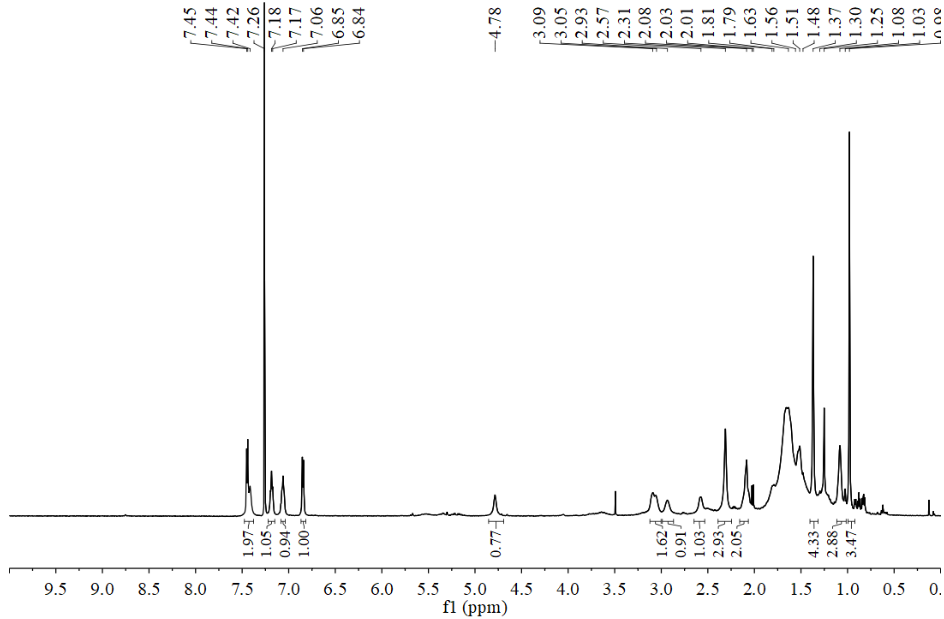

**Supplementary Fig. 24-1** | ^1^H NMR spectrum of **u1** in CDCl_3_.


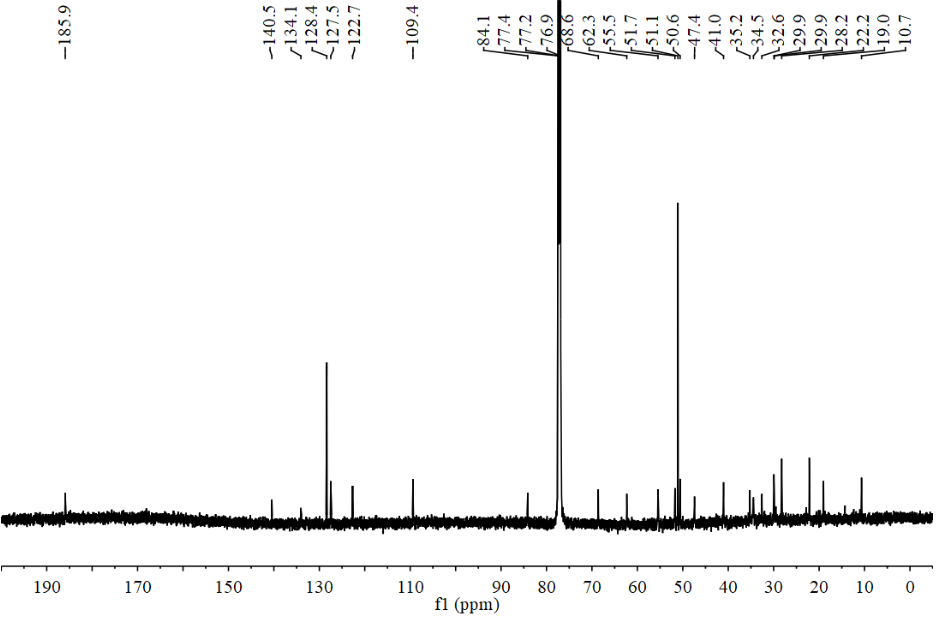

**Supplementary Fig. 24-2** | ^13^C NMR spectrum of **u1** in CDCl_3_.


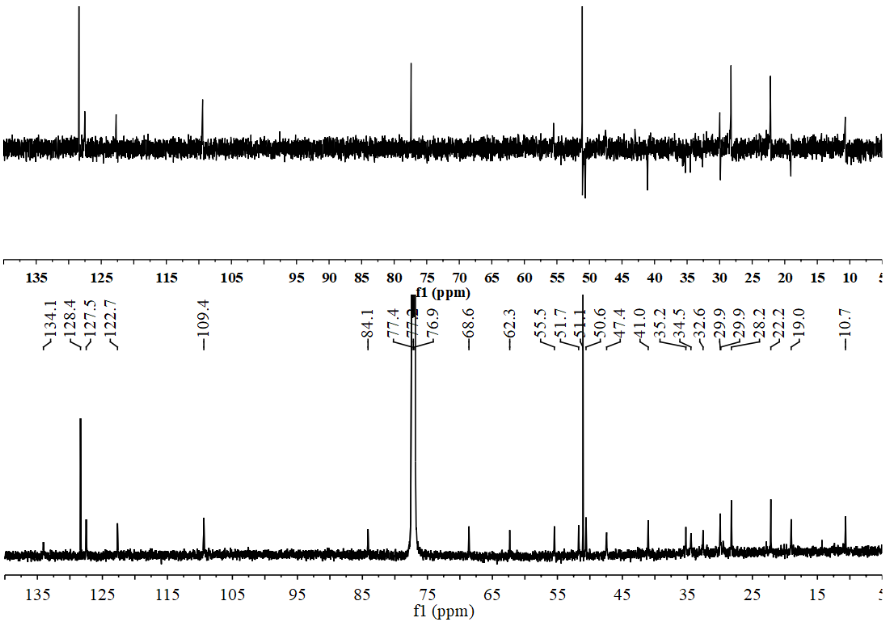

**Supplementary Fig. 24-3** | DEPT135 and ^13^C NMR spectra of **u1** in CDCl_3_.


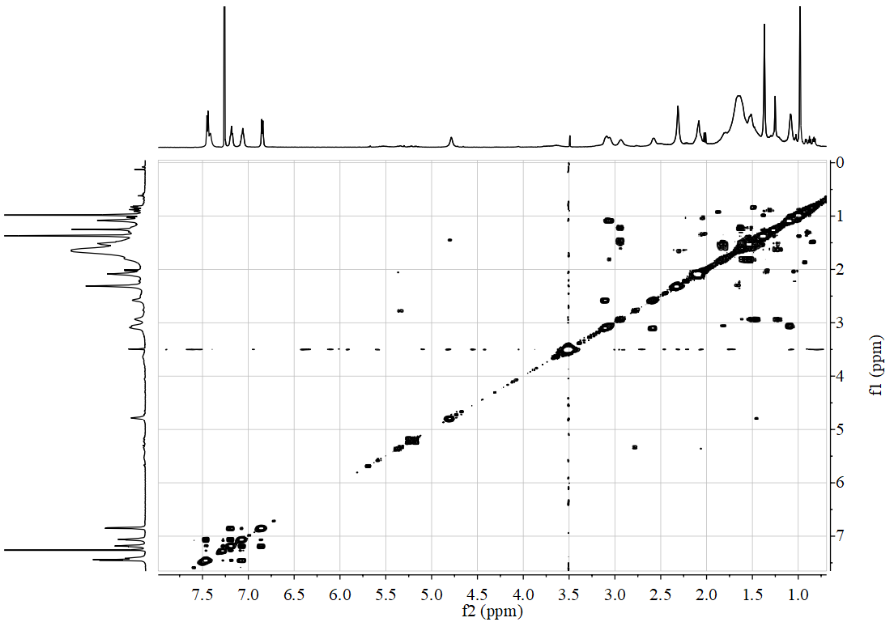

**Supplementary Fig. 24-4** | ^1^H-^1^H COSY NMR spectrum of **u1** in CDCl_3_.


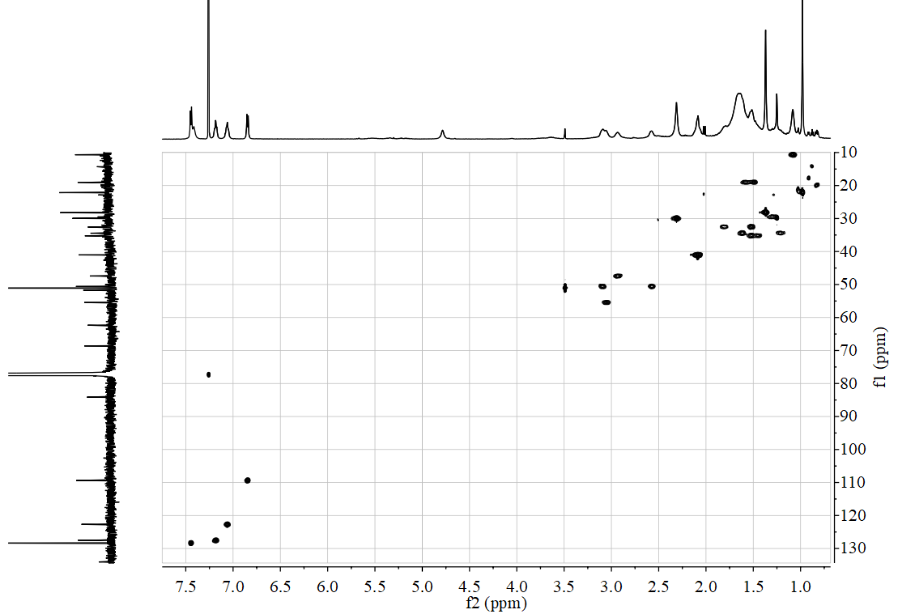

**Supplementary Fig. 24-5** | HSQC NMR spectrum of **3** in CDCl_3_.


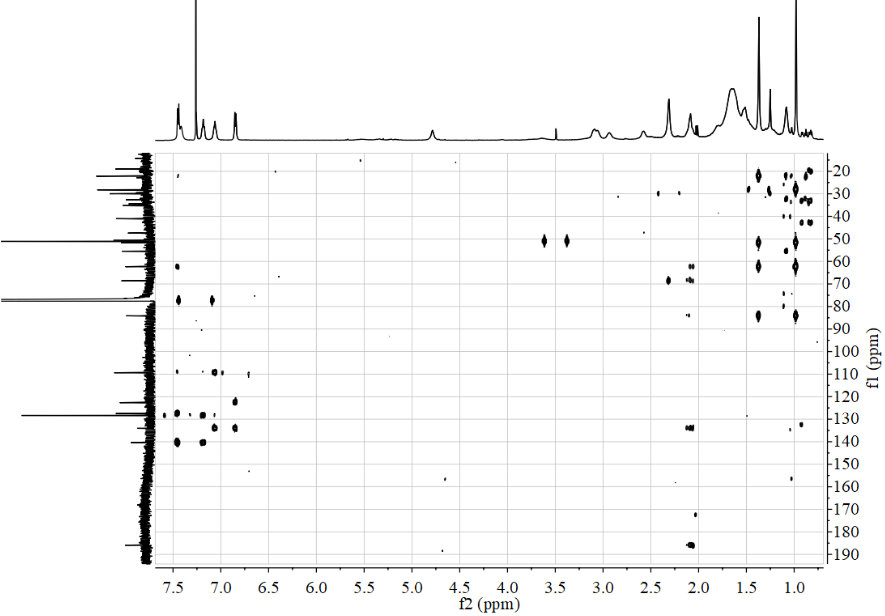

**Supplementary Fig. 24-6** | HMBC NMR spectrum of **u1** in CDCl_3_.

**Energies and Molecular Coordinates of Calculated Structures**.

**Intermediate iii**

Zero-point correction 0.298258 (Hartree/Particle)

Thermal correction to Energy 0.312111

Thermal correction to Enthalpy 0.313055

Thermal correction to Gibbs Free Energy 0.259112

Sum of electronic and zero-point Energies -672.966465

Sum of electronic and thermal Energies -672.952612

Sum of electronic and thermal Enthalpies -672.951668

Sum of electronic and thermal Free Energies -673.005611

**TS iv**

Zero-point correction 0.296704 (Hartree/Particle)

Thermal correction to Energy 0.310433

Thermal correction to Enthalpy 0.311377

Thermal correction to Gibbs Free Energy 0.258135

Sum of electronic and zero-point Energies -672.953718

Sum of electronic and thermal Energies -672.939988

Sum of electronic and thermal Enthalpies -672.939044

Sum of electronic and thermal Free Energies -672.992286

O -1.76335300 -1.07897100 -1.71200600

C -0.71498900 -0.74525900 -0.94438400

C -0.25314400 0.59364600 -0.82910200

C -1.55455200 -0.32841800 0.89872600

C -2.56428100 0.76652900 0.62607600

C -1.94558700 2.07835600 0.16918000

C -1.09703400 1.78784800 -1.08989700

C -0.52480300 -0.06952300 1.96522800

C -2.19269500 -1.69328500 1.06143500

H -3.28349600 0.40917000 -0.11949200

C 1.10456200 0.51422900 -0.42282100

C 1.44797800 -0.85966600 -0.35231300

N 0.34716900 -1.60148100 -0.70342500

C 2.05150700 1.49800700 -0.07442500

C 3.30621600 1.08451300 0.31214800

C 3.62886600 -0.29199500 0.37641500

C 2.71704900 -1.28076800 0.06174900

H -1.76075800 1.58349300 -1.93610200

H -1.01170300 -0.23935600 2.93321800

H 0.30300000 -0.78337200 1.89979600

H -0.12149600 0.94287800 1.95377400

H -2.88871600 -1.93155500 0.25578400

H -1.44829500 -2.48494400 1.17596700

H -2.77907600 -1.65607400 1.98799500

H 0.31620000 -2.61214800 -0.76534700

H 1.79207000 2.55083100 -0.11755400

H 4.06093600 1.81569300 0.57747700

H 4.62741700 -0.58018600 0.68742900

H -0.46557800 2.64272600 -1.34183600

H -2.71958900 2.81190600 -0.06702700

H -1.31298500 2.50859200 0.95137600

H -3.11990100 0.90756300 1.56379400

H 2.97016600 -2.33247300 0.12414900

H -1.93610600 -2.03588500 -1.69146900

**Intermediate v**

Zero-point correction 0.299563 (Hartree/Particle)

Thermal correction to Energy 0.313397

Thermal correction to Enthalpy 0.314341

Thermal correction to Gibbs Free Energy 0.259973

Sum of electronic and zero-point Energies -672.996868

Sum of electronic and thermal Energies -672.983033

Sum of electronic and thermal Enthalpies -672.982089

Sum of electronic and thermal Free Energies -673.036458

**Intermediate vi**

Zero-point correction 0.298898 (Hartree/Particle)

Thermal correction to Energy 0.312186

Thermal correction to Enthalpy 0.313130

Thermal correction to Gibbs Free Energy 0.260482

Sum of electronic and zero-point Energies -672.985989

Sum of electronic and thermal Energies -672.972701

Sum of electronic and thermal Enthalpies -672.971756

Sum of electronic and thermal Free Energies -673.024405

**TS vii**

Zero-point correction 0.294560 (Hartree/Particle)

Thermal correction to Energy 0.307394

Thermal correction to Enthalpy 0.308338

Thermal correction to Gibbs Free Energy 0.265020

Sum of electronic and zero-point Energies -672.940791

Sum of electronic and thermal Energies -672.927957

Sum of electronic and thermal Enthalpies -672.927013

Sum of electronic and thermal Free Energies -672.986331

C 0.71258000 0.33273200 -0.02289900

C -0.07872100 -0.87635900 -0.30797600

C 2.20505600 0.57950600 -0.31427300

C 2.98861200 -0.11730700 0.82939800

C 2.16788400 -1.29553700 1.35885600

C 2.52008500 2.07622800 -0.34147800

C 2.52851000 -0.04490400 -1.67514600

H 3.98242200 -0.44995900 0.47500800

C -1.49006500 -0.51546900 -0.19050100

C -1.53341400 0.88018100 0.12466700

N -0.20953600 1.37716500 0.19570100

C -2.68462600 -1.23485000 -0.33750900

C -3.88494500 -0.55577500 -0.17348500

C -3.91395900 0.82282600 0.14038500

C -2.74638000 1.56127900 0.29723300

H 2.31240900 2.57935200 0.61074400

H 3.58931300 2.24097600 -0.54578600

H 1.97498100 2.60687600 -1.13331400

H 1.92336800 0.38545100 -2.48269900

H 3.58047400 0.11988000 -1.94510400

H 2.36684900 -1.13274200 -1.68537500

H 0.04408700 2.32060600 0.48266400

H -2.66741200 -2.30066800 -0.57372500

H -4.83118800 -1.09034400 -0.28496600

H -4.88394900 1.31589300 0.26344000

H 2.53314100 -1.61722800 2.35786500

H 3.19638000 0.60150400 1.64717700

H -2.76856800 2.62305600 0.54401200

O 0.43519600 -1.93495900 -0.95528800

H -0.22449400 -2.70568600 -1.00474500

H 2.28637400 -2.18244500 0.69910500

C 0.71620500 -0.88493100 1.44060500

H 0.49165900 -0.11543500 2.19685800

H 0.03841400 -1.72529200 1.68397000

**Intermediate vii**

Zero-point correction 0.298766 (Hartree/Particle)

Thermal correction to Energy 0.312921

Thermal correction to Enthalpy 0.313865

Thermal correction to Gibbs Free Energy 0.260555

Sum of electronic and zero-point Energies -672.981692

Sum of electronic and thermal Energies -672.967538

Sum of electronic and thermal Enthalpies -672.966594

Sum of electronic and thermal Free Energies -673.019964

**Truncated reactant of the C2/C3 hydroxylation:**

Zero-point correction= 0.527804 (Hartree/Particle)

Thermal correction to Energy= 0.558970

Thermal correction to Enthalpy= 0.559914

Thermal correction to Gibbs Free Energy= 0.468460

Sum of electronic and zero-point Energies= -1581.748516

Sum of electronic and thermal Energies= -1581.717350

Sum of electronic and thermal Enthalpies= -1581.716406

Sum of electronic and thermal Free Energies= -1581.807860

**Truncated C2-hydroxyl intermediate TS:**

Zero-point correction= 0.526535 (Hartree/Particle)

Thermal correction to Energy= 0.557345

Thermal correction to Enthalpy= 0.558289

Thermal correction to Gibbs Free Energy= 0.466606

Sum of electronic and zero-point Energies= -1581.692757

Sum of electronic and thermal Energies= -1581.661947

Sum of electronic and thermal Enthalpies= -1581.661003

Sum of electronic and thermal Free Energies= -1581.752686

**Structure of truncated C2-hydroxyl intermediate TS**

C -1.49051700 3.02373300 0.14104500

C -0.16819700 4.87812100 -0.18269200

C 0.65963000 2.71539000 -1.10290000

C -0.38257800 2.03155800 -0.21307900

C -2.08913900 0.29922500 -0.43166400

C -2.42674400 -1.03102000 -0.67075500

C -3.61263100 -1.57958900 -0.18913800

C -3.96457600 -3.01371900 -0.48761600

C -4.46717100 -0.78460300 0.59696500

C -5.74327500 -1.35764800 1.15687700

C -4.12215500 0.53867500 0.85099600

C -2.95452000 1.09137600 0.32873100

N -1.36523900 4.30875000 0.21452600

N -2.65477600 2.44683500 0.50610200

N 0.76774200 4.05310100 -0.86773200

N -0.88487300 0.85324000 -0.91836800

O 0.10285700 6.04543700 -0.03922800

O 1.37277200 2.11002400 -1.88292300

N 1.55668200 -2.13719800 1.40732300

C 3.26500000 0.23056000 2.55102700

C 3.56604800 -0.57002900 1.27994300

C 0.80211500 -2.86400800 0.52334500

C 2.94349800 -0.76098200 -1.72950700

C 4.29998300 -0.29297100 -1.17647000

C 4.15340400 0.36536400 0.20003700

C 4.54497200 -1.70711500 1.61452000

C 2.30296000 -1.18084900 0.69829800

C 2.17387700 -1.49109000 -0.68276300

C 1.19137400 -2.51197200 -0.79865500

C 0.60598600 -3.16772900 -1.89906400

C -0.35208600 -4.13289600 -1.66167400

C -0.74768100 -4.44703200 -0.34153400

C -0.19062400 -3.82092600 0.75985100

H -0.75575700 0.86205400 -1.92538800

H 1.54127100 4.54782700 -1.29807700

H -3.35906200 3.07068000 0.88343800

H -4.77966500 1.16281200 1.45226600

H -1.73878400 -1.64575800 -1.24946900

H -6.26838200 -0.62315200 1.77206800

H -6.42339000 -1.67483100 0.35799400

H -5.54718700 -2.23904100 1.77775200

H -3.25582500 -3.44605900 -1.19782500

H -3.94796600 -3.62914700 0.42062400

H -4.97039600 -3.09927400 -0.91374900

H -1.51075800 -5.20401500 -0.18735900

H -0.50120700 -4.07225400 1.76862100

H -0.81281900 -4.65773300 -2.49220000

H 0.90811300 -2.91420500 -2.91101600

H 1.27917100 -1.97864000 2.36652100

H 2.35848400 0.11629100 -2.03631000

H 3.07248600 -1.39603700 -2.61314400

H 4.74327600 0.42164500 -1.87634600

H 4.98771400 -1.14486300 -1.11755600

H 3.49933600 1.23938900 0.10737600

H 5.13127900 0.71693700 0.55085400

H 5.49540000 -1.28727100 1.96092600

H 4.14492200 -2.34439600 2.40916500

H 4.74472100 -2.34440500 0.74771200

H 4.19471100 0.66404600 2.93486200

H 2.55114600 1.03327000 2.35352300

H 2.85876700 -0.41305000 3.34137300

O 0.14179200 1.70663200 1.00158100

O 1.26879500 0.24660500 0.60018400

H 0.55222600 -0.02245300 -0.02125700

**Truncated C3-hydroxyl intermediate TS:**

Zero-point correction= 0.527608 (Hartree/Particle)

Thermal correction to Energy= 0.558515

Thermal correction to Enthalpy= 0.559459

Thermal correction to Gibbs Free Energy= 0.467213

Sum of electronic and zero-point Energies= -1581.697361

Sum of electronic and thermal Energies= -1581.666454

Sum of electronic and thermal Enthalpies= -1581.665509

Sum of electronic and thermal Free Energies= -1581.757756

**Structure of Truncated C2-hydroxyl intermediate TS:**

C -3.08244400 -1.47996900 -0.23105300

C -3.19032300 -3.73727700 0.18836200

C -1.24768300 -2.50394300 1.14891700

C -1.63933500 -1.33950300 0.24248500

C -1.90559700 1.02272700 0.33527400

C -1.28211600 2.25706500 0.49884800

C -1.71455600 3.38751300 -0.19553600

C -1.02549400 4.71070200 0.01672500

C -2.78881400 3.28022900 -1.09341600

C -3.27051400 4.48429400 -1.86045900

C -3.40542700 2.04103100 -1.26683200

C -2.97906000 0.92164000 -0.56406500

N -3.75714700 -2.57995400 -0.31628100

N -3.61175300 -0.32353300 -0.68642200

N -1.98281400 -3.63105000 0.93427400

N -1.55908500 -0.12669900 1.03800800

O -3.68767100 -4.83238500 0.07473300

O -0.31392600 -2.44272600 1.92961200

N 2.89589100 1.33519900 -0.63410400

C 2.47271700 -0.52396400 -3.06798100

C 3.37656200 -0.82600800 -1.86002900

C 2.43147100 1.76617800 0.61584400

C 2.78637300 -1.93338000 0.99259900

C 3.70031000 -2.60845800 -0.04239900

C 3.24218300 -2.31875200 -1.47526800

C 4.83562100 -0.47335500 -2.20152700

C 2.93450100 -0.01432500 -0.67961500

C 2.41407500 -0.54670300 0.53058800

C 2.14971900 0.61772100 1.37781700

C 1.67756100 0.75415200 2.68315400

C 1.51576300 2.03735900 3.19677800

C 1.81249600 3.16939800 2.42314500

C 2.27085500 3.05360200 1.11358800

H -0.68932200 -0.08573500 1.56114900

H -1.69663900 -4.48149800 1.40325400

H -4.51534800 -0.39272200 -1.13645900

H -4.23575900 1.94452800 -1.96250600

H -0.45615000 2.33381000 1.20303800

H -4.10486100 4.22435600 -2.51522900

H -3.60814300 5.28035800 -1.18775400

H -2.47594400 4.90946500 -2.48360700

H -0.23255200 4.61824800 0.76246900

H -0.57897400 5.08610600 -0.91140500

H -1.72741200 5.47730000 0.36233800

H 1.67697500 4.15745800 2.85044800

H 2.49184600 3.93034800 0.51354900

H 1.15064200 2.16785400 4.20948800

H 1.43293800 -0.12684500 3.26956900

H 3.09426300 1.94262900 -1.41572000

H 1.86323100 -2.49978600 1.14453200

H 3.29386700 -1.86624000 1.96022000

H 3.71362600 -3.68758100 0.13058600

H 4.73105800 -2.25695600 0.09312000

H 2.19257500 -2.61204000 -1.58034100

H 3.82667700 -2.90507900 -2.19277000

H 5.15265700 -1.04969100 -3.07516200

H 4.94597900 0.58859800 -2.44461200

H 5.51222600 -0.70083300 -1.37369100

H 2.76690100 -1.16146400 -3.90717200

H 1.42851000 -0.73020600 -2.81997900

H 2.56657300 0.51772500 -3.39541700

O -0.90880100 -1.35479000 -0.91015800

O 0.85217200 -0.92945100 -0.31806100

H 0.51766900 -0.05286400 -0.57492800

**Supplementary References**

1. Bian, Z., Marvin, C.C. & Martin, S.F. Enantioselective total synthesis of (-)-citrinadin A and revision of its stereochemical structure. *J. Am. Chem. Soc.* **135**, 10886-10889 (2013).

2. Holm, L. & Rosenström, P. Dali server: conservation mapping in 3D. *Nucleic Acids Res.* **38**, W545-W549 (2010).

3. Li, S. et al. Biochemical characterization of NotB as an FAD-dependent oxidase in the biosynthesis of notoamide indole alkaloids. *J. Am. Chem. Soc.* **134**, 788-791 (2012).

4. Fraley, A.E. et al. Molecular basis for spirocycle formation in the paraherquamide biosynthetic pathway. *J. Am. Chem. Soc.* **142**, 2244-2252 (2020).

5. Fraley, A.E. et al. Flavin-dependent monooxygenases NotI and NotI' mediate spiro-oxindole formation in biosynthesis of the notoamides. *ChemBioChem* **21**, 2449-2454 (2020).

6. Ye, Y. et al. Fungal-derived brevianamide assembly by a stereoselective semipinacolase. *Nat. Catal.* **3**, 497-506 (2020).

7. Tsunematsu, Y. et al. Distinct mechanisms for spiro-carbon formation reveal biosynthetic pathway crosstalk. *Nat. Chem. Biol.* **9**, 818-825 (2013).

8. Chen, M., Liu, C. & Tang, Y. Discovery and biocatalytic application of a PLP-dependent amino acid γ-substitution enzyme that catalyzes C–C bond formation. *J. Am. Chem. Soc.* **142**, 10506-10515 (2020).

9. Li, S. et al. Comparative analysis of the biosynthetic systems for fungal bicyclo[2.2.2]diazaoctane indole alkaloids: the (+)/(-)-notoamide, paraherquamide and malbrancheamide pathways. *Med. Chem. Comm.* **3**, 987-996 (2012).

10. Li, T. et al. Comparative transcriptome analysis of *Penicillium citrinum* cultured with different carbon sources identifies genes involved in citrinin biosynthesis. *Toxins* **9**, 1-18 (2017).

11. Dan, Q. et al. Fungal indole alkaloid biogenesis through evolution of a bifunctional reductase/Diels-Alderase. *Nat. Chem.* **11**, 972-980 (2019).
